# Identification of Human Alanine–Glyoxylate Aminotransferase Ligands as Pharmacological Chaperones for Variants Associated with Primary Hyperoxaluria Type 1

**DOI:** 10.1021/acs.jmedchem.2c00142

**Published:** 2022-07-13

**Authors:** Silvia Grottelli, Giannamaria Annunziato, Gioena Pampalone, Marco Pieroni, Mirco Dindo, Francesca Ferlenghi, Gabriele Costantino, Barbara Cellini

**Affiliations:** † Department of Medicine and Surgery, University of Perugia, P.le L. Severi 1, 06132 Perugia, Italy; ‡ Department of Food and Drug, University of Parma, Parco Area delle Scienze 27/A, 43124 Parma, Italy

## Abstract

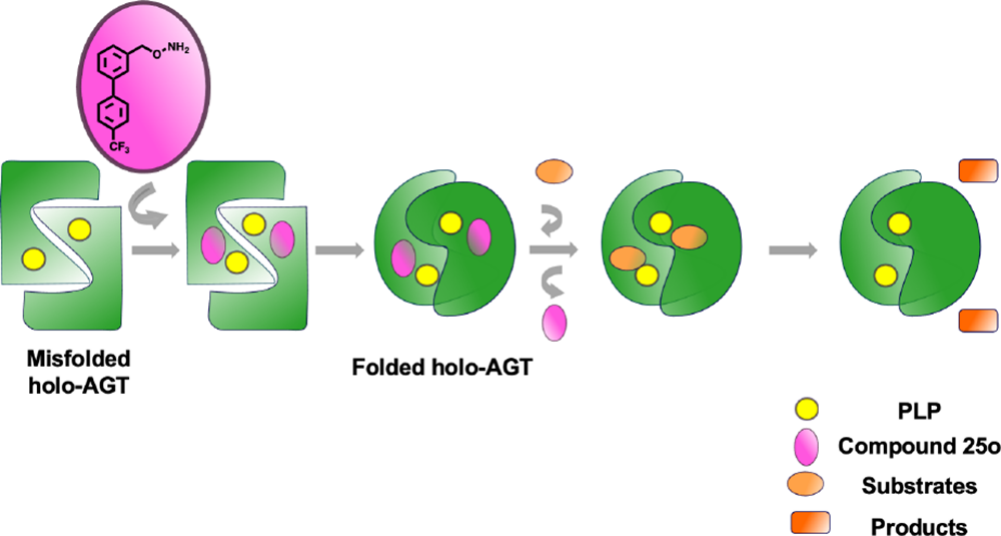

Primary hyperoxaluria type I (PH1) is a rare kidney disease due to the deficit of alanine:glyoxylate aminotransferase (AGT), a pyridoxal-5′-phosphate-dependent enzyme responsible for liver glyoxylate detoxification, which in turn prevents oxalate formation and precipitation as kidney stones. Many PH1-associated missense mutations cause AGT misfolding. Therefore, the use of pharmacological chaperones (PCs), small molecules that promote correct folding, represents a useful therapeutic option. To identify ligands acting as PCs for AGT, we first performed a small screening of commercially available compounds. We tested each molecule by a dual approach aimed at defining the inhibition potency on purified proteins and the chaperone activity in cells expressing a misfolded variant associated with PH1. We then performed a chemical optimization campaign and tested the resulting synthetic molecules using the same approach. Overall, the results allowed us to identify a promising hit compound for AGT and draw conclusions about the requirements for optimal PC activity.

## Introduction

Liver peroxisomal alanine:glyoxylate aminotransferase (AGT) is a homodimeric protein belonging to the fold type I class of pyridoxal 5′-phosphate (PLP)-enzymes.^1^ Each subunit contains one PLP molecule bound to the apoprotein *via* a Schiff base linkage with Lys209 and stabilized by weak interactions at the active site including salt bridges, base stacking, and hydrogen bonds.^1,2^ AGT catalyzes the transamination of l-alanine and glyoxylate to pyruvate and glycine, respectively. Although the catalytic pathway is that typical of PLP-dependent transaminases, the reaction catalyzed by AGT is irreversible under physiological conditions, in agreement with the role of the enzyme in glyoxylate detoxification.^3^ Two polymorphic forms of the *AGXT* gene encoding AGT exist in humans: the *major allele* (encoding AGT-Ma) and the *minor allele* (encoding AGT-Mi). The latter is characterized by a 74-bp duplication in intron 1 and two point mutations causing the P11L and I340M amino acid substitutions.^4^ AGT-Mi exhibits a 30% reduction of transaminase specific activity with respect to AGT-Ma, as well as decreased thermodynamic and kinetic stability.^5−7^ Inherited mutations leading to the loss of AGT functionality determine a rare metabolic recessive disorder known as primary hyperoxaluria type I (PH1, OMIM 259900).^8^ The main hallmark of PH1 is the formation and progressive deposition of calcium oxalate stones in the kidneys, often leading to renal failure and, in the most severe cases, to systemic oxalosis. The clinical aspects of this pathology have been extensively reviewed.^9−11^ PH1 is currently treated by combined or sequential liver–kidney transplantation.^9^ In addition, a substrate-reduction approach based on the silencing of the gene encoding glycolate oxidase has been recently approved in the USA and Europe.^12^ Both transplantation and siRNA therapies are characterized by high costs for the healthcare systems. The administration of pyridoxine (PN), a metabolic precursor of PLP, reduces urinary oxalate excretion by approximately 30% in responsive patients, but they remain susceptible to stone formation.^13,14^ No pharmacological treatments are available for patients unresponsive to PN. Therefore, the need arises to develop cheap and less invasive treatments. Up to now, more than 200 disease-associated mutations of the human *AGXT* gene have been identified. Biochemical and cell biology studies performed in the past few years have highlighted that many mutations cause folding defects in AGT that in turn can determine (i) increased aggregation tendency, either in the cytoplasm or inside peroxisomes; (ii) reduced stability of the dimeric structure of the protein, typically in the apo-form; (iii) enhanced susceptibility to proteolytic degradation; and (iv) mistargeting of the protein to mitochondria.^15−17^ Among the approaches under investigation to treat protein misfolding diseases, one of the most promising involves the use of pharmacological chaperones (PCs). PCs are drug-like molecules able to specifically bind a misfolded protein and improve its folding efficiency, thus recovering the intracellular yield of variants affected by conformational defects.^18,19^ PCs used to treat diseases due to enzymatic deficits are either vitamin derivatives functioning as coenzymes or competitive inhibitors of the disease-causing enzyme.^20−22^ These molecules are usually characterized by a high binding affinity and specificity, two features that allow them to be effective at a very low concentration. In a proof-of-concept study, we showed that an AGT inhibitor acting as PC is aminooxyacetic acid (AOA).^23^ We observed in cellular models that the presence of AOA increases the amount of functional AGT even in cells expressing conformational variants.^23^ In particular, it improves the folding of G41R-Ma, a variant mainly characterized by an increased susceptibility to aggregation and proteolytic degradation, and promotes the peroxisomal localization of G170R-Mi and I244T-Mi, two variants aberrantly targeted to mitochondria.^15^ Since AOA lacks of specificity because it interacts with many PLP-enzymes and with free PLP,^24^ we performed a small-scale screening campaign and identified AOA analogues acting as AGT inhibitors and possibly displaying chaperone activity. Continuing on this path, here we decided to focus our attention on compound **1**, due to its promising preliminary *in vitro* activity, as a feasible starting point for a medicinal chemistry campaign aimed at identifying AGT ligands with improved affinity and chaperone activity. We started from *in silico* screening of commercially available compounds structurally related to compound **1**. Based on the obtained results, we set up a fast and practical synthetic protocol that allowed us to obtain in a few steps 21 derivatives with the desired substituents. We first screened each compound on AGT-Ma and on the G41R-Ma variant, the latter as a prototype of a folding-defective variant, using a twofold strategy: (i) test of binding and inhibition potency on purified proteins and (ii) test of the chaperone activity in a cellular model either in the absence or in the presence of PN. Our results led to the identification of a hit compound showing high affinity for AGT and behaving as PC for folding-defective variants associated with PH1.

## Results and Discussion

### Selection of Commercially Available AGT Ligands

**Figure 1:**
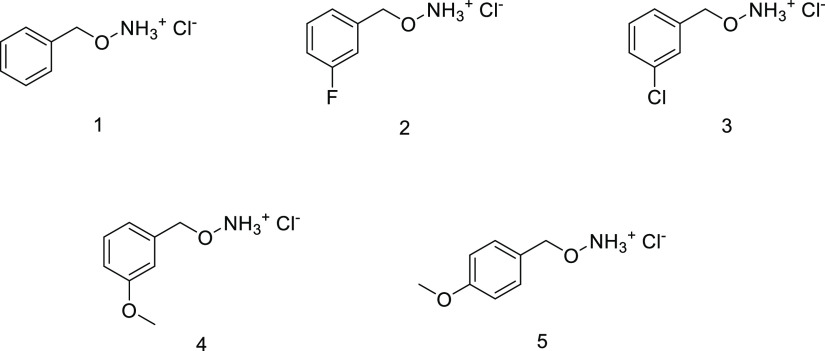
Chemical structures of the selected commercially available analogues of compound 1.

In the search for a specific AGT ligand effective as PC, we started from a known hit compound (compound **1**, Figure 1).^23^ The scaffold of compound **1** was used to launch a small-size screening campaign of commercially available molecules that were tested *in silico* for their putative ability to bind at the AGT active site. Based on their predicted binding affinity for the AGT active site, we selected four analogues of compound **1** (compounds **2**, **3**, **4**, and **5**) (Figure 1).

### Test of Commercially Available Compounds on Wild-Type and Mutant AGT: Binding and Inhibition Potency

**Figure 2:**
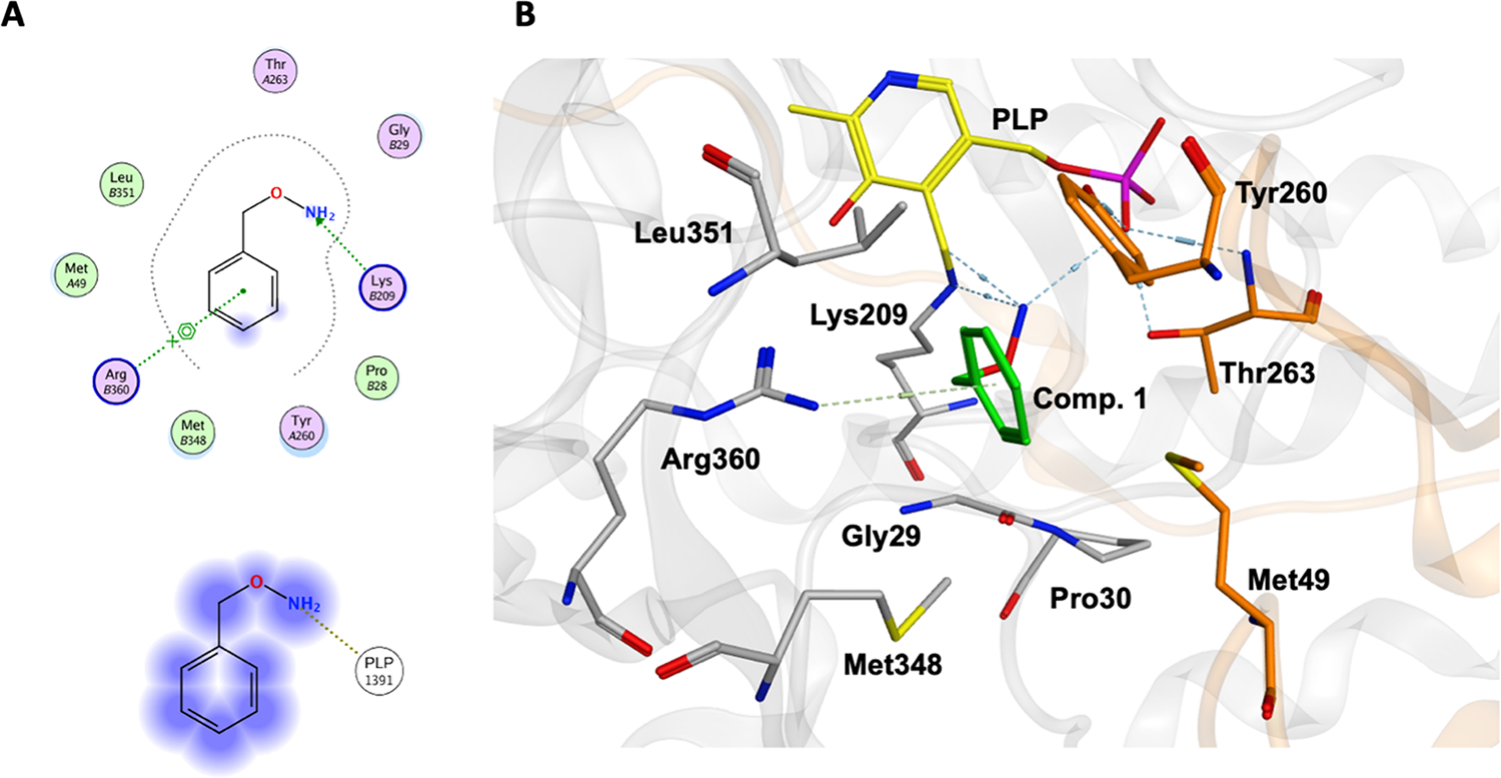
Binding mode of compound **1** into the AGT active site obtained by molecular docking. (A) Two-dimensional (2D) representation showing the interactions of active site residues (upper panel) and PLP (lower panel) with compound **1**. (B) Three-dimensional (3D) representation of the interactions between compound **1** and residues located at the AGT active site. PLP and compound **1** are shown as yellow and green sticks, respectively. Residues belonging to the same subunit of PLP are colored grey, while those belonging to the neighboring subunit are colored orange. The figure was rendered using MOE 2018.0101 (CCG, Montreal, Quebec, Canada) from the crystal structure of the AGT-AOA complex (PDB ID:1H0C).

We evaluated the interaction of compounds **1**, **2**, **3**, **4**, and **5** with AGTwt and the folding-defective G41R variant^25^ by absorbance spectroscopy. As already observed for AOA,^23^ the interaction with each ligand caused the disappearance of the bands at 423 and 340 nm, attributed to the ketoenamine and enolimine tautomers of the internal aldimine, respectively,^3^ and the concomitant appearance of a peak at 374 nm (Figure S1A). Upon excitation of the enzyme–inhibitor complexes at 374 nm, a fluorescence emission spectrum with maximum at ∼450 nm was observed (Figure S1C). Similar spectral changes occurred for the G41R variant, although in the latter case two absorbance bands centered at 370 and at 334 nm were observed in the presence of each ligand (Figure S1B,D) due to subtle active site differences caused by the mutation of Gly41 that affect the tautomeric equilibrium.^25^ Overall, these results indicate that all compounds are able to bind the AGT active site through the formation of an oxime between the carbonyl group of PLP and the amino group of the ligand. We used molecular docking to predict the main interactions driving the formation of a complex between AGT and the compounds under study. The predicted pose of compound **1** is shown in Figure 2, but the results obtained are identical for all tested molecules. First, we found that the aminooxy group is placed in a position comparable to that of AOA,^1^ suitable to interact with PLP generating an external aldimine. The hypothesis that the amino group of compound **1** forms a Schiff base with the carbonyl group of PLP is supported by the following considerations: (i) since peroxisomal matrix proteins like AGT fold in the cell cytosol and are then imported in the fully folded state, they interact with molecules acting as PCs at a pH of approximately 7.2; (ii) considering that the amino group of compound **10b** has a p*K*_a_ of 4.17 ± 0.06 in water, as experimentally measured, it should be in its neutral form at cytosolic pH. *In silico* analyses also revealed that the benzene ring of compound **1** is involved in a cation−π interaction with Arg360, an active site residue that usually forms a salt bridge with the carboxylate group of the substrate.^1^ The latter interaction could strongly influence the binding to the AGT active site and possibly affect the potency of the compounds.

We thus tested the inhibitory activity of each molecule and determined the IC_50_ values, as reported in Table 1. It can be observed that the IC_50_ values for the G41R variant are higher than those for the wild type, in particular in the case of compound **5**. Since the crystal structure of the G41R variant is not available, it is difficult to rationalize these data. Nevertheless, previous analyses have suggested that the mutation of Gly41 could give rise to subtle active site changes through the loop 24-32 connecting the N-terminus to the PLP binding pocket.^25^ More recently, molecular dynamics simulations have shown that helix 48-64 samples alternative conformations inducing a fluctuation that propagates to the active site.^26^ It can be hypothesized that the mutation of Gly41 could affect the conformation of both the loop 24-32 and the helix 48-64, thus accounting for the active site variations. We also observed that the IC_50_ values of the analogues were lower than that of compound **1**, with the only exception being compound **4** on the G41R variant. This suggests that the AGT active site is able to tolerate the presence of substituents of a different nature at the phenyl ring side chain and that they may play a role in improving binding potency.

**Table 1:** IC_50_ Values of Compound **1** and Commercially Available Analogues for AGTwt and the G41R Variant

	wild-type AGT	G41R variant
compound	IC_50_ (μM)	IC_50_ (μM)
**1**	5.6 ± 0.1	12 ± 1
**2**	2.4 ± 0.4	7.5 ± 0.8
**3**	0.7 ± 0.1	2.5 ± 1.8
**4**	1.4 ± 0.2	12 ± 2
**5**	0.16 ± 0.03	5.2 ± 0.3

### Test of Commercially Available Compounds on Wild-Type and Mutant AGT: Inhibition Mechanism

AOA behaves as a slow, tight-binding competitive inhibitor for AGT, i.e., the equilibrium E + I ↔ EI is so shifted toward the EI complex formation that it significantly reduces the population of free inhibitor molecules, and the inhibitor dissociates from the target only upon incubation with the saturating substrate.^23,27,28^ We confirmed that compound **1** and analogues also behave as slow, tight-binding inhibitors, as indicated by the time-dependent recovery of transaminase activity of each AGT-inhibitor complex upon incubation with 200 μM PLP and/or 0.5 M l-alanine at 25 °C.

**Figure 3:**
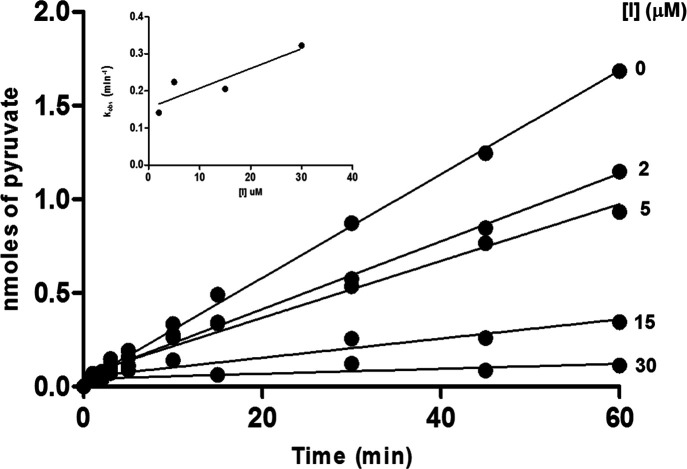
Kinetic characterization of the slow, tight-binding inhibition of AGTwt by compound **3**. (A) Progression curves of pyruvate production during the reaction of AGTwt (0.05 μM) in the presence of l-alanine 250 mM, glyoxylate 10 mM, and compound **3** at the indicated concentrations in KP 0.1 M pH 7.4 at 25 °C for 50 min. Inset: plot of the *k*_obs_ values obtained using eq 1 as a function of inhibitor concentration.

Therefore, we used the progression-curves method^29^ to determine the kinetic parameters of the inhibition. As shown in the example of compound **3** with AGTwt reported in Figure 3, activity decreased with time upon addition of the enzyme to an assay mixture containing the substrate and the inhibitor. While the initial velocity (*v*_0_) was independent of inhibitor concentration, the reaction rate gradually decreased with time, reaching a steady-state value (*v*_s_) inversely proportional to the inhibitor concentration. From the linear fitting of the *k*_obs_ values versus inhibitor concentration, we calculated the association (*k*_1_) and dissociation (*k*_–1_) kinetic rate constants and the inhibition constant (*K*_I_) (Table 2). It can be observed that a remarkable reduction of the *K*_I_ value with respect to compound **1** is only observed when a methoxy substituent is added to the phenylic ring at the *meta*- or *para*-position. The increased affinity of the methoxy-substituted compounds is mainly driven by the increase in k_1_ for AGTwt and by a decrease in k_–1_ for the G41R variant. By analyzing the binding pose of the compounds obtained by molecular docking (Figure 2), we can argue that the effects observed for the derivatives bearing substituents at positions 3 and 4 are mainly linked to their ability to strengthen the cation−π interaction with Arg360 by inductive and resonance effects. Indeed, at position 3, the halogen substituents have an inductive electron-withdrawing effect, thus explaining the reduction of potency (**2** and **3***vs***1**). On the contrary, the shift of the electron-donating methoxy group from position 3 (**4**), where it behaves as an electron-withdrawing group, to position 4 (**5**), where it behaves as an electron-donating group, has the greatest effects on binding potency (**5***vs***1**, **2**, **3**, **4**).^30,31^ It must be mentioned that the docking scores of compounds **1–5** did not show significant differences, as usually observed when considering interactions involving electronic effects, thus preventing the rationalization of the different potency based on *in silico* analyses.

**Table 2:** Inhibition Constant (*K*_I_) and Association (*k*_1_) and Dissociation (*k*_–1_) Kinetic Rate Constants of Compound **1** and Commercially Available Analogues

	wild-type AGT			G41R variant		
compound	*K*_I_ (μM)	*k*_1_ (μM^–1^ min^–1^) × 10^–2^	*k*_–1_ (min^–1^) × 10^–2^	*K*_I_ (μM)	*k*_1_ (μM^–1^ min^–1^) × 10^–2^	*k*_–1_ (min^–1^) × 10^–2^
**1**	0.6 ± 0.3	27 ± 3	17 ± 7	3 ± 1	6 ± 1	19 ± 10
**2**	3.4 ± 1.5	1.8 ± 0.3	6 ± 2	8 ± 2	2.5 ± 0.7	21.0 ± 0.5
**3**	3.8 ± 0.8	0.5 ± 0.2	15 ± 3	2.0 ± 0.4	9 ± 1	21 ± 2
**4**	0.04 ± 0.01	450 ± 4	19 ± 7	0.36 ± 0.08	1.7 ± 0.2	0.6 ± 0.1
**5**	0.08 ± 0.01	120 ± 58	10 ± 3	0.16 ± 0.03	6.0 ± 0.1	0.9 ± 0.2

### Test of Compound **1** and Analogues on Wild-Type and Mutant AGT Expressed in Mammalian Cells

**Figure 4:**
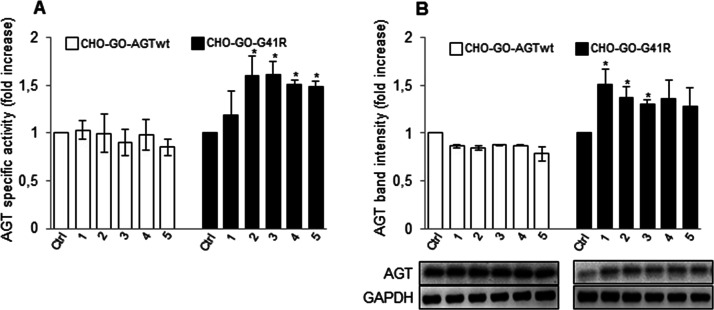
Effect of compound **1** and analogues on AGTwt and the G41R variant expressed in mammalian cells. CHO-GO-AGTwt and CHO-GO-AGT-G41R cells were grown for 7 days in the presence of 50 μM of compound **1** or its analogues. At the end of treatment, cells were detached and lysed, and soluble fraction of each sample was used for (A) transaminase activity determination. The values in CHO-GO-AGTwt control cells (191 ± 10 nmol of pyruvate/min/mg protein) and in CHO-GO-AGT-G41R control cells (6.8 ± 0.7 nmol of pyruvate/min/mg protein) were assumed to be 1 to facilitate the assessment of the changes. Data represent mean ± SEM (*n* = 3). *p < 0.05 vs control cells. (B) AGT protein level quantification by western blot. AGT levels in CHO-GO-AGTwt and CHO-GO-AGT-G41R control cells were assumed to be 1 to facilitate the assessment of the changes. GAPDH was used as loading control. The images are representative of one out of three separate experiments. Data represent mean ± SEM (*n* = 3). *p < 0.05 vs control cells.

The chaperone activity of compound **1** and analogues was studied using a well-known and previously used cellular model of PH1 based on CHO cells stably expressing glycolate oxidase (GO)^32,33^ and wild-type AGT (CHO-GO-AGTwt) or the G41R variant (CHO-GO-G41R). Cells were cultured for 1 week, a time sufficient to allow the majority of AGT to be synthesized in the presence of each putative PC,^34^ in the presence of 50 μM inhibitor, a concentration higher than the *K*_I_ of the analyzed species chosen to take into account any possible effect due to intracellular import. Moreover, we performed the treatment either in a low-B6 medium mimicking a nearly physiological plasmatic vitamin B6 concentration or in the presence of 10 μM PN, mimicking the treatment with vitamin B6. We measured the transaminase specific activity and protein levels in the soluble fraction of the lysate of each sample. We found that neither compound **1** nor its derivatives alter the transaminase specific activity and the protein levels of CHO-GO-AGTwt cells (Figure 4A,B). In agreement with the G41R being a mutation that compromises AGT folding,^25^ the specific activity and expression level of the variant were equal to 10 and 8%, respectively, as compared with AGTwt. Analogs of compound **1** partially rescued the G41R mutation (Figure 4A,B), causing a statistically significant increase in specific activity, along with (for compounds **2** and **3**) an increase in the amount of total protein. This suggests that they could promote the acquisition of the correct three-dimensional structure by misfolded intermediates, thus acting as PCs. However, it must be pointed out that the levels of active protein reached by treated CHO-GO-G41R cells are approximately 4% as compared with those of CHO-GO-AGTwt cells, thus suggesting that only a small rescue of the effects of the mutation occurs in the presence of the compounds.

It has been demonstrated that B6 administration increases the intracellular concentration of PLP, which not only plays a prosthetic role for AGT but also acts as PC by rescuing the effect of conformational mutations that only or mainly affect the folding of the apo-form of the enzyme.^35^ Since PLP acts on apoAGT while the substrate analogues act on the holo-form, we decided to test a combined approach by treating cells with compound **1** and its analogues in the presence of PN. As expected, PN treatment caused a 50% decrease in specific activity of AGTwt due to the inhibition by accumulating intracellular pyridoxine 5′-phosphate (PNP), as previously reported.^15^ On the other hand, it caused a more than 10-fold increase in G41R-Ma specific activity (from 6.8 ± 0.7 to 114 ± 19 nmol of pyruvate/min/mg protein) and 1.5-fold increase in protein levels. These data strongly support the idea that the G41R mutation is responsive to vitamin B6, in line with published data on a patient expressing the variant.^36^ Upon combined treatment with PN and each compound under study, we did not observe changes in either the protein level or specific activity of AGTwt. In the presence of PN, none of the ligands modified the specific activity of G41R, although protein levels were increased and reached statistical significance in the presence of PN and compound **3** (Figure S2A,B). Although it is not easy to interpret these data, it can be speculated that any possible additive or synergic effect of the combined treatment is masked by the known inhibition of activity by PNP.^15^ Nonetheless, the data confirm the effectiveness of compound **3** as PC.

Altogether, the paired analysis of the data at the protein and cellular level highlights that compounds showing the highest affinity for AGT do not display the best chaperone activity in a cellular system. Although we cannot exclude that the different behavior can depend on a different ability to cross the plasma membrane, it should be also considered that the kinetics of binding and dissociation from the target could be very important in determining the ability of a molecule as PC. The observation that compounds **2** and **3** show the lowest differences between association and dissociation rates allows us to speculate that the ability to rapidly establish an equilibrium during interaction with the target could be an important parameter governing chaperone activity.

### Chemistry

**Figure 5:**
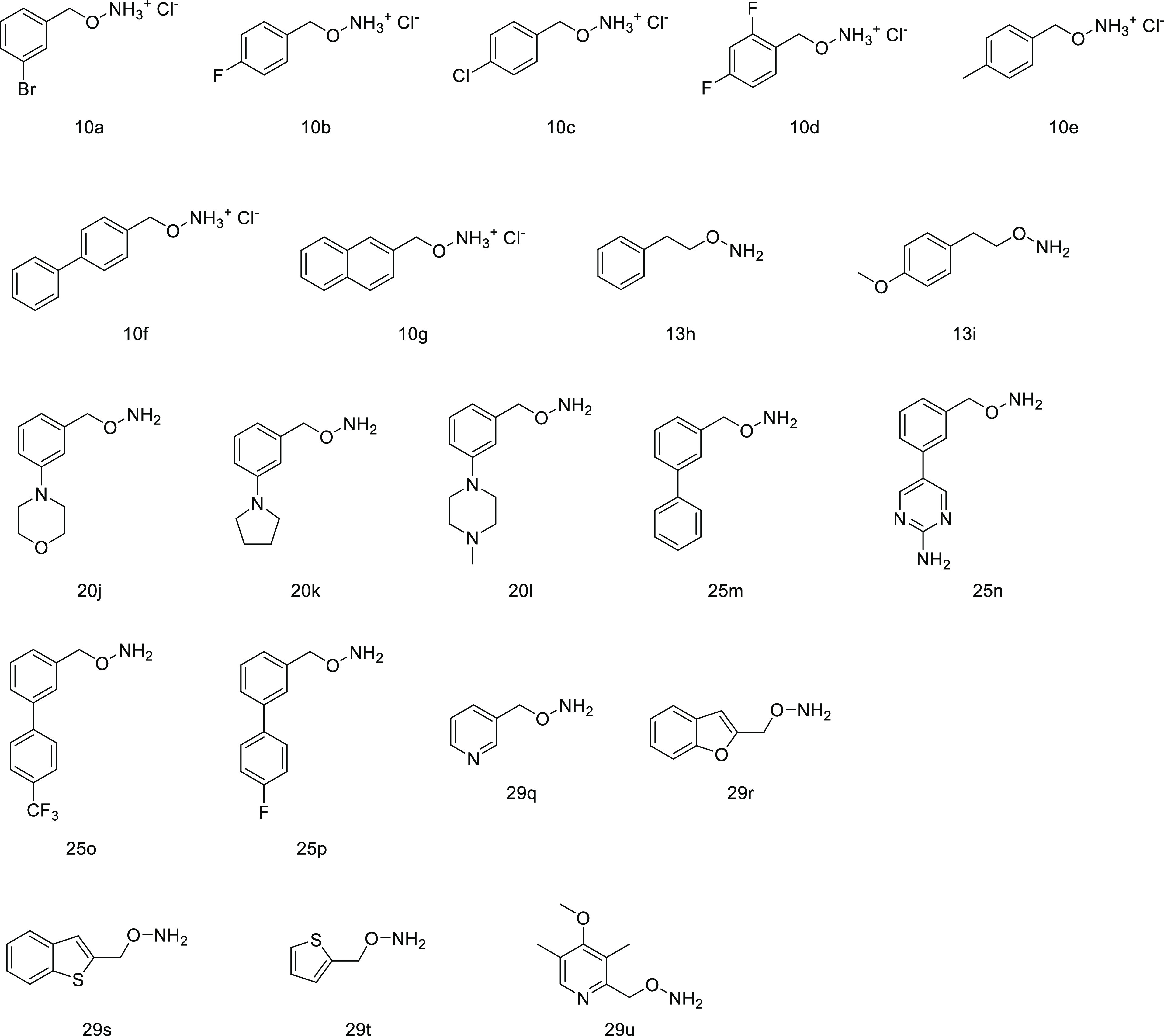
Chemical structures of synthesized AGT ligands.

We started a medicinal chemistry campaign to improve the chaperone activity of AGT ligands. Starting from compound **1** as the scaffold, we synthesized 21 compounds (Figure 5) taking advantage of the results obtained by the initial docking studies and by *in vitro* and in-cell analyses. The selection of compounds to be synthesized was based on the following key aspects: (i) definition of the ability of the enzyme active site to host substituents of different sizes and natures; (ii) introduction of heteroaromatic and heteroaliphatic substituents to change the electron density charge of the aromatic ring interacting with Arg360; (iii) chemical feasibility; and (iv) selection of the most promising compounds able to preserve the nature of the interaction observed by means of docking studies.

**Scheme 1:**
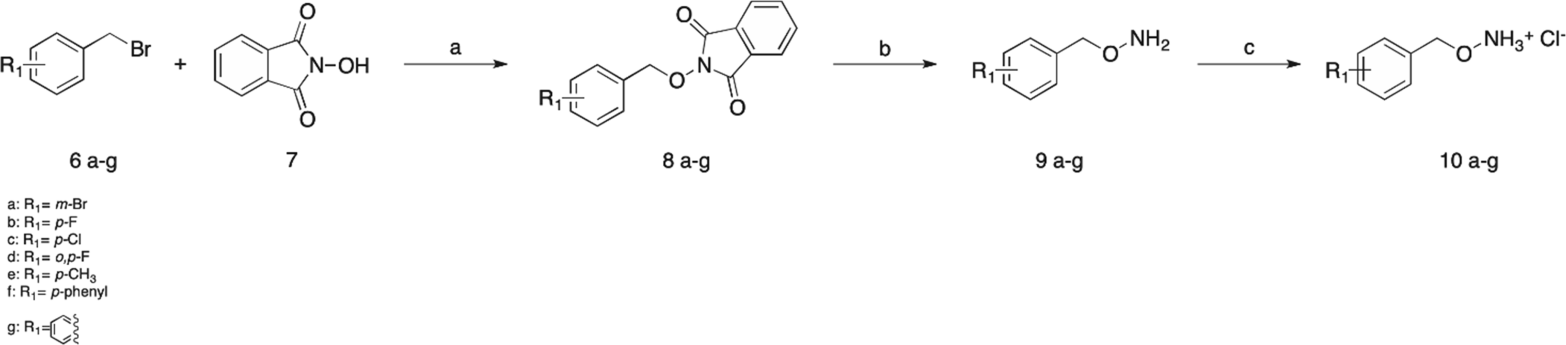
Synthesis of Compounds **10a–g** Reagents and conditions: (a) K_2_CO_3_, DMSO, r.t., 18 h, 94–96%; (b) hydrazine, DCM, r.t., 2 h, 92–97%; (c) HCl 4 N in dioxane, Et_2_O (2:1) 92–98%.

The synthesis of final compounds **10a**–**g** started from the benzyl bromide **6a**–**g** properly substituted that reacted with *N*-hydroxyphtalimide (**7**) in the presence of K_2_CO_3_ in DMSO to give the intermediates **8a**–**g**^37^ (Scheme 1). The deprotection of the amino group was performed with hydrazine, and finally, the salification of the amino group was done with HCl.

**Scheme 2:**
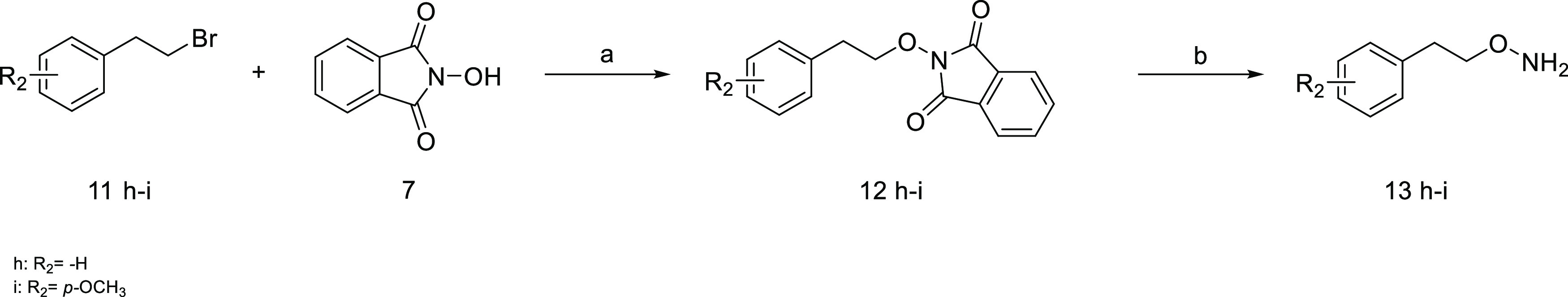
Synthesis of Compounds **13h–i** Reagents and conditions: (a) K_2_CO_3_, DMSO, r.t., 18 h, 82–91%; (b) hydrazine, DCM, r.t., 2 h, 83–87%.

For the synthesis of the final compounds **13h**–**l**, where the methylene chain was lengthened and changed with an ethylene chain, a protocol similar to that reported above was followed, which allowed us to obtain the desired compounds in a few steps and good yields **(**Scheme 2**)**.

**Scheme 3:**
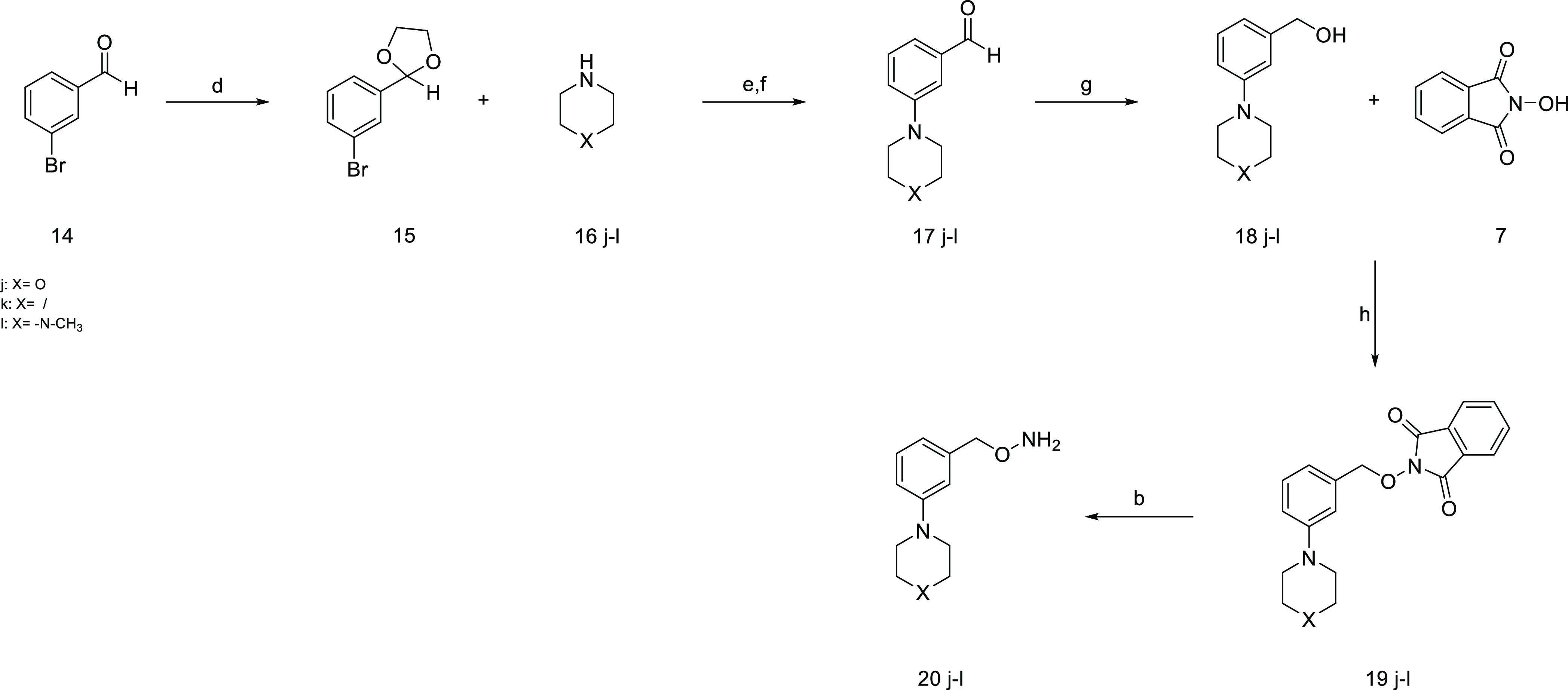
Synthesis of Compounds **20j-l** Reagents and conditions: (d) propane 1,3-diol, *p-*TsOH, toluene dry, 110 °C, 18 h, 92%; (e) Pd_2_(dba)_3_, BINAP, NaO*t*Bu, toluene dry, 110 °C, 18 h, 56–68%; (f) HCl 1 N, 0 °C, 2 h, 73–84%; (g) LiAlH_4_, THF dry, 0 °C, 30 min, 43–78%; (h) PPh_3_, DEAD, THF dry, r.t., 18 h, 67–85%; (b) hydrazine, DCM, r.t., 2 h, 85–92%.

The synthesis of the final compounds **20j–l** started with the protection of the aldehyde group of compound **14** with 1,3-propanediol in the presence of para-toluene sulfonic acid in toluene at 110 °C for 18 h. Then a Pd catalyzed Buckwald–Hartwig amination with the properly substituted amine **16j–l**(38) and the consequent deprotection of the aldehyde group in acidic conditions led to compounds **17j–l**. Afterward, a reduction was performed to convert the aldehyde in primary alcohol. The intermediates **18j–l** reacted with *N*-hydroxyphthalimide to obtain intermediates **19j–l** through a Mitsunobu reaction.^39^ Finally, the amino group was deprotected with hydrazine, and the final compounds **20j–l** were obtained **(**Scheme 3**)**.

**Scheme 4:**
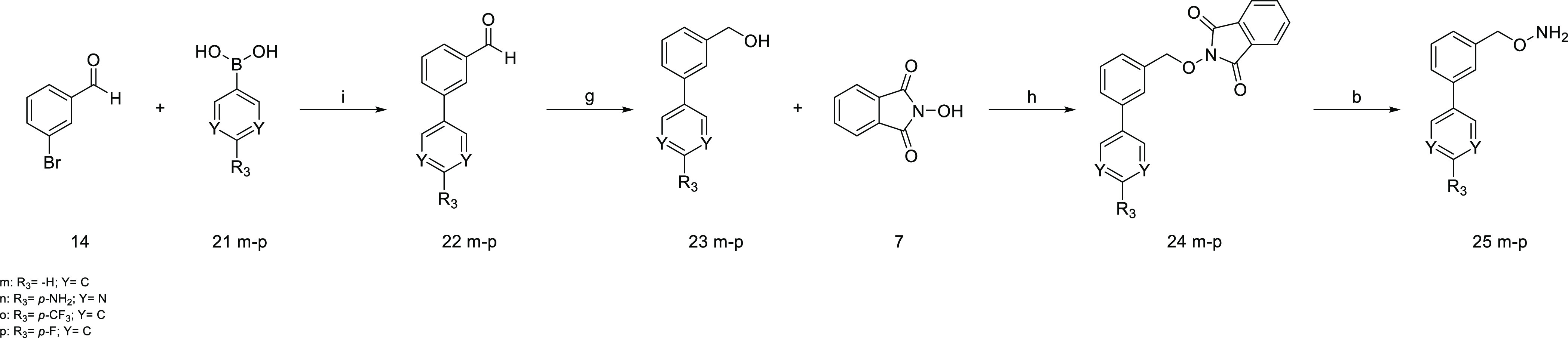
Synthesis of Compounds **25m–p** Reagents and conditions. (i) Pd(PPh_3_)_4_, Cs_2_CO_3_, DME/H_2_O (2:1), mw, 140 °C, 30 min, 100 W, 67–78%; (g) LiAlH_4_, THF dry, 0 °C, 30 min, 56–82%; (h) PPh_3_, DEAD, THF dry, r.t., 18 h, 72–88%; (b) hydrazine, DCM, r.t., 2 h, 92–96%.

The synthesis of compounds **25m-p** started from compound **14**, and an already optimized protocol was followed, according to which **14** reacted with the appropriate boronic acid **21m-p** and, through a Suzuki–Miyaura reaction, the aldehydes **22m**–**p** properly substituted in meta position were obtained.^40^ In the following step, the aldehyde group was reduced to a primary alcohol with LiAlH_4_, and the product of this reaction was allowed to react with *N*-hydroxyphthalimide through a Mitsunobu reaction to obtain the intermediates **24m**–**p**. Finally, the amino group was deprotected in the presence of hydrazine, and the desired final compounds (**25m**–**p**) were obtained (Scheme 4).

**Scheme 5:**
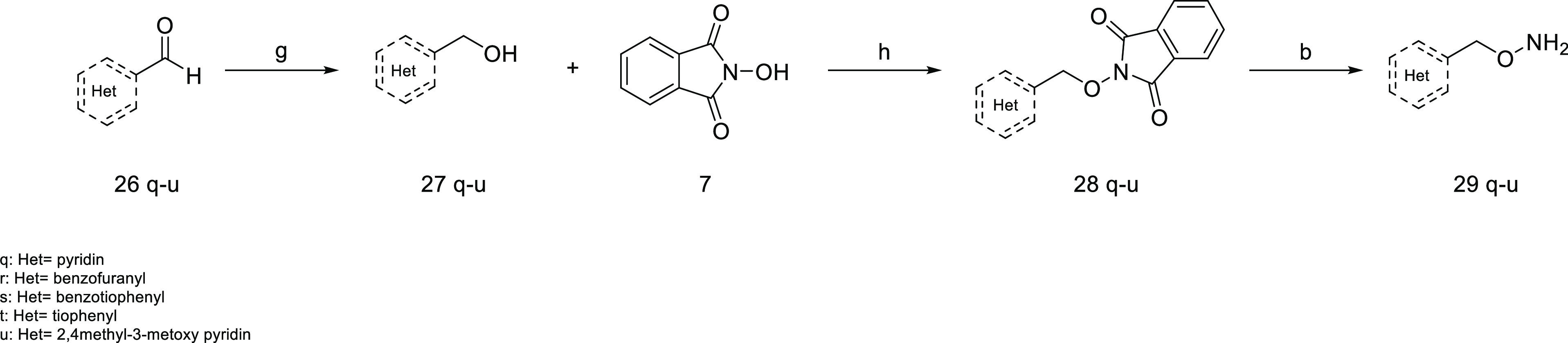
Synthesis of Compounds **29q–u** Reagents and conditions. (g) LiAlH_4_, THF dry, 0 °C, 30 min, 74–80%; (h) PPh_3_, DEAD, THF dry, r.t., 18 h, 65–73%; (b) hydrazine, DCM, r.t., 2 h, 90–94%.

The synthesis of the final compounds **29q**–**u** started from the appropriate heterocycle **26q**–**u** carrying an aldehyde group that was reduced to primary alcohol (**27q–u**). These intermediates reacted with *N*-hydroxyphthalimide through a Mitsunobu reaction to obtain the compounds (**28q**–**u**), and finally, the amino group was deprotected with hydrazine to obtain the desired final compounds **29q**–**u** (Scheme 5).

### Test of Synthesized AGT Ligands on Purified Wild-Type and Mutant AGT

We tested the synthesized AGT ligands using the same experimental approach described above and aimed at defining (a) the binding of each compound at the active site, (b) the kinetic parameters of the inhibition, and (c) the PC activity on AGT expressed in mammalian cells. All synthesized compounds except **13h**, **13i**, **29q**, and **29t** induced spectral changes indicating their binding at the active site of both AGTwt and the G41R variant generating the oxime intermediate (Figure S3). No signal of binding was observed in the case of **13h** and **13i**, while a very slow binding rate was observed for compounds **29q** and **29t** (data not shown). The observed IC_50_ values in the low micromolar or nanomolar range suggest that chemical optimization has improved binding potency (Table 3). Notably, we could only establish an upper limit for the IC_50_ value of compounds **29r**, **10f**, **25m**, **25p**, **29s**, and **29u** for the variant because the experimental conditions did not allow further reduction of the concentration of the inhibitor.

**Table 3:** IC_50_ Values of Synthesized Compounds on Wild-Type AGT and G41R Variant in Purified Forms

	wild-type AGT	G41R variant
compound	IC_50_ (μM)	IC_50_ (μM)
**10a**	0.12 ± 0.02	0.40 ± 0.07
**10b**	0.3 ± 0.1	1.4 ± 0.3
**10c**	0.06 ± 0.01	0.9 ± 0.1
**10d**	0.3 ± 0.1	1.8 ± 0.1
**10e**	0.16 ± 0.04	1.1 ± 0.1
**10f**	0.20 ± 0.01	<0.2
**10g**	0.040 ± 0.006	0.10 ± 0.02
**20j**	0.85 ± 0.07	20 ± 5
**20k**	1.8 ± 0.1	1.7 ± 0.7
**20l**	4.6 ± 0.5	11 ± 2
**25m**	0.23 ± 0.03	<0.2
**25o**	0.55 ± 0.03	0.37 ± 0.03
**25p**	0.28 ± 0.02	<0.2
**29r**	0.23 ± 0.01	<0.2
**29s**	0.35 ± 0.02	<0.2
**29u**	0.62 ± 0.08	<0.2

We then determined the kinetic parameters of the inhibition (Table 4). Many compounds displayed inhibition constants in the nanomolar range, thus further confirming that the rational optimization has been successful in improving the inhibition potency. In line with our previous hypothesis, compounds showing the highest binding potency are those with substituents able to strengthen the cation−π interaction between Arg360 and the aromatic ring (**10g**, **25m**, **10a**, **10b**, **10c**). The observed trend, supporting a pivotal role of electronic interaction with Arg360, is also highlighted by the observation that (*i*) compounds bearing a longer spacer for the aminooxy group are inactive (**13h** and **13i**) likely because they do not properly accommodate in the enzyme active site, thus hampering the formation of the cation−π interaction with Arg360, and (*ii*) the substitution of the phenyl ring with heteroaromatic rings (**29q** and **29t**) has detrimental effects on binding potency, and this behavior is reversed when the heteroaromatic ring is substituted with electron-rich groups (**29s** and **29u**). It can be observed that most of the compounds active on wild-type AGT are also active on the G14R variant. The slight differences observed between the two forms can be ascribed to subtle active site changes that the mutation causes through the loop 24-32 and helix 48-62 connected to the PLP binding pocket, as previously mentioned.^25,26^

**Table 4:** Inhibition Constant (*K*_I_) and Association (*k*_1_) and Dissociation (*k*_–1_) Kinetic Rate Constants of Synthesized Compounds^a^

	wild-type AGT			G41R variant		
compound	*K*_I_ (μM)	*k*_1_ (μM^–1^ min^–1^) × 10^–2^	*k*_–1_ (min^–1^)	*K*_I_ (μM)	*k*_1_ (μM^–1^ min^–1^) × 10^–2^	*k*_–1_ (min^–1^)
**10a**	0.05 ± 0.01	0.060 ± 0.007	0.030 ± 0.005	1.2 ± 0.1	0.07 ± 0.03	∼0.08
**10b**	0.086 ± 0.007	10.5 ± 0.5	0.01 ± 0.004	0.13 ± 0.03	1.1 ± 0.1	∼0.001
**10c**	3.78 ± 0.02	0.14 ± 0.01	0.07 ± 0.01	∼0.03	0.22 ± 0.04	∼0.007
**10d**	17 ± 0.03	0.010 ± 0.006	0.13 ± 0.03	15.3 ± 0.1	0.010 ± 0.003	0.12 ± 0.06
**10e**	0.40 ± 0.03	0.17 ± 0.01	0.07 ± 0.03	2 ± 0.2	0.13 ± 0.05	∼0.2
**10f**	2 ± 0.006	2.6 ± 0.2	0.050 ± 0.005	1.20 ± 0.01	1.9 ± 0.3	∼0.02
**10g**	0.0020 ± 0.0003	15.5 ± 4	∼0.0003	0.99 ± 0.0002	0.90 ± 0.02	0.0090 ± 0.0003
**20j**	n.a.			0.60 ± 0.01	0.22 ± 0.02	∼0.001
**20k**	0.24 ± 0.02	0.80 ± 0.01	∼0.002	5.70 ± 0.03	1.3 ± 0.2	0.08 ± 0.03
**20l**	110 ± 0.01	0.095 ± 0.013	0.10 ± 0.01	n.a.		
**25m**	0.027 ± 0.003	14.5 ± 2.5	∼0.004	<0.015	24 ± 2	<0.003
**25o**	0.72 ± 0.01	3.6 ± 0.3	0.02 ± 0.01	1.40 ± 0.02	1.8 ± 0.3	∼0.02
**25p**	3.50 ± 0.01	2.6 ± 0.2	0.10 ± 0.01	0.24 ± 0.02	7.2 ± 0.6	∼0.02
**29r**	2 ± 0.005	4.1 ± 0.2	0.080 ± 0.004	1.40 ± 0.04	4 ± 1	∼0.06
**29s**	0.11 ± 0.02	9.8 ± 0.4	∼0.01	0.80 ± 0.02	8.6 ± 0.6	0.07 ± 0.02
**29u**	4.60 ± 0.01	0.80 ± 0.05	0.04 ± 0.01	<0.015	2.30 ± 0.06	<0.0003

a n.a., not available.

### Test of Synthesized AGT Ligands on Wild-Type and Mutant AGT Expressed in Mammalian Cells

**Figure 6:**
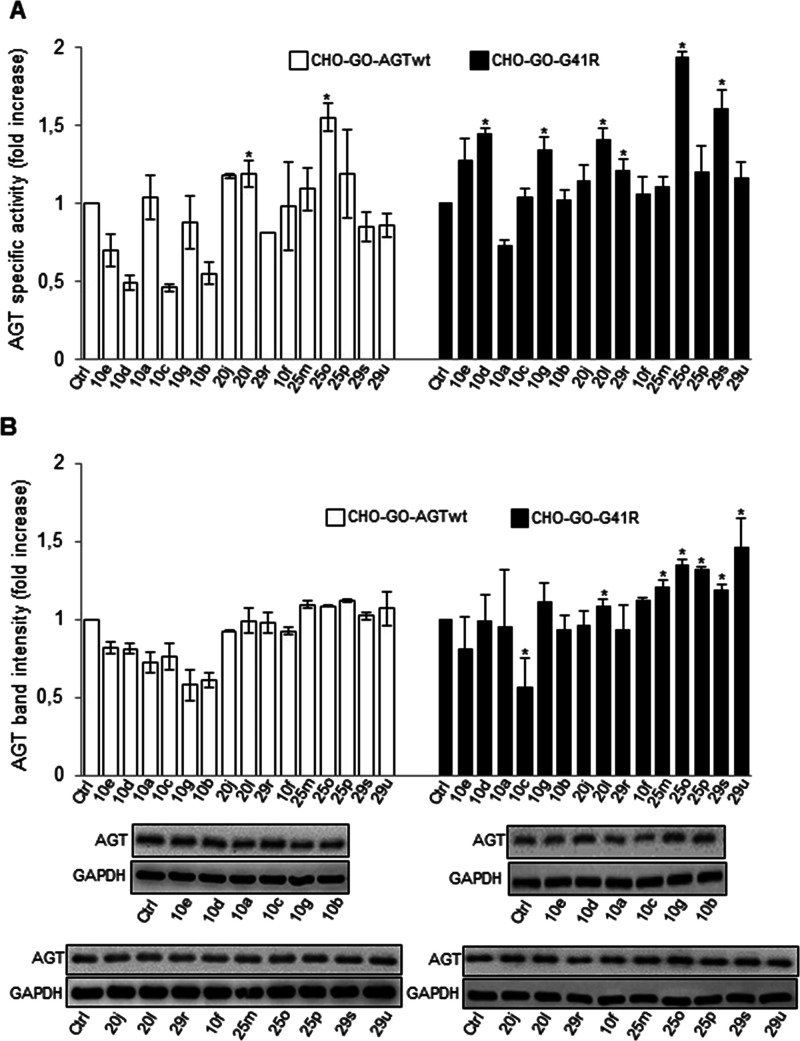
Effects of synthesized compound treatment on AGTwt and the G41R variant expressed in mammalian cells. CHO-GO-AGTwt and CHO-GO-AGT-G41R cells were grown for 7 days in the presence of 50 μM of each compound, as indicated. At the end of treatment, cells were detached and lysed, and the soluble fraction of the lysate was used for (A) transaminase activity determination. The specific activity of AGT in CHO-GO-AGTwt control cells (191 ± 10 nmol of pyruvate/min/mg protein) and in CHO-GO-AGT-G41R control cells (6.8 ± 0.7 nmol of pyruvate/min/mg protein) was assumed to be 1 to help assess the changes. Data represent mean ± SEM (*n* = 7). *p < 0.05 vs control cells. (B) AGT protein level quantification by western blot. AGT levels in CHO-GO-AGTwt and CHO-GO-AGT-G41R control cells were assumed to be 1 to help assess the changes. GAPDH has been used as the loading control. The images are representative of one out of three separate experiments. Data represent mean ± SEM (*n* = 4). *p < 0.05 vs control cells.

We tested the effect of synthesized compounds on AGT expression and specific activity in CHO-GO-AGTwt and CHO-GO-G41R cells (Figure 6A,B). We did not observe any increase in specific activity of AGTwt and G41R upon culturing cells in the presence of compounds **10e**, **10a**, **10c**, **10g**, **10b**, **20j**, **10f**, **25m**, or **25p**, although there was an increase in protein levels in the western blot in the case of compounds **25m** and **25p**. On the other hand, the specific activity of the G41R variant showed a significant increase upon culturing cells in the presence of compounds **10d**, **20l**, **29r**, **25o**, **29s**, and **29u**. These changes were not always associated with comparable increased protein levels in the soluble fraction of the lysate. Nevertheless, it must be mentioned that western blot analyses measure protein levels under denaturing conditions, thus implying that they do not allow one to distinguish between folded and unfolded AGT. Thus, it can be hypothesized that compounds **10d**, **20l**, **29r**, **25o**, **29s**, and **29u** shift the equilibrium toward the native state without affecting the global synthesis and/or degradation rates of AGT. Notably, the treatment with compounds **10e**, **10d**, **10c**, **10b**, and **29r** led to a decrease in AGTwt specific activity that was not paired by an equivalent reduction in protein levels. This effect is also observed with the variant in the case of compound **10a**. The most probable explanation is that compounds **10e**, **10d**, **10c**, **10b**, and **29r** bind so tightly at the active site that they do not dissociate from the enzyme upon cell lysis and dilution in the reaction mixture, thus resulting in apparent inhibition.

In combination with PN, compounds **10e**, **10d**, **10c**, and **10b** did not change AGTwt activity but reduced the one of the G41R variant due to an apparent inhibition, as shown by the fact that the reduced activity is compounded by reduced protein levels only in the case of compound **10d**. Compounds **10a**, **29r**, **10f**, **25m**, **25p**, **25o**, **29s**, and **29u** cause an increase of AGTwt activity that is accompanied by increased protein levels in the case of **10f**, **25o**, and **29u**. Compounds **10a**, **29r**, and **10f** have no effect on the variant, while compounds **25 m**, **25p**, **29s**, and **29u** increase the variant protein levels without affecting specific activity. This would suggest that the compounds could play a chaperone role but remain at least partly bound at the active site, thus causing inhibition. Finally, only compounds **20l** and **25o** increased the specific activity of the G41R variant, an effect accompanied by an increase in protein levels in the case of **25o**.

To select the best hit among the tested molecules, we performed a ranking of the compounds based on their effect on specific activity and expression levels of AGTwt and the G41R variant. Based on this analysis, we identified five compounds as the best hits for each form of the enzyme (Table S1). We then evaluated the viability of both CHO-GO-AGTwt and CHO-GO-G41R cells upon treatment with each inhibitor in the absence or presence of PN to have insights into any toxic effect (Figure S5). The viability of CHO-GO-AGTwt did not change in the presence of compounds **10d**, **25o**, **29s**, and **29u**, while it was reduced in the presence of compound **20l**. On the other hand, the viability of CHO-GO-G41R cells increased in the presence of compound **10d** and compounds **29u** and **25o** without PN or in the presence of compounds **25o** and **29u** and PN, while it slightly decreased in the presence of compound **20l** with or without PN.

Overall, by pairing the data obtained on AGT expression and activity with those on cell viability, we could observe that (i) the five selected compounds (**10d**, **20l**, **25o**, **29s**, **29u**) increase the specific activity of G41R, a model of a misfolded variant, although with different efficiency; (ii) compounds **20l** and **25o** lead to improved activity and to improved or unaltered expression in the absence and in the presence of PN in both AGTwt and G41R variant, and they do not affect cell viability. Since the effects of compound **25o** are higher than those of compound **20l**, we chose compound **25o** as the best hit for subsequent studies.

In line with our previous observations, compounds showing the best chaperone activity in the cellular system are those showing an intermediate affinity for AGT, and also in this case, compounds with the smallest differences between association and dissociation rate constants seem to be the most promising (**25o**, **20l**, and **29u**).

### Chaperone Effect of Compound **25o** on Common AGT Variants Associated with PH1

**Figure 7:**
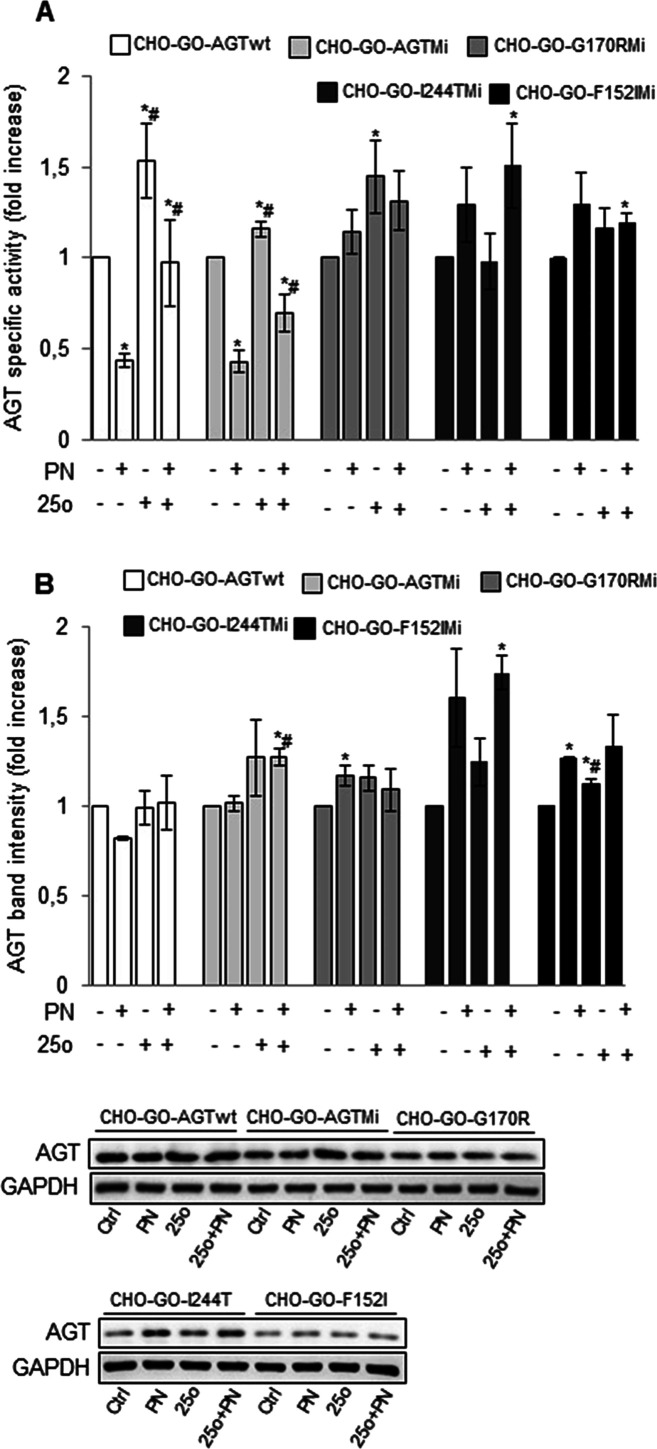
Action of compound **25o** as pharmacological chaperone for common pathogenic AGT variants. CHO-GO-AGTwt, CHO-GO-AGT-Mi, CHO-GO-G170R-Mi, CHO-GO-I244T-Mi, and CHO-GO-F152I-Mi cells were grown for 7 days in the absence or presence of 50 μM **25o** and 10 μM PN. At the end of treatment, cells were detached and lysed, and the soluble fraction of each sample used for (A) AGT enzymatic activity determination. The AGT specific activity of control cells of each clone was assumed to be 1 to help assess the changes (CHO-GO-AGTwt cells: 191 ± 10 nmol of pyruvate/min/mg protein; CHO-GO-AGT-Mi: 132 ± 9 nmol of pyruvate/min/mg protein; CHO-GO-G170R-Mi: 54 ± 7 nmol of pyruvate/min/mg protein; CHO-GO-I244T-Mi: 28 ± 5 nmol of pyruvate/min/mg protein; CHO-GO-F152I-Mi 24 ± 5 nmol of pyruvate/min/mg protein). Data represent mean ± SEM (*n* = 3). *p < 0.05 vs respective control cells. ^#^*p* < 0.05 vs respective PN-treated cells. (B) AGT expression level quantification. AGT levels in control cells of each clone were assumed to be 1 to help assess the changes. GAPDH has been used as loading control. The images are representative of one out of three separate experiments. Data represent mean ± SEM (*n* = 3). *p < 0.05 vs respective control cells. ^#^*p* < 0.05 vs respective PN-treated cells.

Over 200 mutations have been identified in the *AGXT* gene, some of the most common missense being the G170R and I244T, and F152I replacements. All these mutations are pathogenic only on the background of the minor allele^5,41−44^ Moreover, patients bearing the G170R or the F152I mutation on the minor allele are responsive to vitamin B6.^14^ We evaluated whether compound **25o** was able to modulate the specific activity and expression level of AGT of CHO-GO-AGT-Mi cells, which exhibit a reduction of 30% of specific activity with respect to CHO-GO-AGTwt, and of cells expressing the pathogenic forms: CHO-GO-G170R-Mi, CHO-GO-I244T-Mi, and CHO-GO-F152I-Mi, in the absence or presence of PN (Figure 7).

We confirmed that PN treatment results in the inhibition of AGTwt and AGT-Mi activity, while it causes an increase of protein levels and/or specific activity of the pathogenic variants.^45^ In addition, we found that compound **25o** caused (i) an increase in the specific activity and expression levels of AGT-Mi both in the absence and in the presence of PN. The latter effect is analogous to that observed in the case of AGTwt except for a more marked increase in protein levels that is probably due to the enhanced tendency to degradation caused by the P11L polymorphic mutation typical of the minor allele^46,47^ and (ii) an increase in the specific activity of G170R-Mi in the absence of PN. On the one hand, since the G170R-Mi variant is usually responsive to PN both *in vitro* and in patients,^46−48^ it could be argued that compound **25o** is not able to induce additive effects on a PN-treated background. However, it cannot be excluded that, under *in vivo* conditions, the different metabolism of PN and compound **25o** could give to patients expressing the G170R-Mi variant a significant benefit from a combined treatment. Compound **25o** also caused (iii) an increase in the specific activity and protein levels of I244T-Mi in the presence of PN. Data obtained in mammalian cells indicate that the I244T-Mi variant is highly prone to aggregation but is mainly present as holoenzyme.^46^ Thus, compound **25o** could exert its PC action on the holo-form by increasing protein solubility. Lastly, it also caused (iv) a small but statistically significant increase of F152I-Mi specific activity in co-treatment with PN, accompanied by increased protein levels also in the presence of the compound alone. The F152I-Mi variant shows very low expression levels, which can be rescued only by the concomitant presence of the cofactor and compound **25o**, which can possibly cooperate to promote the achievement of the native structure. Notwithstanding the different responsiveness of the variants, our data suggest that compound **25o** might represent a PC for common pathogenic forms of AGT displaying conformational defects. Nevertheless, it must be taken into account that the treatment of cells expressing PH1-associated variants does not allow them to reach the same protein levels and specific activity of cells expressing the nonpathogenic forms AGT-Ma and AGT-Mi, thus implying that further compound optimization will be necessary.

## Conclusions

In this work, we used ligand-based drug discovery approaches based on *in silico* experiments to select and synthesize putative candidates as PCs for AGT. We identified a novel hit compound (**25o**) able to bind the enzyme with high affinity and stabilize the protein in a cellular environment. Our data suggest that compound **25o** could counteract conformational defects caused by some of the most common mutations associated with PH1. In this regard, although promising treatment options for PH1 based on the RNA interference technology have been recently approved by both the FDA and EMA and others are currently tested at the clinical level,^49,50^ the availability of treatment strategies based on small-molecule drugs is an unmet need. They could represent a noninvasive and cheap preemptive approach for patients affected by milder forms of the disease, as well as the only option for patients who have no access to biological drugs.

Besides their significance in the possible future development of a pharmacological therapy for PH1, our results also give interesting insights into the possible correlation between the kinetic parameters of the ligand–protein interaction and the PC activity in a biological environment of a cell. Since classical PC targeting enzymes are designed as competitive inhibitors to exploit the active site and increase binding specificity,^51^ a suitable balance between the inhibitor and chaperone activity should be achieved to maximize the efficiency as PC.^52^ Our data suggest that the best candidates are not those endowed with the highest affinity for the target but rather those showing higher values of kinetic association and dissociation constants. This could be due to the possibility to establish a rapid equilibrium, which on the one hand can promote the chaperone effect by preventing accumulation of partly folded intermediates, while on the other can allow the dissociation of the inhibitor once the native structure is achieved in the presence of the physiological substrate. Although the predictive character of the data must be taken with care, because possible differences of compound permeability and folding kinetic aspects of the mutants are not considered, the combination of molecular and cellular data on putative PCs could be very useful to guide the drug discovery process, paving the way for further medicinal chemistry optimization strategies.

## Experimental Section

### Chemistry

All the reagents were purchased from Sigma-Aldrich, Alfa-Aesar, and Enamine at reagent purity and, unless otherwise noted, were used without any further purification.

Dry solvents used in the reactions were obtained by distillation of technical-grade materials over appropriate dehydrating agents. Microwave reactions were performed using the CEM microwave synthesizer Discover model. Reactions were monitored by thin layer chromatography on silica gel coated aluminum foils (silica gel on Al foils, SUPELCO Analytical, Sigma-Aldrich) at both 254 and 365 nm wavelengths. When indicated, intermediates and final products were purified through silica gel flash chromatography (silica gel, 0.040–0.063 mm) using appropriate solvent mixtures. ^1^H NMR and ^13^C NMR spectra were recorded on a BRUKER AVANCE spectrometer at 300, 400, and 100 MHz, respectively, with TMS as internal standard. ^1^H NMR spectra are reported in this order: multiplicity and number of protons. Standard abbreviations indicating the multiplicity were used as follows: s = singlet, d = doublet, dd = doublet of doublets, t = triplet, q = quadruplet, m = multiplet and br = broad signal. HPLC/MS experiments were performed with HPLC instrumentation (Agilent 1100 series, equipped with a Waters Symmetry C18, 3.5 μm, 4.6 × 75 mm column) and MS instrumentation (Applied Biosystem/MDS SCIEX, with API 150EX ion source). An Accela UHPLC system (Thermo, USA) equipped with a Waters XSelect HSS T3 column (100 × 2.1 mm i.d., 3.5 μm particle size; Waters, USA) was used for chromatographic separation. A Thermo TSQ Quantum Access Max triple quadrupole mass spectrometer (Thermo, San Jose, CA, USA) equipped with a heated electrospray ionization (H-ESI) source was employed for compound detection (see SI Annex 1). HRMS experiments were performed with an LTQ ORBITRAP XL THERMO.

All compounds were tested as 95% purity samples or higher (by HPLC/MS).

### General Procedure for the Synthesis of Compounds **8a**–**g** and **12h**–**i**

The appropriate benzyl bromide **6a**–**g** (2 equiv) and anhydrous potassium carbonate (0.8 equiv) were added to a solution of *N*-hydroxyphtalimide (1 equiv) in DMSO (1.5 mL/mmol). The resulting mixture was stirred for 18 h at room temperature and then was checked by TLC. Then 3 mL/mmol of cold water was added, and the resulting mixture was stirred for 30 min. The obtained white precipitate was filtered and washed with water (×3) to give the products as white powder. Yields: 82–96%.

#### 2-((3-Bromobenzyl)oxy)isoindoline-1,3-dione (**8a**)

^1^H NMR (300 MHz, CDCl_3_) δ 7.87–7.71 (m, 5H); 7.54–7.50 (m, 2H); 7.31–7.26 (m, 1H); 5.19 (s, 2H).

LC–MS calcd for C_15_H_10_BrNO_3_ ([M + H]^+^) 330.98; found 333.14.

#### 2-((4-Fluorobenzyl)oxy)isoindoline-1,3-dione (**8b**)

^1^H NMR (300 MHz, CDCl_3_) δ 7.92–7.81 (m, 4H); 7.08 (t, *J* = 9, 4H); 5.20 (s, 2H).

LC–MS calcd for C_15_H_10_FNO_3_ ([M + H]^+^) 272.06; found 272.24.

#### 2-((4-Chlorobenzyl)oxy)isoindoline-1,3-dione (**8c**)

^1^H NMR (300 MHz, CDCl_3_) δ 7.86–7.75 (m, 4H); 7.51–7.38 (m, 4H); 5.14 (s, 2H).

LC–MS calcd for C_15_H_10_ClNO_3_ ([M + H]^+^) 288.03; found 288.69.

#### 2-((2,4-Difluorobenzyl)oxy)isoindoline-1,3-dione (**8d**)

^1^H NMR (300 MHz, CDCl_3_) δ 7.77–7.75 (m, 4H); 7.60–7.52 (3H); 5.27 (s, 2H).

LC–MS calcd for C_15_H_9_F_2_NO_3_ ([M + H]^+^) 290.05; found 290.23.

#### 2-((4-Methylbenzyl)oxy)isoindoline-1,3-dione (**8e**)

^1^H NMR (300 MHz, CDCl_3_) δ 7.84–7.73 (m, 4H); 7.44–7.20 (m, 4H); 5.19 (s, 2H); 2.37 (s, 3H).

LC–MS calcd for C_16_H_13_NO_3_ ([M + H]^+^) 268.08; found 268.27.

#### 2-([1,1′-Biphenyl]-4-ylmethoxy)isoindoline-1,3-dione (**8f**)

^1^H NMR (300 MHz, CDCl_3_) δ 7.86–7.74 (m, 4H); 7.64–7.60 (m, 6H); 7.49–7.37 (m, 3H); 5.28 (s, 2H).

LC–MS calcd for C_21_H_15_NO_3_ ([M + H]^+^) 330.10; found 330.34.

#### 2-(Naphthalen-2-ylmethoxy)isoindoline-1,3-dione (**8g**)

^1^H NMR (300 MHz, DMSO) δ 8.03–7.86 (m, 8H); 7.68–7.54 (m, 3H); 5.34 (s, 2H).

LC–MS (ESI) calculated for C_19_H_13_NO_3_ ([M + H]^+^) 304.08; found 304.31.

#### 2-Phenethoxyisoindoline-1,3-dione (**12h**)

^1^H NMR (300 MHz, CDCl_3_) δ 7.71–7.7.59 (m, 4H); 7.28–7.23 (m, 5H); 4.37 (t, *J* = 6, 2H); 3.07 (t, *J* = 6, 2H).

LC–MS calcd for C_16_H_13_NO_3_ ([M + H]^+^) 268.08; found 268.27.

#### 2-(4-Methoxyphenethoxy)isoindoline-1,3-dione (**12i**)

^1^H NMR (300 MHz, CDCl_3_) δ 7.87–7.75 (m, 4H); 7.24 (d, *J* = 9, 2H); 7.16 (d, *J* = 9, 2H); 4.42 (t, *J* = 6, 2H); 3.83 (s, 3H). 3.53 (t, *J* = 6, 2H);

LC–MS calcd for C_17_H_15_NO_4_ ([M + H]^+^) 298.10; found 298.30.

### Procedure for the Synthesis of Compound **15**

To a solution of 3-bromobenzaldehyde **14** (1 equiv) in dry toluene (3 mL/mmol), propane-1,3-diol (1.2 equiv) and *p-*toluenesulfonic acid (0,006 equiv) were added. The reaction mixture was heated at 110 °C for 18 h, and then it was controlled by TLC. The solvent was evaporated. After addition of H_2_O, the organic layer was separated, and the aqueous layer was extracted with dichloromethane (×3). The organic layer was washed with brine and dried over anhydrous Na_2_SO_4_. After removal of the solvent, the crude material was purified by column chromatography with hexane/ethyl acetate 9:1 as eluent. The desired products were obtained as colorless oils with 92% yields.

#### 2-(3-Bromophenyl)-1,3-dioxolane (**15**)

^1^H NMR (300 MHz, CDCl_3_) δ 7.53–7.38 (m, 3H); 5.74 (s, 1H); 4.09–3.98 (m, 4H).

LC–MS calcd for C_9_H_9_BrO_2_ ([M + H]^+^) 229.97; found 229.07.

### General Procedure for the Synthesis of Compounds **17j-l**

To a solution of compound **15** (1 equiv) in dry toluene (3 mL/mmol), under nitrogen flux, the suitable cyclic amines **16j**–**l** (1.2 equiv), Pd_2_(dba)_3_ (0.005 equiv), BINAP (0.015 equiv), and NaO*t*Bu (1.7 equiv) were added. The reaction mixture was stirred for 18 h at 100 °C. A TLC, with petroleum ether/ethyl acetate (9:1) as eluent, showed complete consumption of the starting material. HCl 1 N was added gradually at 0 °C until pH 1. The mixture was stirred at 0 °C; after 3 h, NaOH 1 N was added until pH 11. Ethyl acetate was added, and the crude was extracted and then washed with brine. The organic phase was dried over anhydrous Na_2_SO_4_, filtered, and concentrated under reduced pressure. The desired product was purified by silica flash chromatography by elution with petroleum ether/ethyl acetate from 95:5 to 7:3. The desired products were obtained as colorless oils with 56–68% yields.

#### 3-Morpholinobenzaldehyde (**17j**)

^1^H NMR (300 MHz, CDCl_3_) δ 9.98 (s, 1H); 7.48–7.18 (m, 3H); 3.89 (t, *J* = 6, 2H); 3.24 (t, *J* = 6, 2H).

LC–MS calcd for C_11_H_13_NO_2_ ([M + H]^+^) 192.09; found 192.22.

#### 3-(Pyrrolidin-1-yl)benzaldehyde (**17k**)

^1^H NMR (300 MHz, CDCl_3_) δ 9.94 (s, 1H); 7.67–7.55 (m, 3H); 3.33–3.29 (m, 4H); 2.05–2.00 (m, 4H).

LC–MS calcd for C_11_H_13_NO ([M + H]^+^) 176.09; found 176.22.

#### 3-(4-Methylpiperazin-1-yl)benzaldehyde (**17l**)

^1^H NMR (300 MHz, CDCl_3_) δ 9.90 (s, 1H); 7.38–7.12 (m, 3H); 3.25–3.22 (m, 4H); 2.56–2.52 (m, 4H); 2.31 (s, 3H).

LC–MS calcd for C_12_H_16_N_2_O ([M + H]^+^) 205.12; found 205.26.

### General Procedure for the Synthesis of Compounds **22m–p**

A microwave tube was charged with Pd(PPh_3_)_4_ (0.02 equiv), the proper boronic acid pinacol ester **21m**–**p** (2 equiv), and cesium carbonate (2 equiv). The tube was purged with argon three times, and then a solution of compound **14** (1 equiv) in a mixture of DME/H_2_O 2:1 .3 mL/mmol) was injected in the reaction mixture that was then heated in a microwave reactor for 30 min at 140 °C at 100 W power. A TLC, with the proper eluent, showed complete consumption of the starting material. Afterward, the reaction mixture was filtered through a plug of Celite, the filtrate was concentrated under reduced pressure, and the residue was purified by flash column chromatography with the proper eluent. The desired products were obtained as colorless oils with 67–78% yields.

#### [1,1′-Biphenyl]-3-carbaldehyde (**22m**)

^1^H NMR (300 MHz, CDCl_3_) δ 10.12 (s, 1H); 8.14 (s, 1H); 7.91–7.87 (m, 2H); 7.67–7.61 (m, 3H); 7.53–7.40 (m, 3H).

LC–MS calcd for C_13_H_10_O ([M + H]^+^) 183.07; found 183.21.

#### 3-(2-Aminopyrimidin-5-yl)benzaldehyde (**22n**)

^1^H NMR (300 MHz, DMSO) δ 10.07 (s, 1H); 8.67 (s, 2H); 8.22–8.17 (m, 1H); 8.00–7.97 (d, *J* = 9, 1H); 7.86–7.83 (d, *J* = 9, 1H); 7.66 (m, 1H); 6.89 (s, 2H).

LC–MS calcd for C_11_H_9_N_3_O ([M + H]^+^) 200.07; found 200.20.

#### 4′-(Trifluoromethyl)-[1,1′-biphenyl]-3-carbaldehyde (**22o**)

^1^H NMR (300 MHz, CDCl_3_) δ 10.12 (s, 1H); 8.13 (s, 1H); 7.95–7.86 (m, 2H); 7.75 (s, 4H); 7.69–7.64 (m, 1H).

LC–MS calcd for C_14_H_9_F_3_O ([M + H]^+^) 251.06; found 251.21.

#### 4′-Fluoro-[1,1′-biphenyl]-3-carbaldehyde (**22p**)

^1^H NMR (300 MHz, CDCl_3_) δ 10.11 (s, 1H); 8.08 (s, 1H); 8.07–7.82 (m, 2H); 7.66–7.58 (m, 3H); 7.22–7.16 (m, 2H).

LC–MS calcd for C_13_H_9_FO ([M + H]^+^) 201.17; found 201.45.

### General Procedure for the Synthesis of Compounds **18j**–**l**, **23m–p**, and **27q–u**

LiAlH_4_ (4 equiv) was added in a round-bottomed flask containing dry THF (4 mL/mmol) under nitrogen flux, then the reaction mixture was stirred at 0 °C, and the appropriate aldehyde **17j**–**l**, **22m-**–**p**, or **26q**–**u** (1 equiv) was added. After 30 min, a TLC, with dichloromethane/methanol (95:5) as eluent, showed complete consumption of the starting material. A solution of NaOH 1 N was added dropwise until pH 11 at 0 °C. Ethyl acetate was added, and the crude was extracted and then washed with brine. The organic phase was dried over anhydrous Na_2_SO_4_, filtered, and concentrated under reduced pressure. The desired product was purified by silica flash chromatography by elution with dichloromethane/methanol from 99:1 to 95:5. The desired products were obtained as colorless oils with 43–82% yields.

#### (3-Morpholinophenyl)methanol (**18j**)

^1^H NMR (300 MHz, CDCl_3_) δ 7.24 (d, *J* = 6, 1H); 6.90–6.81 (m, 3H); 4.59 (s, 2H); 3.82–3.74 (m, 4H); 3.12–3.08 (m, 5H).

LC–MS calcd for C_11_H_15_NO_2_ ([M – H]^−^) 192.11; found 192.24.

#### (3-(Pyrrolidin-1-yl)phenyl)methanol (**18k**)

^1^H NMR (300 MHz, CDCl_3_) δ 7.25 (t, *J*_1_ = 9, *J*_2_ = 15, 1H); 6.90–6.81 (m, 3H); 4.59 (s, 2H); 3.79 (s, 1H); 3.82 (t, *J* = 6, 4H); 3.12 (t, *J* = 6, 4H).

LC–MS calcd for C_11_H_15_NO ([M – H]^−^) 176.11; found 176.24.

#### (3-(4-Methylpiperazin-1-yl)phenyl)methanol (**18l**)

^1^H NMR (300 MHz, CDCl_3_) δ 7.24 (t, *J* = 9, 1H); 6.94 (s, 1H); 6.85 (d, *J* = 9, 2H); 4.64 (s, 2H); 3.53–3.17 (m, 4H); 2.88 (s, 1H); 2.57–2.49 (m, 4H); 2.35 (s, 3H).

LC–MS calcd for C_12_H_18_N_2_O ([M – H]^−^) 205.14; found 205.28.

#### [1,1′-Biphenyl]-3-ylmethanol (**23m**)

^1^H NMR (300 MHz, CDCl_3_) δ 7.73–7.56 (m, 4H); 7.47–7.37 (m, 5H); 4.67 (s, 2H); 3.65 (s, 1H).

LC–MS calcd for C_13_H_12_O ([M – H]^−^) 183.08; found 183.23.

#### (3-(2-Aminopyrimidin-5-yl)phenyl)methanol (**23n**)

^1^H NMR (300 MHz, DMSO) δ 8.67 (s, 2H); 7.53–7.26 (m, 4H); 6.76 (s, 2H); 5.22 (t, *J* = 6, 1H); 4.54 (d, *J* = 6, 2H).

LC–MS calcd for C_11_H_11_N_3_O ([M – H]^−^) 200.09; found 200.22.

#### (4′-(Trifluoromethyl)-[1,1′-biphenyl]-3-yl)methanol (**23o**)

^1^H NMR (300 MHz, CDCl_3_) δ 7.72 (s, 4H); 7.69–7.64 (m, 4H); 4.82 (s, 2H); 3.71 (s, 1H).

LC–MS calcd for C_14_H_11_F_3_O ([M – H]^−^) 251.07; found 251.23.

#### (4′-Fluoro-[1,1′-biphenyl]-3-yl)methanol (**23p**)

^1^H NMR (300 MHz, DMSO) δ 7.70–7.64 (m, 2H); 7.58 (s, 1H); 7.51–7.41 (m, 2H); 7.32–7.27 (m, 3H); 5.27 (t, *J* = 6, 1H); 4.57 (d, *J* = 6, 2H).

LC–MS calcd for C_13_H_11_FO ([M – H]^−^) 201.07; found 201.22.

#### Pyridin-3-ylmethanol (**27q**)

^1^H NMR (300 MHz, CDCl_3_) δ 8.47–8.39 (m, 2H); 7.73–7.70 (m, 1H); 7.27–7.24 (m, 1H); 4.68 (s, 2H); 3.88 (s, 1H).

LC–MS calcd for C_6_H_7_NO ([M – H]^−^) 108.05; found 108.12.

#### Benzofuran-2-ylmethanol (**27r**)

^1^H NMR (400 MHz, CDCl_3_) δ 7.58 (d, *J* = 6, 1H); 7.49 (d, *J* = 6, 1H); 7.33–7.23 (m, 2H); 6.69 (s, 1H); 4.80 (6, *J* = 6, 2H); 2.05 (t, *J* = 6, 1H).

LC–MS calcd for C_9_H_8_O_2_ ([M – H]^−^) 147.05; found 147.15.

#### Benzo[*b*]thiophen-2-ylmethanol (**27s**)

^1^H NMR (300 MHz, CDCl_3_) δ 7.86–7.74 (m, 2H); 7.40–7.31 (m, 2H); 7.26–7.23 (m, 1H); 4.97 (s, 2H); 3.96 (s, 1H).

LC–MS calcd for C_9_H_8_OS ([M – H]^−^) 163.02; found 163.22.

#### Thiophen-2-ylmethanol (**27t**)

^1^H NMR (300 MHz, CDCl_3_) δ 7.40 (d, *J* = 9, 1H); 6–96-6.90 (m, 1H); 6.85–6.79 (m, 1H); 4.85 (s, 1H); 3.85 (s, 1H).

LC–MS calcd for C_5_H_6_OS ([M – H]^−^) 113.01; found 113.16.

#### (4-Methoxy-3,5-dimethylpyridin-2-yl)methanol (**27u**)

^1^H NMR (300 MHz, CDCl_3_) δ 8.45 (s, 1H); 5.07 (s, 1H); 3.75 (s, 1H); 3.68 (s, 1H); 2.93 (s, 6H).

LC–MS calcd for C_9_H_13_NO_2_ ([M – H]^−^) 166.09; found 166.20.

### General Procedure for the Synthesis of Compounds **19j**–**l**, **24m**–**p**, and **28q**–**u**

The appropriate alcohol (1 equiv) (**18j**–**l**, **23m**–**p**, **27q**–**u**) was dissolved in dry THF (3 mL/mmol) under nitrogen flux, and then *N*-hydroxyphtalimide **7** (1 equiv), PPh_3_ (1.1 equiv), and DEAD (1 equiv) were added at room temperature. The reaction mixture was stirred at room temperature for 18 h. A TLC, with the proper eluent, showed complete consumption of the starting material. The solvent was evaporate under a vacuum, and the product was obtained by trituration with MeOH as white powder with 67–88% yields.

#### 2-((3-Morpholinobenzyl)oxy)isoindoline-1,3-dione (**20j**)

^1^H NMR (300 MHz, CDCl_3_) δ 7.74–7.63 (m, 4H); 7.19 (t, *J* = 6, 1H); 7.07 (s, 1H); 6.94–6.82 (m, 2H); 5.12 (s, 2H); 3.82–3.74 (m, 4H); 3.12–3.08 (m, 4H).

LC–MS calcd for C_19_H_18_N_2_O_4_ ([M + H]^+^) 339.12; found 339.35.

#### 2-((3-(Pyrrolidin-1-yl)benzyl)oxy)isoindoline-1,3-dione (**20k**)

^1^H NMR (300 MHz, CDCl_3_) δ 7.77–7.66 (m, 4H); 7.17 (t, *J* = 6, 1H); 6.77–6.58 (m, 2H); 6.53–6.49 (m, 1H); 5.16 (s, 2H); 3.36 (t, *J* = 6, 4H); 1.97 (t, *J* = 6, 4H).

LC–MS calcd for C_19_H_18_N_2_O_3_ ([M + H]^+^) 323.13; found 323.35.

#### 2-((3-(4-Methylpiperazin-1-yl)benzyl)oxy)isoindoline-1,3-dione (**20l**)

^1^H NMR (300 MHz, CDCl_3_) δ 7.84–7.72 (m, 4H); 7.29–7.24 (m, 1H); 7.14 (s, 1H); 7.01–6.91 (m, 2H); 5.29 (s, 2H); 3.29 (t, *J* = 6, 4H); 2.65 (t, *J* = 6, 4H); 2.41 (s, 3H).

LC–MS calcd for C_20_H_21_N_3_O_3_ ([M + H]^+^) 352.15; found 352.39.

#### 2-([1,1′-Biphenyl]-3-ylmethoxy)isoindoline-1,3-dione (**24m**)

^1^H NMR (300 MHz, CDCl_3_) δ 8.14 (s, 1H); 7.91–7.78 (m, 6H); 7.67–7.61 (m, 3H); 7.53–7.40 (m, 3H); 5.31 (s, 2H).

LC–MS calcd for C_21_H_15_NO_3_ ([M + H]^+^) 330.10; found 330.34.

#### 2-((3-(2-Aminopyrimidin-5-yl)benzyl)oxy)isoindoline-1,3-dione (**24n**)

^1^H NMR (300 MHz, DMSO) δ 8.56 (s, 2H); 7.86 (s, 4H); 7.67–7.46 (m, 4H); 7.47 (d, *J* = 6, 2H); 6.81 (s, 2H); 5.24 (s, 2H).

LC–MS calcd for C_19_H_14_N_4_O_3_ ([M + H]^+^) 347.10; found 347.33.

#### 2-((4′-(Trifluoromethyl)-[1,1′-biphenyl]-3-yl)methoxy)isoindoline-1,3-dione (**24o**)

^1^H NMR (300 MHz, CDCl_3_) δ 7.85–7.51 (m, 8H); 5.32 (s, 2H).

LC–MS calcd for C_22_H_14_F_3_NO_3_ ([M + H]^+^) 398.09; found 398.34.

#### 2-((4′-Fluoro-[1,1′-biphenyl]-3-yl)methoxy)isoindoline-1,3-dione (**24p**)

^1^H NMR (400 MHz, DMSO) δ 8.01–7.95 (m, 1H); 7.86 (s, 4H); 7.73–7.67 (m, 3H); 7.51–7.47 (m, 2H); 7.31 (t, *J* = 6, 2H); 5.26 (s, 2H).

LC–MS calcd for C_21_H_14_FNO_3_ ([M + H]^+^) 348.09; found 348.33.

#### 2-(Pyridin-3-ylmethoxy)isoindoline-1,3-dione (**28q**)

^1^H NMR (300 MHz, CDCl_3_) δ 8.71 (s, 1H); 8.66–8.64 (m, 2H); 8.02–7.98 (m, 1H); 7.86–7.75 (m, 4H); 7.41–7.36 (m, 1H); 5.26 (s, 2H).

LC–MS calcd for C_14_H_10_N_2_O_3_ ([M + H]^+^) 255.06; found 255.24.

#### 2-(Benzofuran-2-ylmethoxy)isoindoline-1,3-dione (**28r**)

^1^H NMR (300 MHz, DMSO) δ 7.87–7.81 (m, 4H); 7.65–7.57 (m, 2H); 7.39–7.43 (m, 1H); 7.25–7.21 (m, 1H); 7.11 (s, 1H) 5.31 (s, 2H).

LC–MS calcd for C_17_H_11_NO_4_ ([M + H]^+^) 294.06; found 294.27.

#### 2-(Benzo[*b*]thiophen-2-ylmethoxy)isoindoline-1,3-dione (**28s**)

^1^H NMR (300 MHz, CDCl_3_) δ 7.85–7.74 (m, 6H); 7.45 (S, 1H); 7.38–7.35 (m, 2H); 5.51 (s, 2H).

LC–MS calcd for C_17_H_11_NO_3_S ([M + H]^+^) 310.04; found 310.33.

#### 2-(Thiophen-2-ylmethoxy)isoindoline-1,3-dione (**28t**)

^1^H NMR (400 MHz, CDCl_3_) δ 7.85–7.75 (m, 4H); 7.43 (d, *J* = 4, 1H); 7.22 (d, *J* = 4, 1H); 7.02 (t, *J* = 4, 1H); 5.40 (s, 2H).

LC–MS calcd for C_13_H_9_NO_3_S ([M + H]^+^) 260.03; found 260.28.

#### 2-((4-Methoxy-3,5-dimethylpyridin-2-yl)methoxy)isoindoline-1,3-dione (**28u**)

^1^H NMR (300 MHz, CDCl_3_) δ 8.15 (s, 1H); 7.89–7.83 (m, 4H); 5.35 (s, 2H); 3.83 (s, 3H); 2.57 (s, 3H); 2.27 (s, 3H).

LC–MS calcd for C_17_H_16_N_2_O_4_ ([M + H]^+^) 313.11; found 313.32.

### General Procedure for the Synthesis of Compounds **9a**–**g**, **13h**–**i**, **21j**–**l**, **25m**–**p**, and **29q**–**u**

The appropriate intermediate (1 equiv) (**8a**–**g**, **12h**–**i**, **20j**–**l**, **24m**–**p**, **28q**–**u**) was dissolved in DCM (1 m/mmol), and then hydrazine (2 equiv) was added at room temperature. The reaction mixture was stirred at room temperature for 2 h. Afterward, a TLC with the proper eluent showed complete consumption of the starting material. The reaction mixture was filtered, and the solution containing the product was dried under a vacuum. The desired products (**9a**–**g**) were obtained as colorless oil with 92–97% yields. Compounds **13h**–**i**, 21j-l, **25m**–**p**, and **29q**–**u** were purified by flash column chromatography with the proper eluent. The desired products were obtained as colorless oils with 83–97% yields.

#### *O*-(3-Bromobenzyl)hydroxylamine (**9a**)

^1^H NMR (300 MHz, CDCl_3_) δ 7.64–7.60 (m, 2H); 7.46–7.37 (m, 2H); 5.66 (s, 2H); 4.59 (s, 2H).

LC–MS calcd for C_7_H_8_BrNO ([M + H]^+^) 202.97; found 202.04.

#### *O*-(4-Fluorobenzyl)hydroxylamine (**9b**)

^1^H NMR (300 MHz, DMSO) δ 7.34–7.17 (m, 4H); 5.21 (s, 2H) 5.87 (s, 2H) 4.45 (s, 2H).

LC–MS calcd for C_7_H_8_FNO ([M + H]^+^) 142.05; found 142.14.

#### *O*-(4-Chlorobenzyl)hydroxylamine (**9c**)

^1^H NMR (300 MHz, CDCl_3_) δ 7.51–7.38 (m, 4H); 5.87 (s, 2H) 4.34 (s, 2H).

LC–MS calcd for C_7_H_8_ClNO ([M + H]^+^) 158.02; found 158.59.

#### *O*-(2,4-Difluorobenzyl)hydroxylamine (**9d**)

^1^H NMR (300 MHz, CDCl_3_) δ 7.60–7.52 (3H); 5.66 (s, 2H); 4.51 (s, 2H).

LC–MS calcd for C_7_H_7_F_2_NO ([M + H]^+^) 160.04; found 160.13.

#### *O*-(4-Methylbenzyl)hydroxylamine (**9e**)

^1^H NMR (300 MHz, CDCl_3_) δ 7.21–7.13 (m, 4H); 5.99 (s, 2H);4.51 (s, 2H); 2.29 (s, 3H).

LC–MS calcd for C_8_H_11_NO ([M + H]^+^) 138.08; found 138.17.

#### *O*-([1,1′-Biphenyl]-4-ylmethyl)hydroxylamine (**9f**)

^1^H NMR (300 MHz, CDCl_3_) δ 7.64–7.61 (m, 4H); 7.49–7.44 (m, 4H); 7.40–7.35 (m, 1H); 5.46 (s, 2H); 4.76 (s, 2H).

#### *O*-(Naphthalen-2-ylmethyl)hydroxylamine (**9g**)

^1^H NMR (300 MHz, CDCl_3_) δ 7.97–7.47 (m, 7H); 5.01 (s, 2H); 4.68 (s, 2H).

^13^C NMR (100.6 MHz, CDCl_3_) δ: 133.52; 133.10; 131.69; 129.03; 128.78; 128.51; 128.11; 127.23; 127.09; 126.99; 76.25.

HRMS (ESI) calculated for C_11_H_11_NO ([M + H]^+^) 174.08414; found 174.08431.

#### *O*-Phenethylhydroxylamine (**13h**)

^1^H NMR (300 MHz, CDCl_3_) δ 7.43–7.22 (m, 5H); 5.45 (s, 2H); 3.92 (t, *J* = 8, 2H); 2.93 (t, *J* = 8, 2H).

^13^C NMR (100.6 MHz, CDCl_3_) δ: 151.42; 138.24; 129.24; 119.61; 115.75; 115.58; 55.05; 48.91.

HRMS (ESI) calculated for C_8_H_11_NO ([M + H]^+^) 138.07613; found 138.076504.

#### *O*-(4-Methoxyphenethyl)hydroxylamine (**13i**)

^1^H NMR (300 MHz, CDCl_3_) δ 7.14 (d, *J* = 8, 2H); 6.84 (d, *J* = 8, 2H); 4.14 (t, *J* = 8, 2H); 3.77 (s, 3H); 2.86 (t, *J* = 8, 2H).

^13^C NMR (100.6 MHz, CDCl_3_) δ: 151.42; 138.24; 129.24; 119.61; 115.75; 115.58; 55.05; 48.91; 46.11.

HRMS (ESI) calculated for C_9_H_13_NO_2_ ([M + H]^+^) 168.09464; found 168.094503.

#### *O*-(3-Morpholinobenzyl)hydroxylamine (**20j**)

^1^H NMR (400 MHz, CDCl_3_) δ: 7.20 (t, *J* = 8, 1H); 6.86–6.79 (m, 3H); 5.12 (s, 2H); 4.59 (s, 2H); 3.78 (t, *J* = 4, 4H); 3.09 (t, *J* = 4, 4H).

^13^C NMR (100.6 MHz, CDCl_3_) δ: 151.50; 138.41; 129.33; 119.99; 115.42; 115.32; 78.28; 66.92; 49.36; 49.28; 21.97; 21.93.

HRMS (ESI) calculated for C_11_H_16_N_2_O_2_ ([M + H]^+^) 209.12123; found 209.121569.

#### *O*-(3-(Pyrrolidin-1-yl)benzyl)hydroxylamine (**20k**)

^1^H NMR (300 MHz, CDCl_3_) δ 7.24 (t, *J* = 9, 1H); 6.68 (d, *J* = 4, 1H); 6.59–6.54 (m, 2H); 5.09 (s, 2H); 4.69 (s, 2H); 3.34–3.30 (m, 4H); 2.05–2.00 (m, 4H).

^13^C NMR (100.6 MHz, CDCl_3_) δ: 148.20; 138.16; 129.32; 115.43; 111.46; 111.43; 78.70; 47.68; 25.52.

HRMS (ESI) calculated for C_11_H_16_N_2_O ([M + H]^+^) 193.12634; found 193.126775.

#### *O*-(3-(4-Methylpiperazin-1-yl)benzyl)hydroxylamine (**20l**)

^1^H NMR (300 MHz, CDCl_3_) δ 7.18 (t, *J* = 6, 1H); 6.37 (s, 1H); 6.78 (t, *J* = 6, 2H); 4.95 (s, 2H); 4.57 (s, 2H); 3.15 (t, *J* = 6, 4H); 2.49 (t, *J* = 6, 4H); 2.27 (s, 3H).

^13^C NMR (100.6 MHz, CDCl_3_) δ: 151.42; 138.24; 129.24; 119.61; 115.75; 115.58; 27.31.

HRMS (ESI) calculated for C_12_H_19_N_3_O ([M + H]^+^) 222.15284; found 222.15288.

#### *O*-([1,1′-Biphenyl]-3-ylmethyl)hydroxylamine (**25m**)

^1^H NMR (300 MHz, CDCl_3_) δ 7.73–7.67 (m, 4H); 7.56–7.37 (m, 5H); 5.06 (s, 2H); 4.67 (s, 2H).

^13^C NMR (100.6 MHz, CDCl_3_) δ: 140.99; 140.11; 134.96; 129.79; 129.48; 128.68; 128.19; 128.02; 127.85; 127.22; 76.20.

HRMS (ESI) calculated for C_13_H_13_NO ([M + H]^+^) 200.09971; found 200.09986.

#### 5-(3-((Aminooxy)methyl)phenyl)pyrimidin-2-amine (**25n**)

^1^H NMR (300 MHz, CDCl_3_) δ 8.73–8.69 (m, 2H); 8.07–8.04 (m, 1H); 7.66–7.61 (m, 1H); 5.11 (s, 2H); 4.67 (s, 2 h).

^13^C NMR (100.6 MHz, CDCl_3_) δ: 147.64; 140.90; 131.90; 124.49; 73.07.

HRMS (ESI) calculated for C_11_H_12_N_4_O ([M + H]^+^) 217.10114; found 217.10192.

#### *O*-((4′-(Trifluoromethyl)-[1,1′-biphenyl]-3-yl)methyl)hydroxylamine (**25o**)

^1^H NMR (300 MHz, CDCl_3_) δ 7.94–7.78 (m, 6H); 7.61–7.48 (m, 2H); 5.10 (s, 2H); 4.56 (s, 2H).

^13^C NMR (100.6 MHz, CDCl_3_) δ: 144.11; 139.38; 135.29; 130.00; 129.65; 128.30; 128.19; 128.05; 126.34; 76.05.

HRMS (ESI) calculated for C_14_H_12_F_3_NO ([M + H]^+^) 268.08714; found 268.08786.

#### *O*-((4′-Fluoro-[1,1′-biphenyl]-3-yl)methyl)hydroxylamine (**25p**)

^1^H NMR (300 MHz, CDCl_3_) δ 8.08 (s, 1H); 8.07–7.82 (m, 2H); 7.66–7.58 (m, 5H); 7.22–7.16 (m, 2H); 4.91 (s, 2H); 4.62 (s, 2H).

^13^C NMR (100.6 MHz, CDCl_3_) δ: 164.92; 139.94; 136.58; 134.99; 129.82; 129.28; 129.20; 128.65; 127.96; 127.80; 116.40; 116.18; 87.15; 76.16.

HRMS (ESI) calculated for C_13_H_12_FNO ([M + H]^+^) 218.09034; found 218.09089.

#### *O*-(Pyridin-3-ylmethyl)hydroxylamine (**29q**)

^1^H NMR (300 MHz, CDCl_3_) δ 8.73–8.69 (m, 2H); 8.07–8.04 (m, 1H); 7.66–7.61 (m, 1H); 5.11 (s, 2H); 4.71 (s, 2H).

^13^C NMR (100.6 MHz, CDCl_3_) δ: 147.64; 140.90; 131.90; 125.49; 73.07.

HRMS (ESI) calculated for C_6_H_8_N_2_O ([M + H]^+^) 125.06371; found 125.06305.

#### *O*-(Benzofuran-2-ylmethyl)hydroxylamine (**29r**)

^1^H NMR (300 MHz, CDCl_3_) δ 7.59 (d, *J* = 3; 1H); 7.51 (d, *J* = 3; 1H); 7.34–7.22 (m, 2H); 6.77 (s, 1H); 5.60 (s, 2H); 4.81 (s, 2H).

^13^C NMR (100.6 MHz, CDCl_3_) δ: 155.35; 150.50; 127.92; 125.89; 123.63; 122.30; 111.86; 109.90; 68.24.

HRMS (ESI) calculated for C_9_H_9_NO_2_ ([M + H]^+^) 164.06333; found 164.06333.

#### *O*-(Benzo[*b*]thiophen-2-ylmethyl)hydroxylamine (**29s**)

^1^H NMR (400 MHz, DMSO) δ 7.94 (d, *J* = 4, 1H); 7.81 (d, *J* = 4, 2H); 7.38–7.31 (m, 3H); 6.24 (s, 2H); 4.82 (s, 2H); 4.76.

^13^C NMR (100.6 MHz, CDCl_3_) δ: 142.62; 139.98; 139.58; 124.76; 124.73; 123.99; 123.28; 122.88; 72.40.

HRMS (ESI) calculated for C_9_H_9_NOS ([M + H]^+^) 180.04053; found 180.04086.

#### *O*-(Thiophen-2-ylmethyl)hydroxylamine (**29t**)

^1^H NMR (400 MHz, DMSO) δ 7.69 (d, *J* = 4, 1H); 7.28 (s, 1H); 7.09 (t, *J* = 4, 1H); 5.99 (s, 2H); 5.24 (s, 2H).

^13^C NMR (100.6 MHz, CDCl_3_) δ: 135.46; 130.61; 129.35; 127.71; 70.06.

HRMS (ESI) calculated for C_5_H_7_NOS ([M + H]^+^) 130.02481; found 130.02486.

#### *O*-((4-Methoxy-3,5-dimethylpyridin-2-yl)methyl)hydroxylamine (**29u**)

^1^H NMR (300 MHz, CDCl_3_) δ 8.26 (s, 1H); 5.63 (s, 2H); 4.85 (s, 2H); 3.78 (s, 3H); 2.29 (s, 3H); 2.27 (s, 3H) .

^13^C NMR (100.6 MHz, CDCl_3_) δ: 163.08; 153.95; 148.16; 124.79; 124.35; 58.86; 12.28.

HRMS (ESI) calculated for C_9_H_14_N_2_O_2_ ([M + H]^+^) 183.10557; found 183.10586.

### General Procedure for the Synthesis of Compounds **10a**–**g**

The appropriate intermediate **9a**–**g** (1 equiv) was dissolved in ethyl ether (1 mL/mmol), and then a hydrochloric solution 4 N in dioxane (0.5 mL/mmol) was gradually added. The reaction mixture was stirred at room temperature for 30 min. Afterward, the precipitate was filtered, and the product was obtained as white powder salt with 92–98% yields.

#### *O*-(3-Bromobenzyl)hydroxylammonium Chloride (**10a**)

^1^H NMR (300 MHz, DMSO) δ 11.12 (s, 3H); 7.64–7.60 (m, 2H); 7.46–7.37 (m, 2H); 5.05 (s, 2H).

^13^C NMR (100.6 MHz, CDCl_3_) δ: 136.94; 132.35; 132.22; 131.31; 128.63; 122.17; 75.07.

HRMS (ESI) calculated for C_7_H_9_BrClNO ([M + H]^+^) 203.21783; found 203.21786.

#### *O*-(4-Fluorobenzyl)hydroxylammonium Chloride (**10b**)

^1^H NMR (300 MHz, CDCl_3_) δ 11.04 (s, 3H); 7.51–7.46 (m, 2H); 7.29–7.23 (m, 2H); 5.03 (s, 2H).

^13^C NMR (100.6 MHz, CDCl_3_) δ: 164.18; 161.74: 132.22; 132.14; 130.59; 130.56; 116.10; 115.88; 75.29.

HRMS (ESI) calculated for C_7_H_9_ClFNO ([M + H]^+^) 142.03571; found 142.03586.

#### *O*-(4-Chlorobenzyl)hydroxylammonium Chloride (**10c**)

^1^H NMR (400 MHz, DMSO) δ 11.23 (s, 3H); 7.51–7.38 (m, 4H); 4.76 (s, 2H).

^13^C NMR (100.6 MHz, CDCl_3_) δ: 134.23; 133.22; 131.61; 129.13; 75.15.

HRMS (ESI) calculated for C_7_H_9_Cl_2_NO ([M + H]^+^) 158.00613; found 158.00686.

#### *O*-(2,4-Difluorobenzyl)hydroxylammonium Chloride (**10d**)

^1^H NMR (400 MHz, DMSO) δ 11.18 (s, 3H); 7.62–7.56 (m, 1H); 7.37–7.31 (m, 1H); 7.19–7.14 (m, 1H); 5.10 (s, 2H).

^13^C NMR (100.6 MHz, CDCl_3_) δ: 162.92; 160.43; 134.25; 134.20; 134.15; 134.09; 117.93; 117.78; 112.48; 112.45; 112.27; 112.24; 105.02; 104.76; 104.51; 69.44; 69.41.

HRMS (ESI) calculated for C_7_H_8_ClF_2_NO ([M + H]^+^) 160.02624; found 160.02686.

#### *O*-(4-Methylbenzyl)hydroxylammonium Chloride (**10e**)

^1^H NMR (400 MHz, DMSO) δ 11.11 (s, 3H); 7.44–7.20 (m, 4H); 5.03 (s, 2H); 2.37 (s, 3H).

^13^C NMR (100.6 MHz, CDCl_3_) δ: 139.10; 131.11; 129.86; 129.66; 76.13; 21.32.

HRMS (ESI) calculated for C_8_H_12_ClNO ([M + H]^+^) 138.06073; found 138.06086.

#### *O*-([1,1′-Biphenyl]-4-ylmethyl)hydroxylammonium Chloride (**10f**)

^1^H NMR (400 MHz, DMSO) δ 11.13 (s, 3H); 7.52–7.49 (m, 5H); 7.42–7.38 (m, 4H); 4.79 (s, 2H).

^13^C NMR (100.6 MHz, CDCl_3_) δ: 140.91; 139.70; 135.47; 129.23; 128.12; 127.92; 127.64; 79.03.

HRMS (ESI) calculated for C_13_H_14_ClNO ([M + H]^+^) 235.0764; found 200.2286.

#### *O*-(Naphthalen-2-ylmethyl)hydroxylammonium Chloride (**10g**)

^1^H NMR (400 MHz, DMSO) δ 11.23 (s, 3H); 7.97–7.47 (m, 8H); 5.01 (s, 2H).

^13^C NMR (100.6 MHz, CDCl_3_) δ: 133.52; 133.10; 131.69; 129.03; 128.78; 128.51; 128.11; 127.23; 127.09; 126.99; 76.25.

HRMS (ESI) calculated for C_11_H_12_ClNO ([M + H]^+^) 174.67301; found 174.67311.

## Materials

PLP, pyridoxine, l-alanine, sodium glyoxylate, rabbit muscle l-lactic dehydrogenase (LDH), β-nicotinamide adenine dinucleotide reduced form (NADH), isopropyl-β-d-thiogalactoside (IPTG), and imidazole were all purchased from Sigma. Ham’s F12 Glutamax medium and Zeocin were purchased from Invitrogen. Geneticin was purchased from Gibco. Protease Inhibitor Cocktail EDTA-free and Protease Inhibitor Cocktail Complete Mini were purchased from Roche. The clones of Chinese hamster ovary cells stably expressing glycolate oxidase (CHO-GO) and AGTwt (CHO-GO-AGTwt), or the variants (CHO-GO-G41R, CHO-GO-AGTMi, CHO-GO-F152IMi, CHO-GO-G170RMi, CHO-GO-I244TMi), and the rabbit polyclonal anti-AGT human antibody were kindly provided by Prof. C.J. Danpure (University College London). Compounds **1**, **2**, **3**, **4**, and **5** were purchased from Sigma-Aldrich. All other chemicals were of the highest purity available.

## Methods

### Docking Studies

Molecular docking has been performed using the MOE Dock Tool available on MOE 2018.0101(Chemical Computing Group ULC, Montreal, QC, Canada) as previously reported.^53^ Briefly, the crystal structure of human AGT (PDB ID:1H0C)^1^ and the ligand (compound **1**) have been prepared and protonated using the Protonated 3D Tool and the docking site selected by using the MOE Site Finder Tool. Then, a collection of ligand conformations using the bond rotation method has been generated, placed in the selected site by the Triangle Matcher method, and ranked using the London dG scoring function, which estimates the free energy of binding of the ligand from a given pose. Finally, the Refinement tool has been used for energy minimization of the pocket before rescoring the poses with Affinity dG, which estimates the enthalpic contribution to the free energy of binding. The top 10 poses for each ligand generated by the MOE Dock Tool were analyzed by visual inspection.

### p*K*_a_ Measurement

The p*K*_a_ of compound **10b** was performed spectrophotometrically by using a Sirius T3 instrument (Pion Inc., Forest Row, UK).^54,55^ After calibration of the glass-electrode in ionic strength adjusted water (0.15 M for KCl addition),^56^ 15 μL of a 10 mM solution of the compound in DMSO was spiked into 1.5 mL of ionic strength adjusted water. The sample solution was alkalimetrically titrated in the pH range from 2.0 to 9.0 at 25 ± 0.5 °C. Before titration, (i) a dark spectrum (i.e., a spectrum collected when lamp was off), accounting for detector noise, and (ii) a reference spectrum in the solution matrix (i.e., water with 1% DMSO) were recorded. Those were taken into account to return absorbance of the sample at each wavelength. The initial estimates of p*K*_a_ values were iteratively refined by means of the Target Factor Analysis^57^ procedure, implemented in the SiriusT3Refine v. 1.1.3.0 software (Pion Inc., Forest Row, UK), to return the p*K*_a_ of **10b**. Reported is the mean value of three replicates with standard deviation.

### Protein Expression and Purification

AGTwt and pathogenic variants in their His-tagged form were expressed in *Escherichia coli* BL21 strain and purified by the procedure already described.^58^ Briefly, after the expression of the protein upon induction with 0.1 mM IPTG for 15 h at 30 °C, cells were lysed and the suspensions were centrifuged at 30,000*g* for 30 min at 4 °C. The sample was loaded on a HisPrep FF 16/10 column (GE Healthcare), and the eluted fractions, after addition of 100 μM PLP, were concentrated using Vivaspin Turbo 15 concentrators (Sartorius). Protein concentration was determined by absorbance spectroscopy using an extinction coefficient of 9.54 × 10^4^ M^–1^ cm^–1^ at 280 nm. The PLP content was determined by releasing the coenzyme in 0.1 M NaOH and using ε = 6600 M^–1^ cm^–1^at 388 nm.^3^

### Activity Assays

The transaminase activity of purified enzymes and cellular lysates was assayed as previously described.^3,34^ Briefly, 100 μg of the cell lysate was incubated with 0.5 M l-alanine and 10 mM glyoxylate at 25 °C for 10–60 min in 0.1 M potassium phosphate buffer (KP), pH 7.4, in the presence of 100 μM PLP. The reactions were stopped by adding TCA 10% (v/v), and pyruvate production was measured using a spectrophotometric assay coupled with lactate dehydrogenase.

### Inhibition Studies

IC_50_ values were determined by incubating AGTwt and the G41R variant in the presence of increasing concentrations of each inhibitor (range 100 nM–1 mM) followed by the measurement of residual transaminase activity. Each mixture contained 0.1 or 0.2 μM purified recombinant enzyme, 30 μM PLP, inhibitor, and 10 mM glyoxylate in 0.1 M KP, pH 7.4. The reaction, started by adding 37 mM or 50 mM l-alanine to AGTwt and the G41R variant, respectively, was stopped by adding 10% TCA (v/v) followed by the measurement of pyruvate formation using the assay coupled with LDH.

The slow-binding inhibition kinetics of AGT ligands was evaluated using the progression curve method.^59^ AGTwt (0.05 μM) or the G41R variant (0.1 μM) was added to a reaction mixture (200 mM l-alanine, 10 mM glyoxylate, and 100 μM PLP in KP 0.1 M, pH 7.4), containing each inhibitor (nM−μM), and incubated at 25 °C. At different time points, aliquots were collected, the reaction was stopped by TCA 10% (v/v), and pyruvate produced was measured. Data for each progression curve were fitted to the integrated rate equation for slow binding inhibitors:
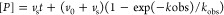
1where *v*_0_ represents the initial rate, *v*_s_ the steady-state rate, and *k*_obs_ the apparent first-order rate constant characterizing the formation of the steady-state enzyme–inhibitor complex. The obtained *k*_obs_ values were further analyzed for a one-step association mechanism:
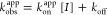
2where *k*_off_ and *k*_on_ are the dissociation and association rate constants, respectively. Intercept and slope values, obtained by linear regression of the *k*_obs_ versus inhibitor concentration plot (eq 2), yielded the association and dissociation rate constants *k*_on_ and *k*_off_, respectively, and the inhibition constant (eq 3)
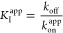
3*k*_on_^app^ was determined from the slope of the plot and then corrected for substrate competition using the following equation:
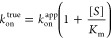
4

The *K*_I_^true^ value was obtained from the following equation:
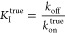
5

For some inhibitors (compounds **10g**, **10b**, **25m**, and **20k** on wild-type AGT, and compounds **10g** and **10b** on the G41R variant), it was not possible to define experimental conditions suitable to monitor the binding equilibrium to the enzyme. In the latter cases, the kinetic parameters of the inhibition were determined by using the relationship
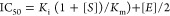
6

### Cell Culture, Treatment, and Lysis

Clones of CHO-GO cells were cultured in Ham’s F12 Glutamax medium supplemented with fetal bovine serum (10%, v/v), penicillin (100 U/mL), and streptomycin (100 mg/mL) at 37 °C in a 5% CO_2_ humidified environment. The expression of AGT and GO was maintained by adding G-418 (0.8 mg/mL) and Zeocin (0.4 mg/mL), respectively, to the culture medium. To test the effects of each compound, cells were seeded in Petri dishes at the density of 9000/cm^2^ and cultured for 7 days with 50 μM of each inhibitor in the presence or in the absence of 10 μM PN. At the end of treatment, cells were harvested and lysed by freeze/thawing (five cycles) in phosphate buffer saline (PBS) pH 7.2 supplemented with 100 μM PLP and the protease inhibitor cocktail (Complete Mini, Roche). The whole-cell extract was separated by centrifugation (13,200*g*, 10 min, 4 °C) to obtain the soluble fraction. Protein concentration in the soluble fraction was measured in quadruplicate using the Bradford protein assay.

### Western Blot Analyses

Three micrograms of the soluble cell lysate was loaded on 10% SDS–PAGE and transferred on a nitrocellulose membrane. To rule out any gross difference of gel loading, we visually inspected the Ponceau staining. We immunoblotted the membrane with anti-AGT antibody (1:10.000) in 2.5% (w/v) milk in TTBS (50 mM Tris–HCl, pH 7.5, 150 mM NaCl, 0.1% Tween 20) overnight at 4 °C. After three washes in TTBS, the membrane was incubated with peroxidase-conjugated antirabbit immunoglobulin G (IgG) (1:10,000) in 5% milk in TTBS for 1 h at room temperature. We used the anti-GAPDH (1:1000) antibody as loading control. Immunocomplexes were visualized by an enhanced chemiluminescence kit (ECL, Pierce Biotechnology, Rockford, IL).

### Cytotoxicity Assay

CHO-GO-AGTwt and CHO-GO-AGT-G41RMa cells were seeded in 96-well plates at a density of 9000/cm^2^ in the presence or in the absence of the tested compounds. After 7 days, the cytotoxic effect of each inhibitor was evaluated using crystal violet staining as previously reported.^60^

## Supporting Information Available

Spectral changes of AGT wt and the G41R variant in the presence of compound 1 and analogues (Figure S1); effect of compound **1** and analogues on AGTwt and the G41R variant expressed in mammalian cells in the presence of PN (Figure S2); binding studies of synthetized compounds on AGT and G41R variant in the purified form (Figure S3); effects of synthetic compound treatment on AGTwt and the G41R variant expressed in mammalian cells in the presence of PN (Figure S4); cytotoxic effect of selected hit compounds (Figure S5); ranking of five best synthetized compounds (Table S1); and LC–MS analytical method for assessing purity of key target compounds (Annex) (PDF)
Molecular formula strings (CSV)
Compound **1** + AGT (PDB)

## Supplementary Material

jm2c00142_si_002.csv

jm2c00142_si_003.pdb

## Abbreviations Used

AGT: alanine–glyoxylate aminotransferase
AOA: aminooxyacetic acid
CHO: Chinese hamster ovary
GO: glycolate oxidase
PLP: pyridoxal 5′-phosphate
PN: pyridoxine.
