# Supplementary material for: Identification of Human Alanine–Glyoxylate Aminotransferase Ligands as Pharmacological Chaperones for Variants Associated with Primary Hyperoxaluria Type 1
Source: J Med Chem. 2022 Jul 13;65(14):9718–34. doi: 10.1021/acs.jmedchem.2c00142 (PMC9340776; doi:10.1021/acs.jmedchem.2c00142)
Supplement: Supplementary file 1 — jm2c00142_si_001.pdf [file jm2c00142_si_001.pdf]

SUPPLEMENTARY INFORMATION

IDENTIFICATION OF HUMAN ALANINE: GLYOXYLATE AMINOTRANSFERASE LIGANDS AS PHARMACOLOGICAL CHAPERONES FOR VARIANTS ASSOCIATED WITH PRIMARY HYPEROXALURIA TYPE 1.

Silvia Grottelli[1a], Giannamaria Annunziato[2a*], Gioena Pampalone[1a], Marco Pieroni[2], Mirco Dindo[1], Francesca Ferlenghi[2], Gabriele Costantino[2], and Barbara Cellini[1*]

[1]Department of Medicine and Surgery, University of Perugia, P.le L. Severi 1, 06132 Perugia, Italy

[2] P4T group, Department of Food and Drug, University of Parma, Parco Area delle Scienze 27/A 43124, Parma, Italy

[a]The authors contributed equally to this work

Correspondence should be addressed to:

*Prof. Barbara Cellini, Department of Medicine and Surgery, University of Perugia, P.le Severi 1, Perugia, Italy, email: barbara.cellini@unipg.it

*Dr. Giannamaria Annunziato, Department of Food and Drug, University of Parma, Parma, Italy, email: giannamaria.annunziato@unipr.it

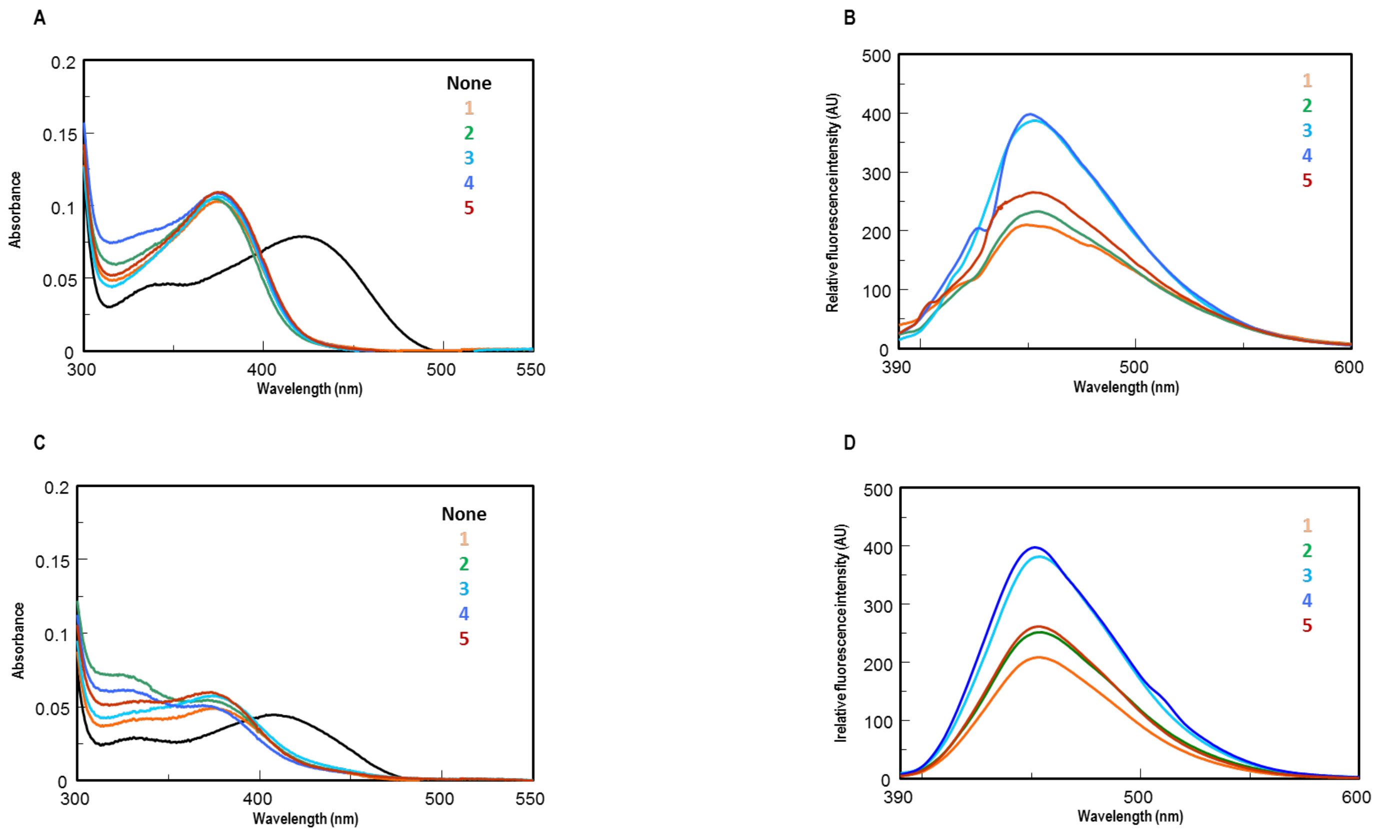

Figure S1. Spectral changes of AGTwt and the G41R variant in the presence of compound 1 and analogues. A) Absorbance and B) external aldimine emission fluorescence (excitation at 370 nm) spectra of 7 μM AGTwt in 0.1 M KP, pH 7.4 in the presence of the indicated compounds at 20 μM concentration. C) Absorbance and D) external aldimine emission fluorescence (excitation at 370 nm) spectra of 7 μM G41R variant in 0.1 M KP, pH 7.4 in the presence of the indicated compounds at 20 μM concentration.

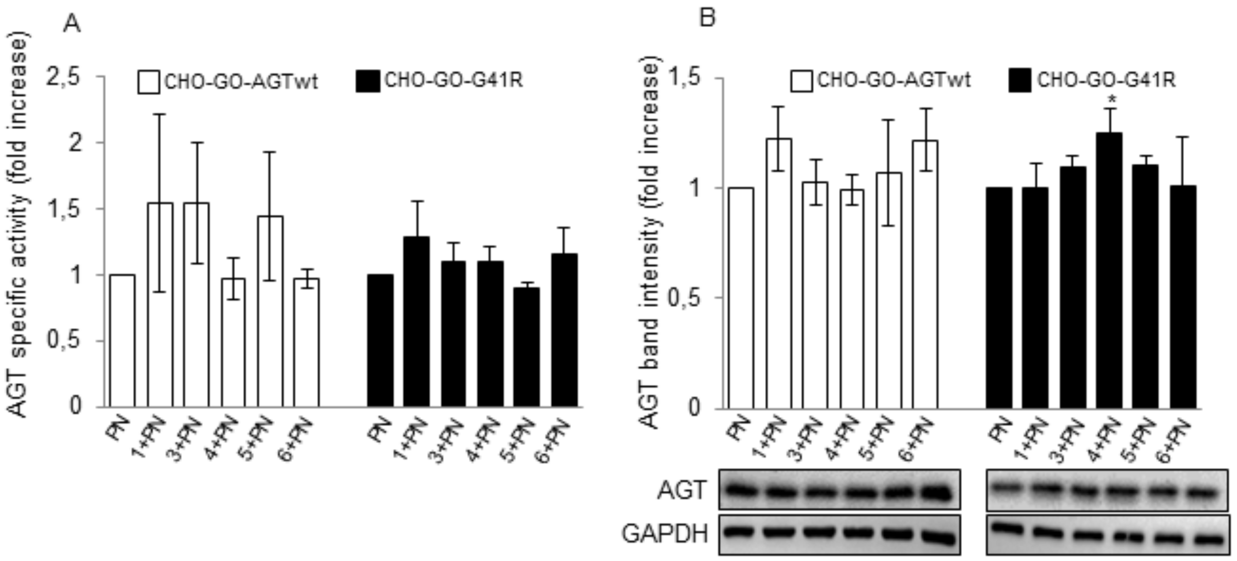

Figure S2. Effect of compound 1 and analogues on AGTwt and the G41R variant expressed in mammalian cells in the presence of PN. CHO-GO-AGTwt and CHO-GO-AGT-G41R cells were grown for seven days in the presence of 50 μM of compound 1 or its analogues and 10 μM PN. At the end of treatment cells were detached, lysed and soluble fraction of each sample used for: (A) transaminase activity determination. Specific activity of AGT in PN-treated-CHO-GO-AGTwt cells (92±11 nmoles of pyruvate/min/mg protein) and in PN-treated-CHO-GO-AGT-G41R cells (114±19 nmoles of pyruvate/min/mg protein) was assumed as 1 to help assess the changes. Data represent mean ± SEM (n=7). *$p<0.05$ vs respective PN-treated cells; (B) AGT protein level quantification by western-blot. AGT levels in in PN-treated-CHO-GO-AGTwt and PN-treated-CHO-GO-AGT-G41R cells was assumed as 1 to help assess the changes. GAPDH has been used as loading control. The images are representative of one out of three separate experiments. Data represent mean ± SEM (n=3). *$p<0.05$vs respective PN-treated cells.

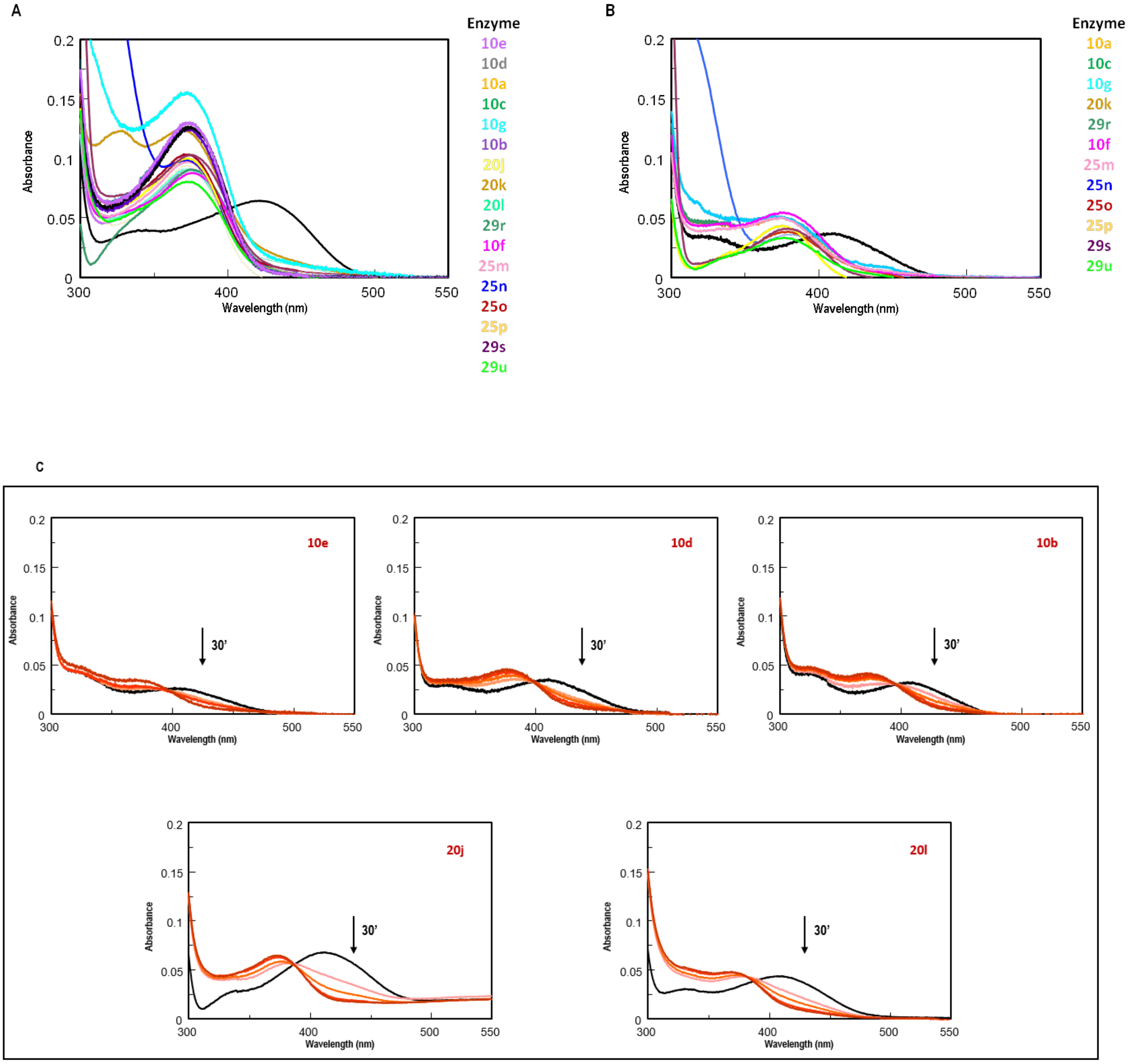

Figure S3. Binding studies of synthetized compounds on AGTwt and G41R variant in the purified form. Spectral changes were evaluated in presence of 5μM enzyme and 100 μM of each compound in 0.1 M KP pH 7.4 at 25°C. A) Spectral changes of AGT-wt in the presence of the indicated compound. B) Spectral changes of the G41R variant in the presence of the indicated compounds. C) Time-dependent spectral changes of the G41R variant in the absence (black) or presence (nuances from pink to red as a function of incubation time as indicated by the arrow) of the indicated compounds.

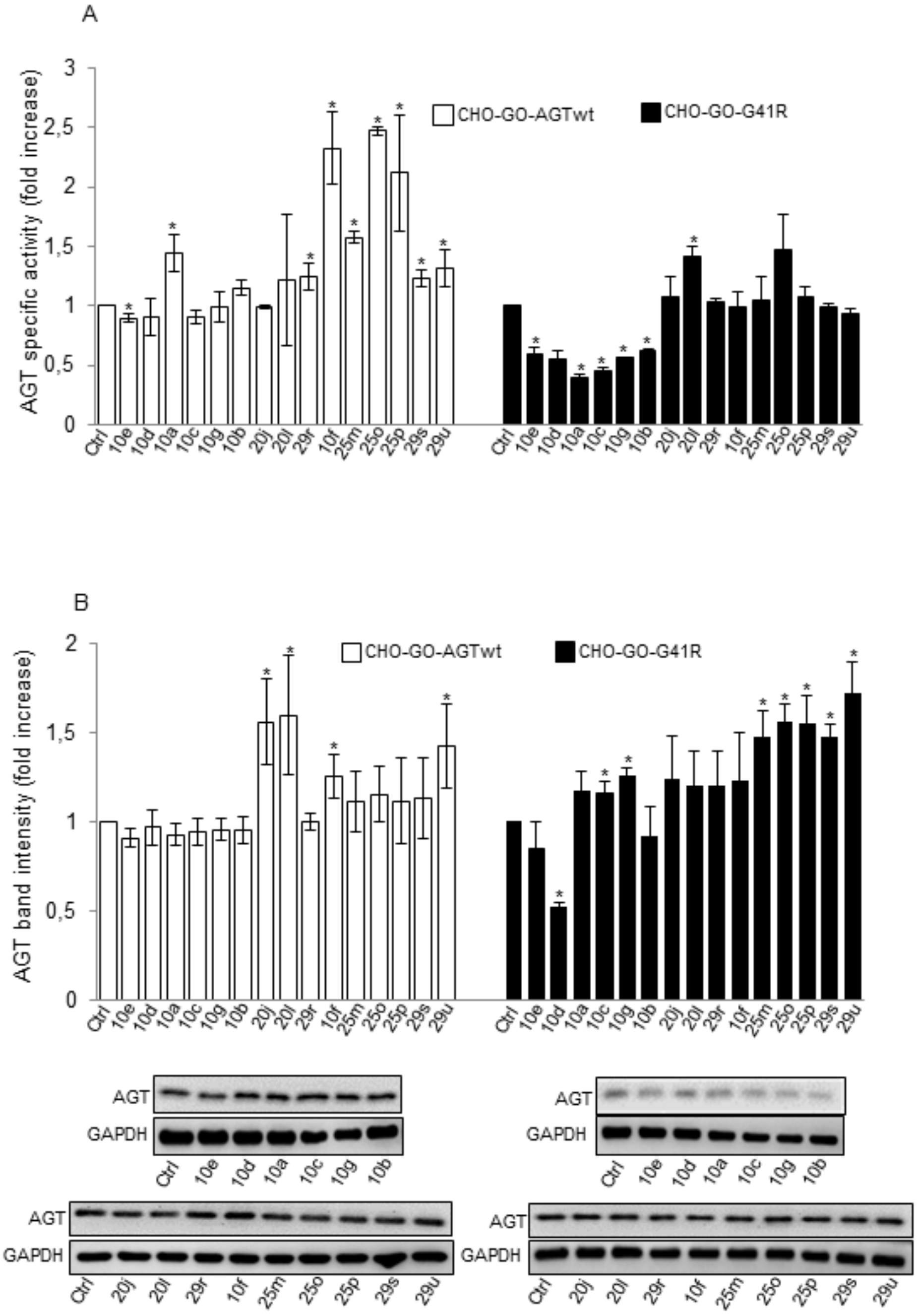

Figure S4. Effects of synthetic compounds treatment on AGTwt and the G41R variant expressed in mammalian cells in the presence of PN. CHO-GO-AGTwt and CHO-GO-AGT-G41R cells were grown for seven days in the presence of 10 μM PN and 50 μM of each compound, as indicated. At the end of treatment cells were detached, lysed and soluble fraction of each sample used for: (A) AGT enzymatic activity determination. Specific activity of AGT in PN-treated-CHO-GO-AGTwt cells (92 ± 11 nmoles of pyruvate/min/mg protein) and in PN-treated-CHO-GO-AGT-G41R cells (114 ± 19 nmoles of pyruvate/min/mg protein) was assumed as 1 to help assess the changes. Data represent mean ± SEM (n=7). $^{*}p<0.05$ vs respective PN-treated cells; (B) AGT expression level quantification. AGT levels in in PN-treated-CHO-GO-AGTwt and PN-treated-CHO-GO-AGT-G41R cells was assumed as 1 to help assess the changes. GAPDH has been used as loading control. The images are representative of one out of three separate experiments. Data represent mean ± SEM (n=4). $^{*}p<0.05$vs respective PN-treated cells.

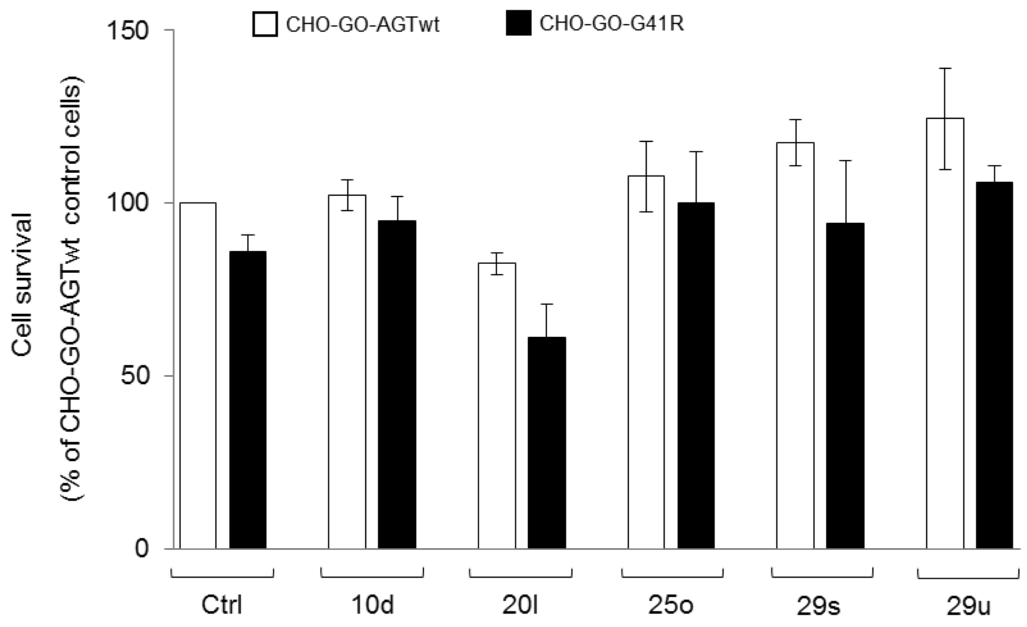

Figure S5. Cytotoxic effect of selected hit compounds. CHO-GO-AGTwt and CHO-GO-G41R cells were seeded in 96-well plates in Ham's F12 Glutamax medium in the absence or in the presence of the indicated compounds at 50μM concentration. After 7 days, cell viability was assessed by crystal violet staining and results expressed as percentage respect to CHO-GO-AGTwt cells.

Table S1. Ranking of 5 best synthetized compounds. Fold increase values of specific activity and protein expression level for CHO-GO-AGTwt and CHO-GO-G41R cells treated with 5 best hit compounds ranked.

	Wild-type AGT				PH1-associated G41R variant			
	Specific activity Fold increase		Protein expression level Fold increase		Specific activity Fold increase		Protein expression level Fold increase	
Compound	vs AGTwt control cells	vs AGTwt PN treated cells	vs AGTwt control cells	vs AGTwt PN treated cells	vs G41R-MA control cells	vs G41R-MA PN treated cells	vs G41R-MA control cells	vs G41R-MA control cells
**25o**	1.55 ± 0.09	2.5 ± 0.03	1.09 ± 0.005	1.15 ± 0.16	1.95 ± 0.03	1.47 ± 0.3	1.35 ± 0.03	1.56 ± 0.1
**20l**	1.18 ± 0.08	1.21 ± 0.55	0.99 ± 0.08	1.59 ± 0.33	1.42 ± 0.07	1.41 ± 0.08	1.08 ± 0.04	1.2 ± 0.19
**29u**	0.85 ± 0.07	1.38 ± 0.15	1.07 ± 0.11	1.42 ± 0.23	1.17 ± 0.1	0.93 ± 0.03	1.46 ± 0.18	1.72 ± 0.17
**29s**	0.84 ± 0.09	1.23 ± 0.07	1.02 ± 0.02	1.13 ± 0.23	1.62 ± 0.12	0.98 ± 0.02	1.19 ± 0.03	1.47 ± 0.07
**10d**	0.48 ± 0.05	0.9 ± 0.16	0.81 ± 0.03	0.96 ± 0.09	1.45 ± 0.03	0.55 ± 0.07	0.99 ± 0.16	0.52 ± 0.02

Annex S1.

LC-MS analytical method for assessing purity of key target compounds.
10 mM stock solutions of test compounds were freshly prepared in DMSO and further diluted to get a 5, 100 or 200 µM standard solution in MeOH, which was directly analyzed by HPLC-MS.
An Accela UHPLC system (Thermo, USA), equipped with a Waters XSelect® HSS T3 column (100 × 2.1 mm i.d., 3.5 µm particle size; Waters, USA) was used for chromatographic separation. Mobile phases were ACN (eluent A) and ultra-pure water (eluent B), both added with formic acid at 0.1% v/v, at a flow rate of 220 µL min^{-1}. Employed gradient conditions were as follows: from 95 to 5%B in 12 min; 5%B from 12 to 16 min and returning to 95%B in 1 min followed by 3-min reconditioning. Total run time: 20 min.
A Thermo TSQ Quantum Access Max triple quadrupole mass spectrometer (Thermo, San Jose, CA, USA) equipped with a heated electrospray ionization (H-ESI) source was employed for compound detection. All analyses were performed by setting ion source voltage at 3000 V, Collision Induced Dissociation (CID) voltage at 12 V and capillary temperature at 270 °C. Nitrogen served both as sheath and auxiliary gas at 35 psi and 15 psi, respectively; argon with a pressure of 1.5 mtorr was employed as collision gas.
Sample concentration and adopted mass range are reported for each compound together with the corresponding chromatograms in Positive (PIM) and Negative Ion Mode (NIM) of (i) the vehicle and (ii) the standard solution of test compound in MeOH and the mass spectrum of the most abundant peak. Xcalibur software version 2.2 (Thermo, USA) was employed for both data acquisition and processing.

Annex S1a. Upper panel. Full Scan HPLC Trace in Positive Ion Mode (PIM, ESI$^+$ mass range 100-600) of vehicle (upper trace) and compound **10f** 5 µM in MeOH+0.05% DMSO (lower trace); **Lower panel**. Traces reported in the upper panel zoomed in the retention time window from 3 to 12 minutes.

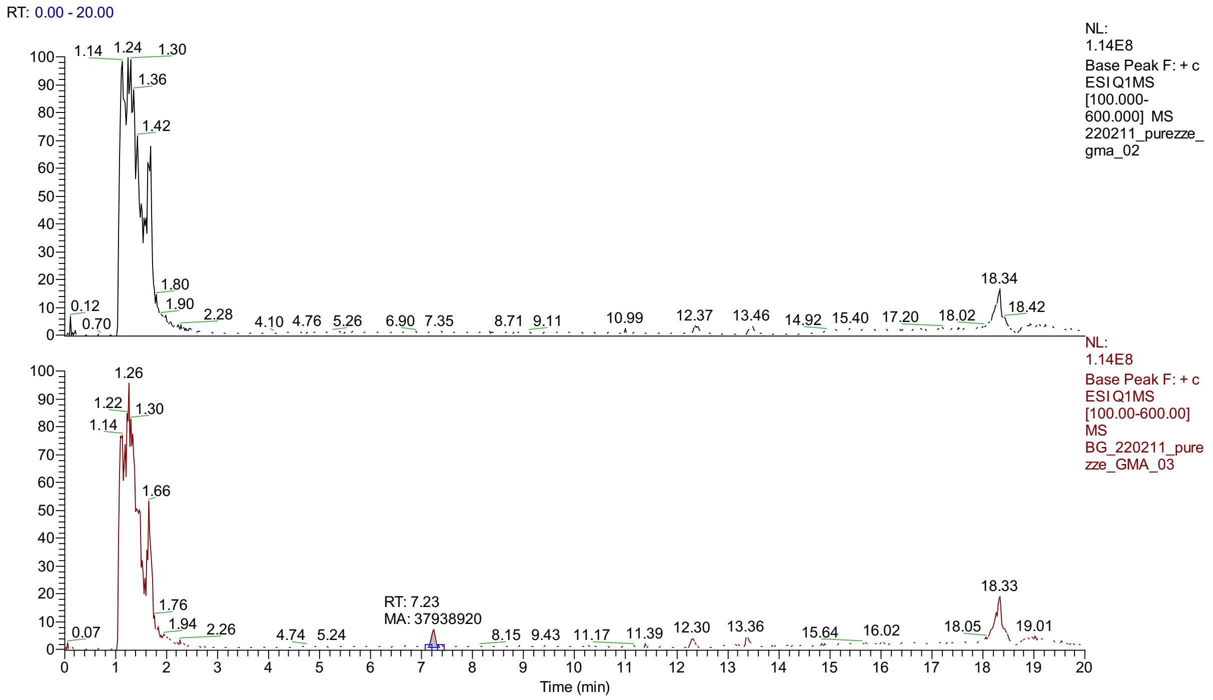

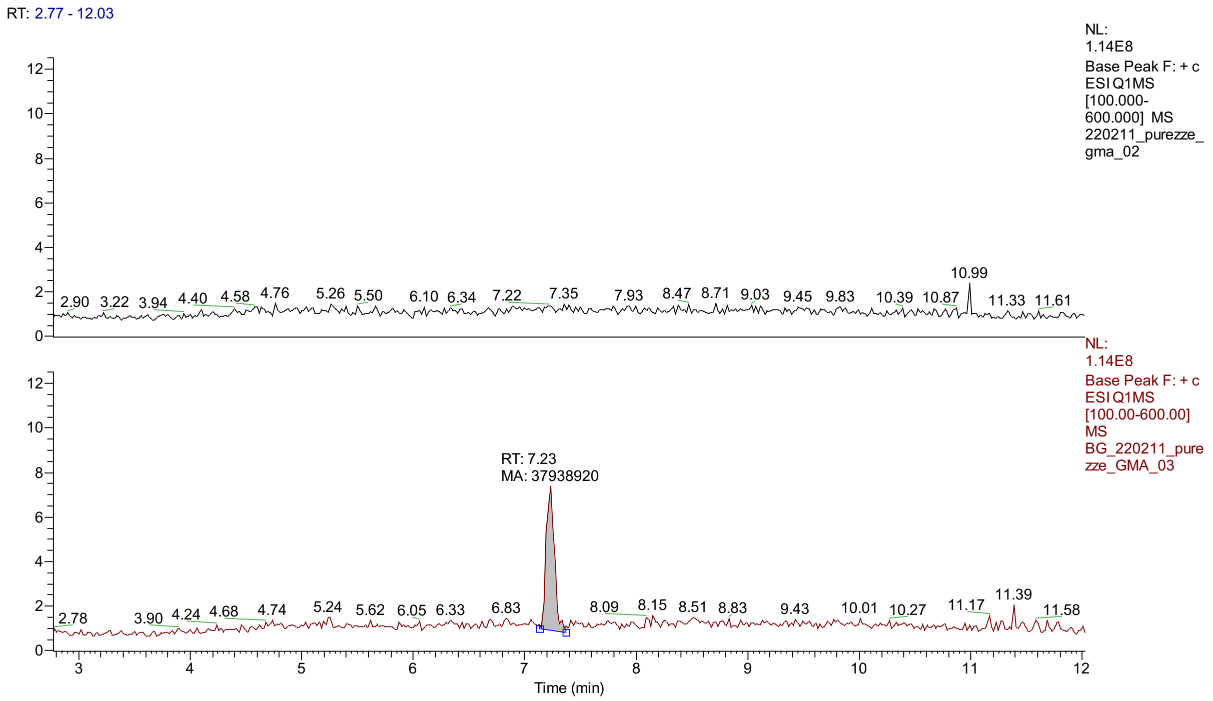

b. MS spectrum of peak at RT=7.23 min.

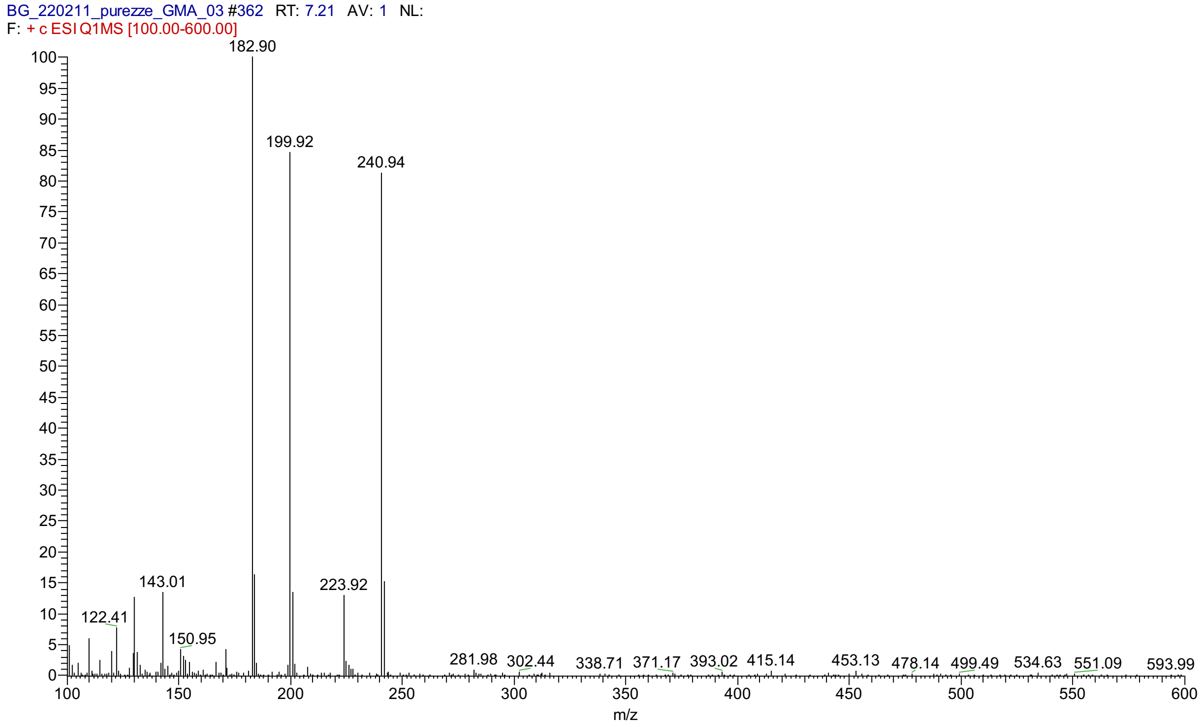

c. Full Scan HPLC Trace in Negative Ion Mode (NIM, ESI¯ mass range 100-600) of vehicle (upper trace) and compound **10f** 5 µM in MeOH+0.05% DMSO (lower trace).

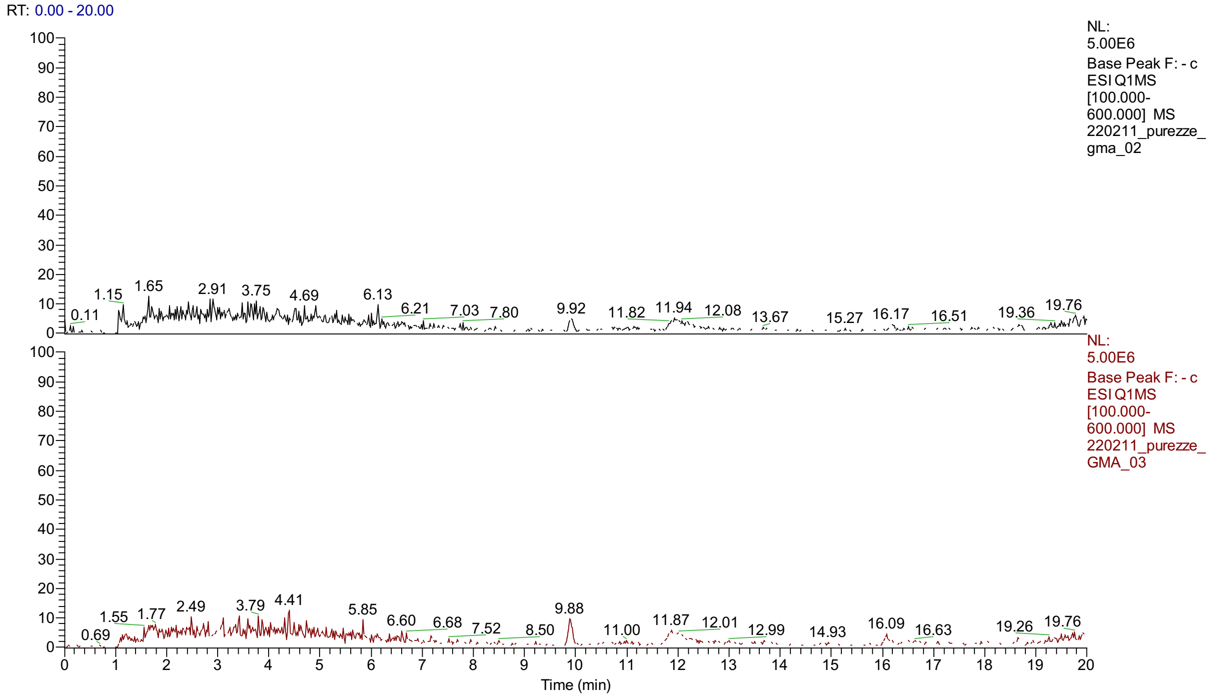

Purity > 99%.

No relevant impurity-related peaks were detected after careful inspection of chromatogram and corresponding full scan MS spectrum in both PIM and NIM.

Annex S2a. Upper panel. Full Scan HPLC Trace in PIM (ESI$^+$ mass range 100-600) of vehicle (upper trace) and compound **25m** 5 µM in MeOH+0.05% DMSO (lower trace); **Lower panel**. Traces reported in the upper panel zoomed in the retention time window from 3 to 11 minutes.

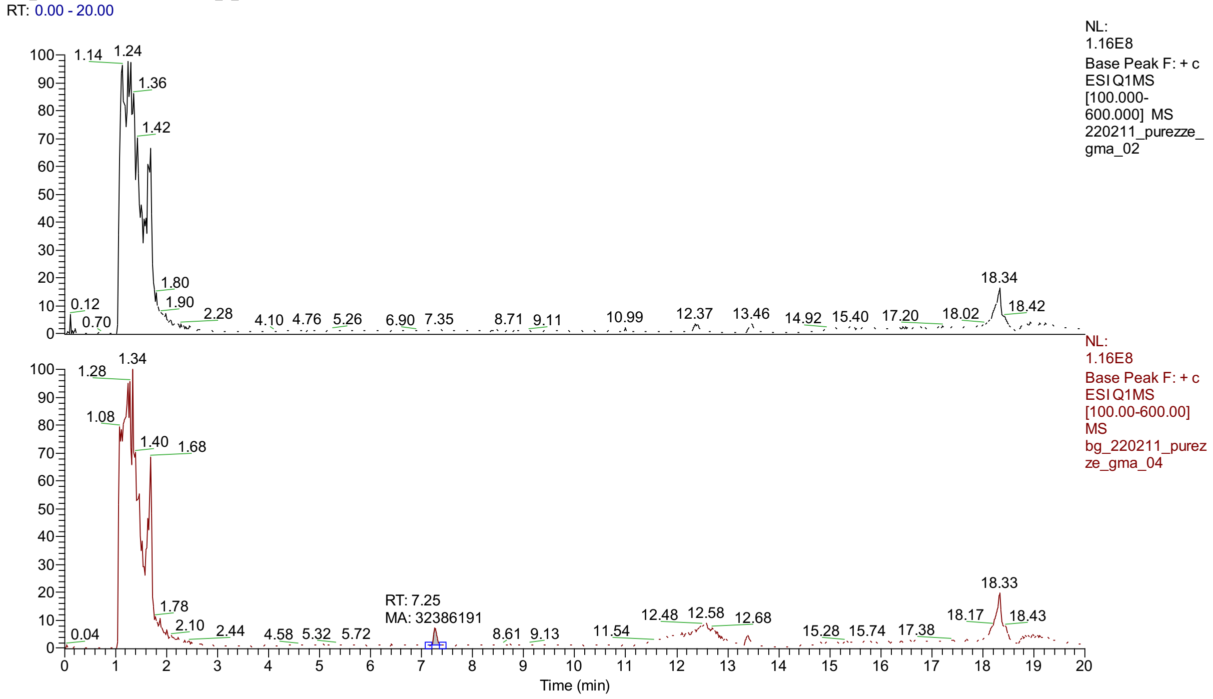

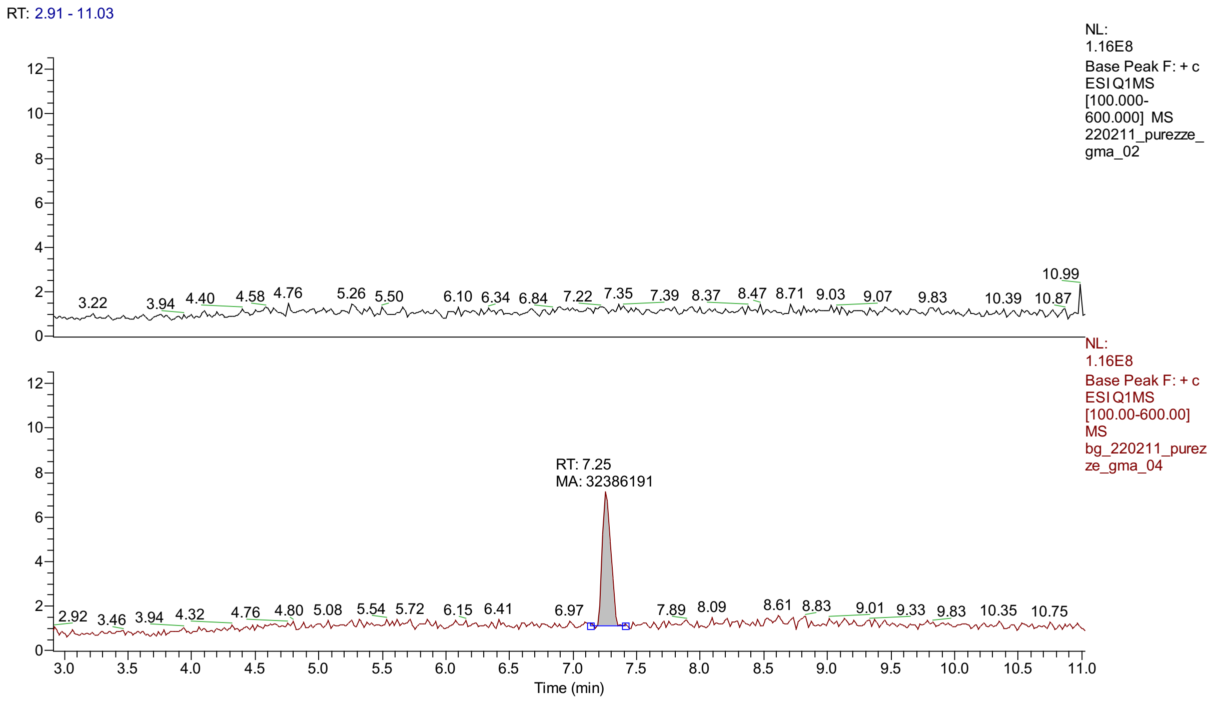

b. MS spectrum of peak at RT=7.25 min.

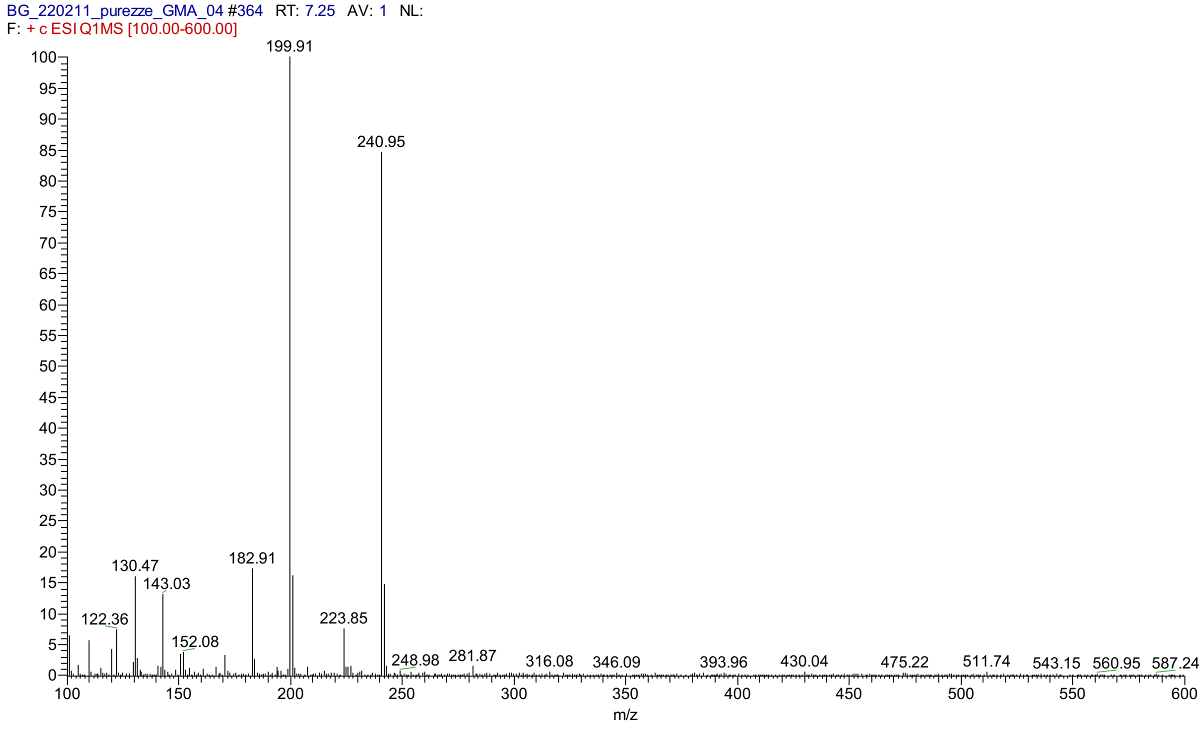

c. Full Scan HPLC Trace in NIM (ESI^{-} mass range 100-600) of vehicle (upper trace) and compound **25m** 5 μM in MeOH+0.05% DMSO (lower trace).

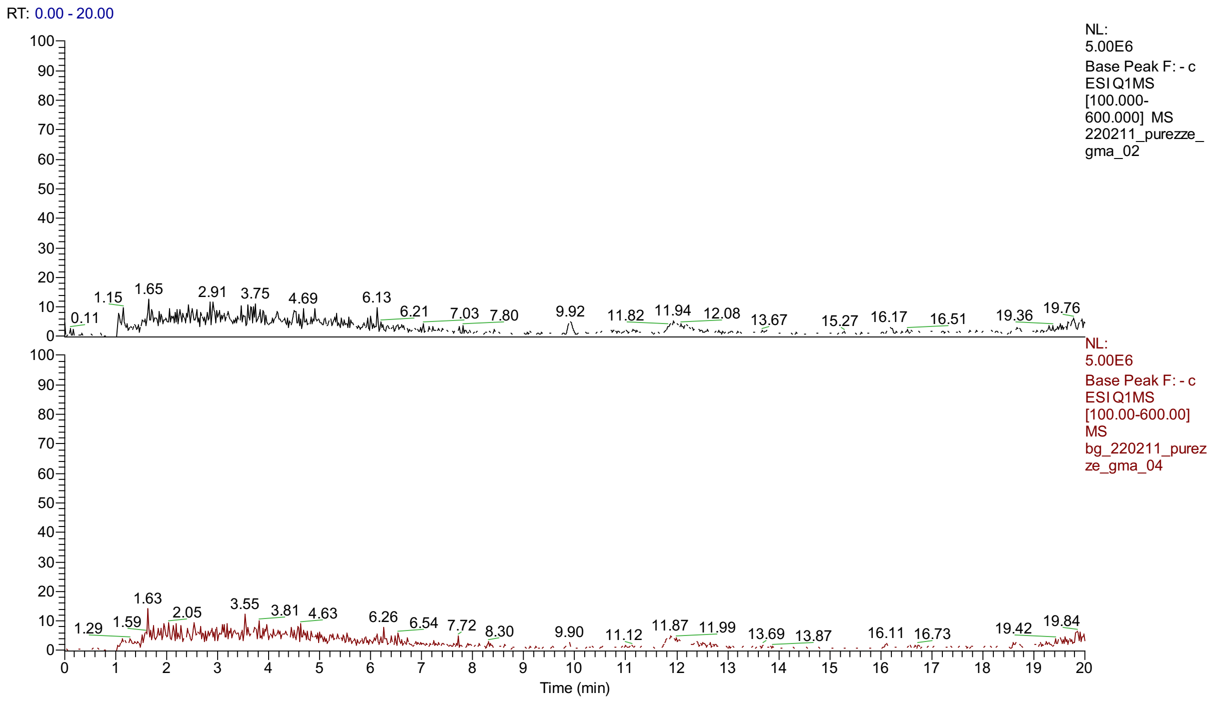

Purity > 99%.
No relevant impurity-related peaks were detected after careful inspection of chromatogram and corresponding full scan MS spectrum in both PIM and NIM.

Annex S3a. Upper panel. Full Scan HPLC Trace in PIM (ESI$^+$ mass range 100-600) of vehicle (upper trace) and compound **25o** 5 µM in MeOH+0.05% DMSO (lower trace); **Lower panel**. Traces reported in the upper panel zoomed in the retention time window from 3 to 12 minutes.

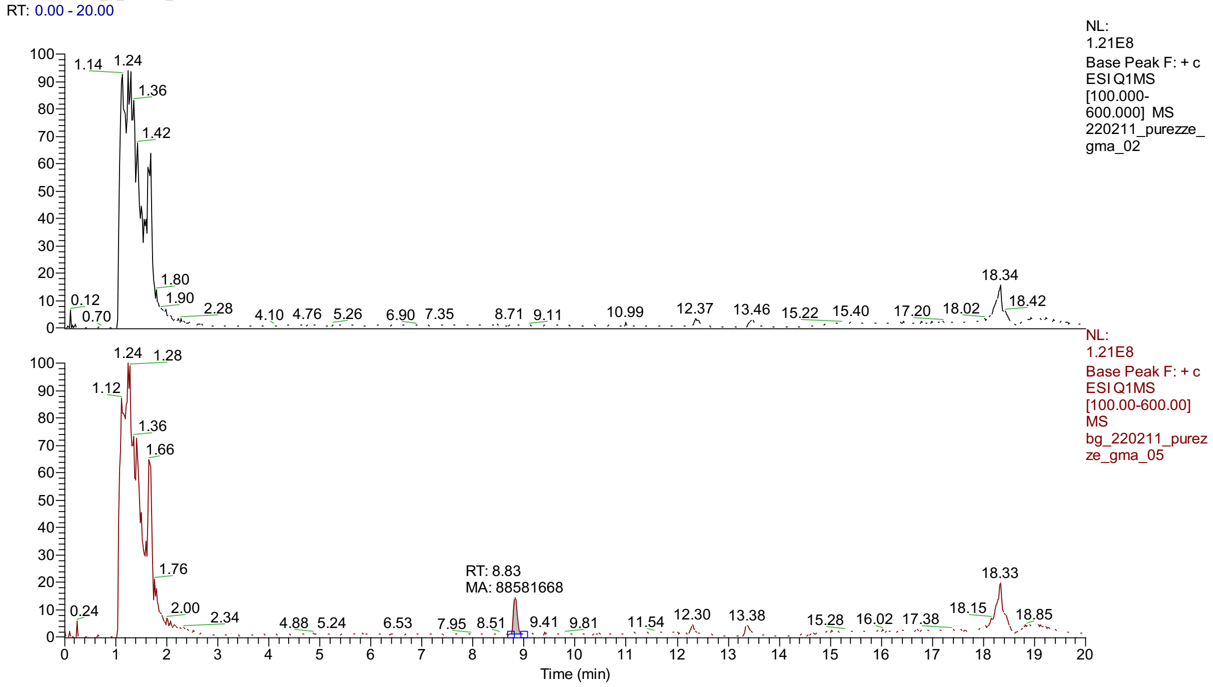

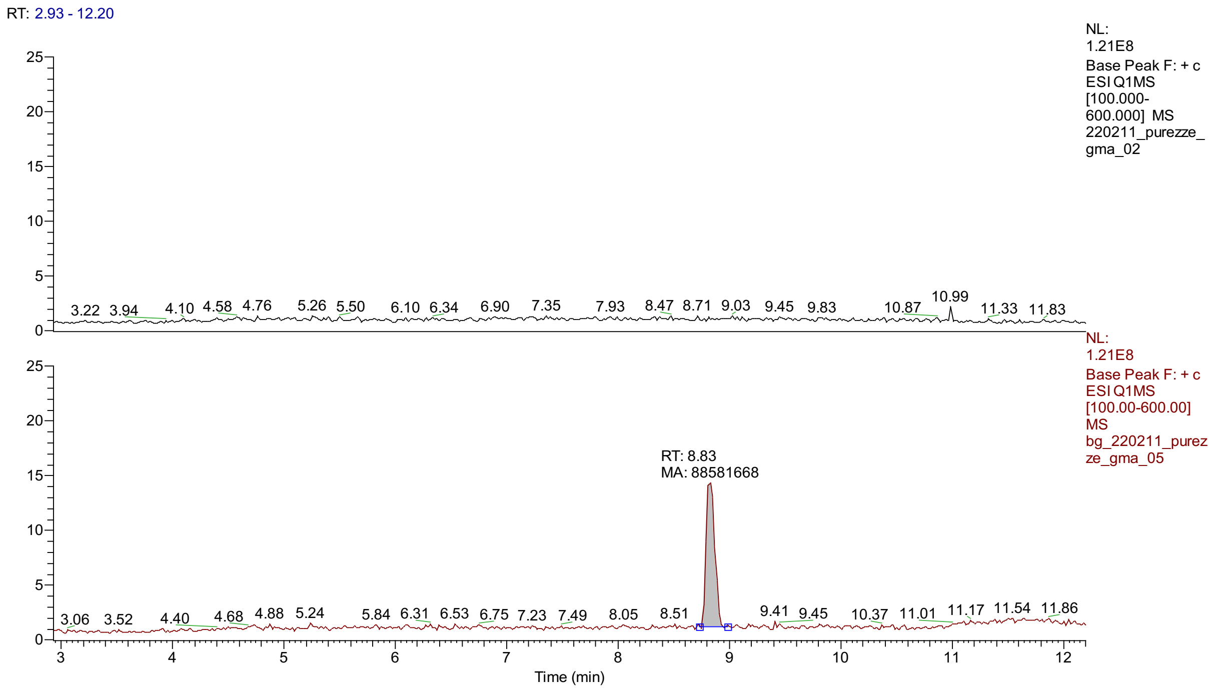

b. MS spectrum of peak at RT=8.83 min.

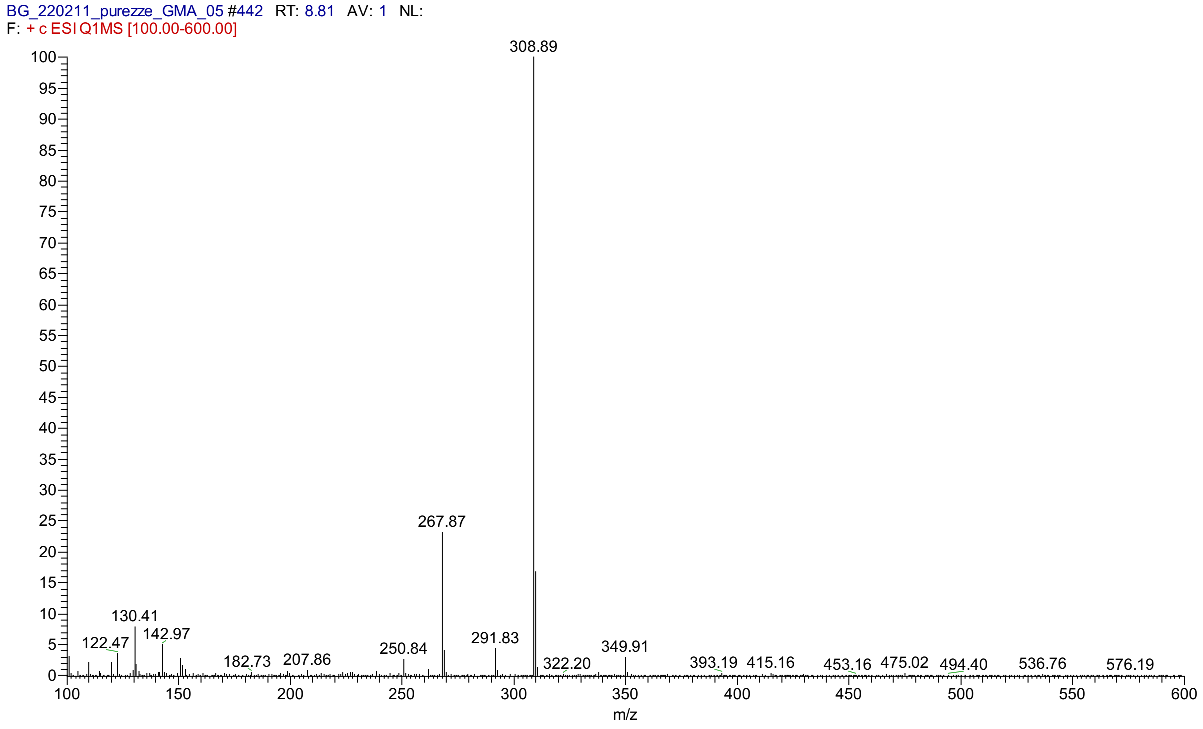

c. Full Scan HPLC Trace in NIM (ESI¯ mass range 100-600) of vehicle (upper trace) and compound **25o** 5 µM in MeOH+0.05% DMSO (lower trace).

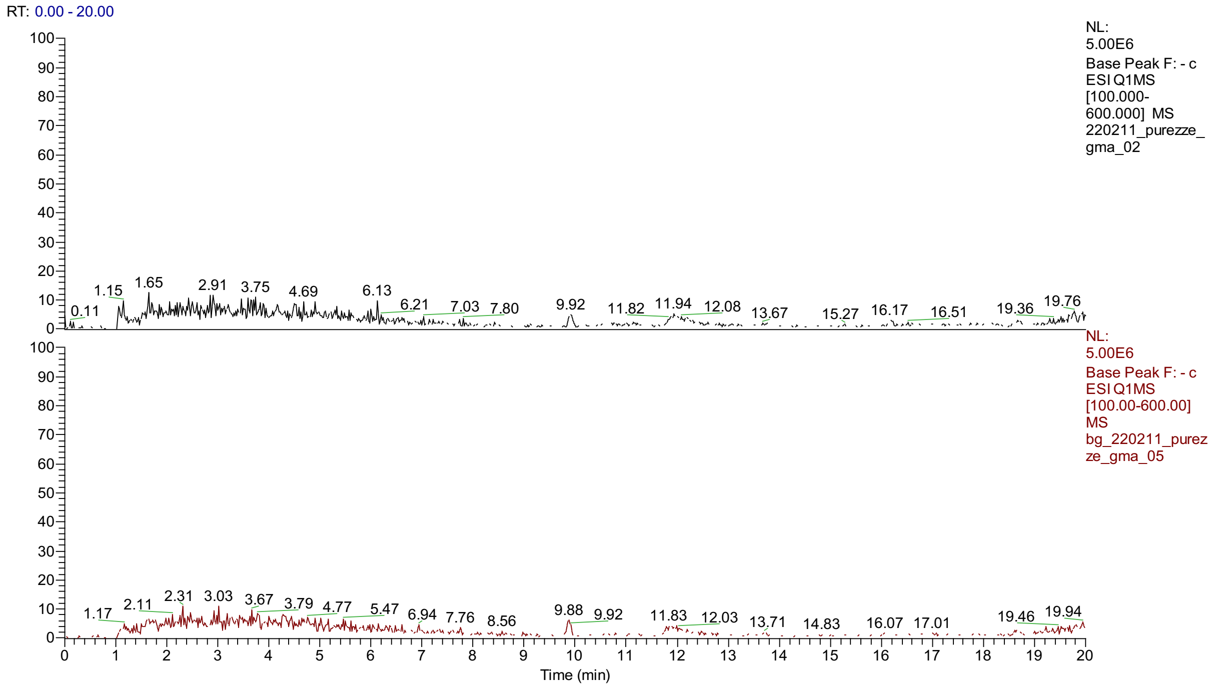

Purity > 99%.

No relevant impurity-related peaks were detected after careful inspection of chromatogram and corresponding full scan MS spectrum in both PIM and NIM.

Annex S4a. Upper panel. Full Scan HPLC Trace in PIM (ESI$^+$ mass range 100-600) of vehicle (upper trace) and compound **25p** 5 µM in MeOH+0.05% DMSO (lower trace); **Lower panel**. Traces reported in the upper panel zoomed in the retention time window from 3 to 12 minutes.

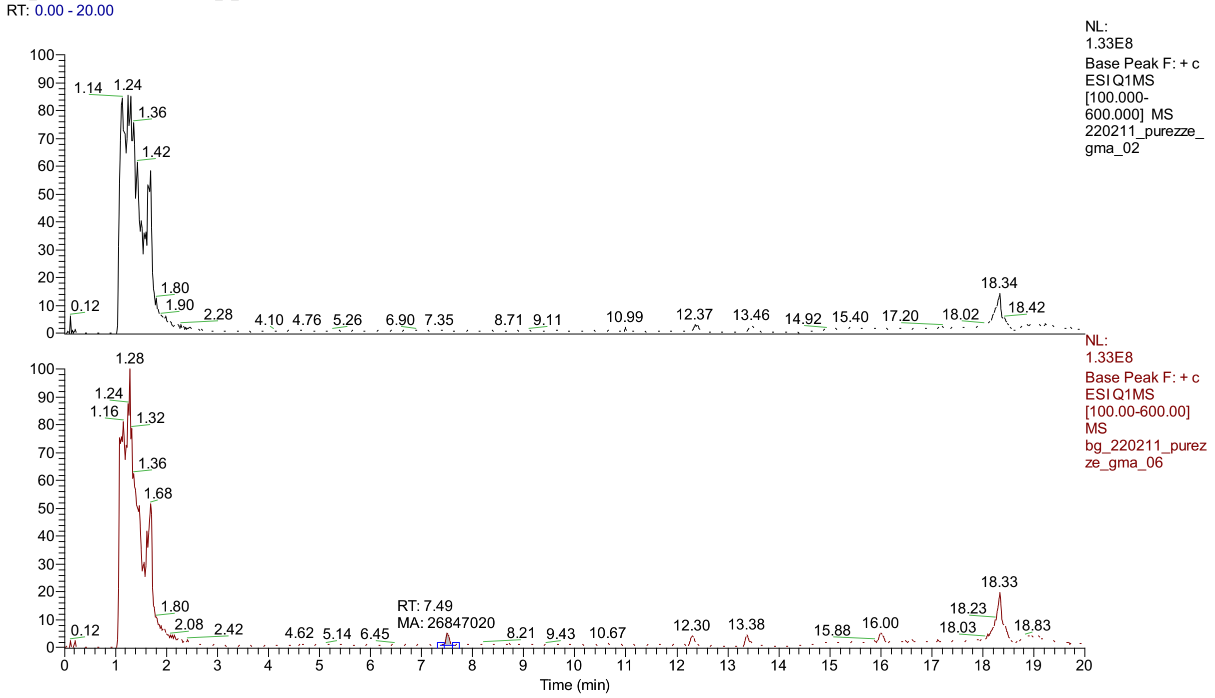

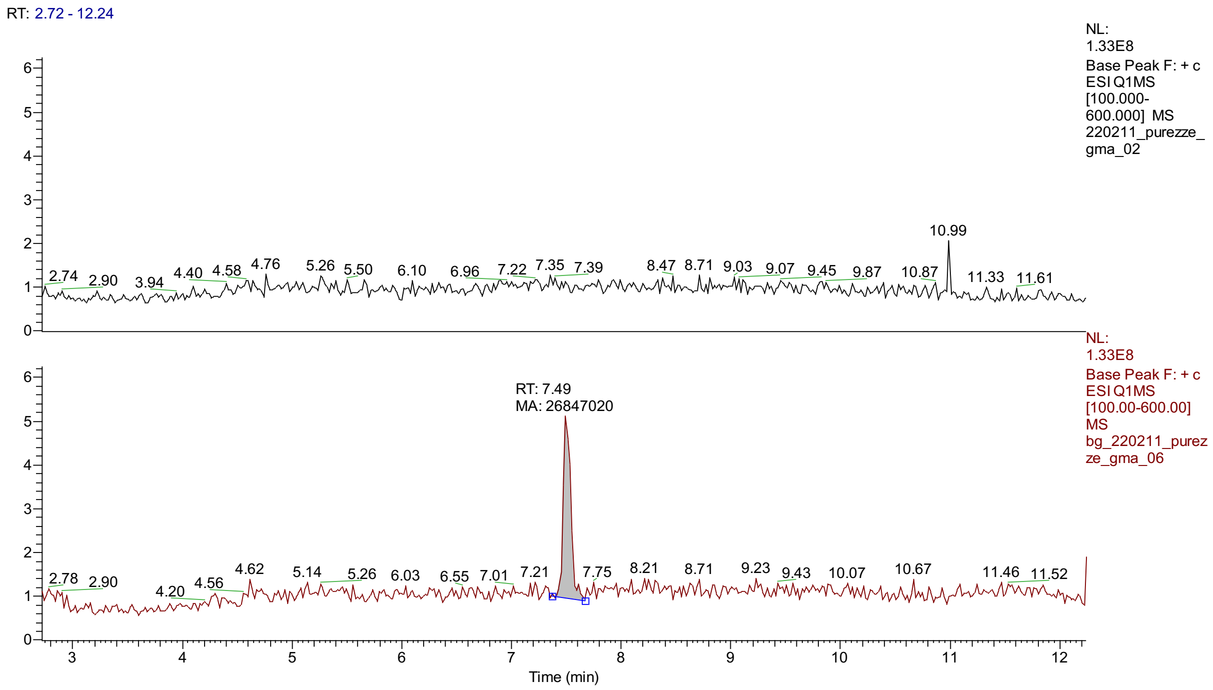

b. MS spectrum of peak at RT=7.49 min.

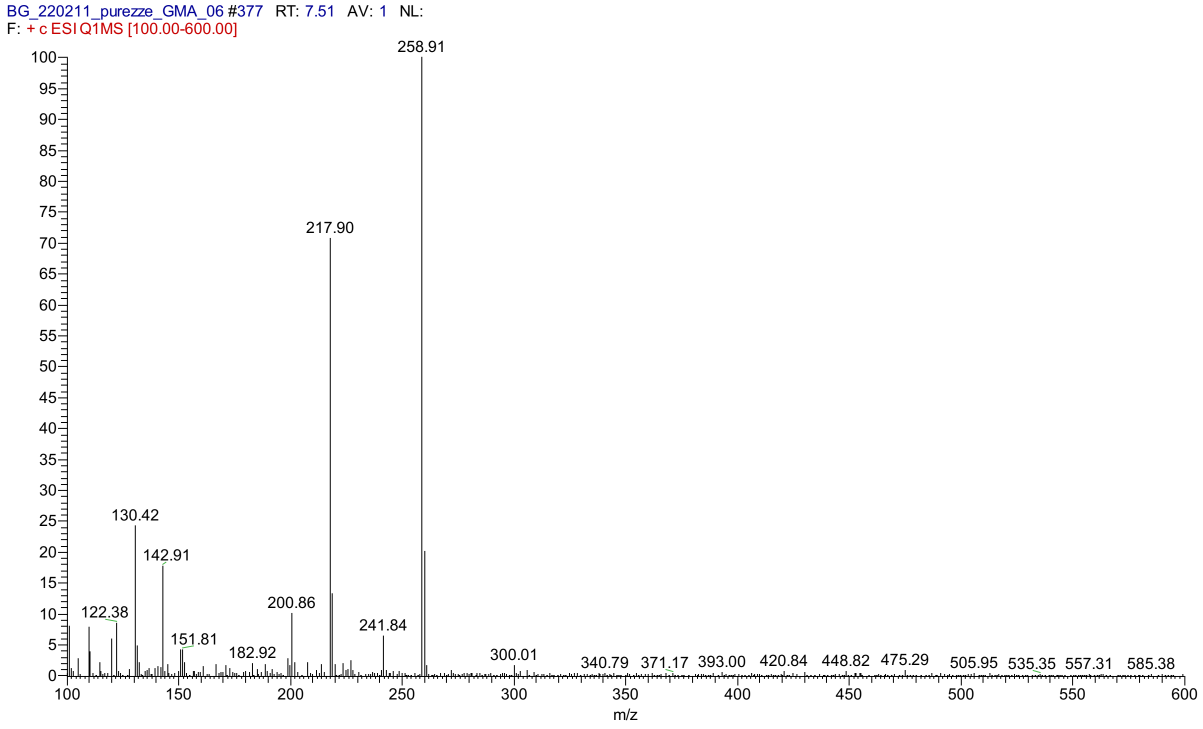

c. Full Scan HPLC Trace in NIM (ESI^{-} mass range 100-600) of vehicle (upper trace) and compound **25p** 5 µM in MeOH+0.05% DMSO (lower trace).

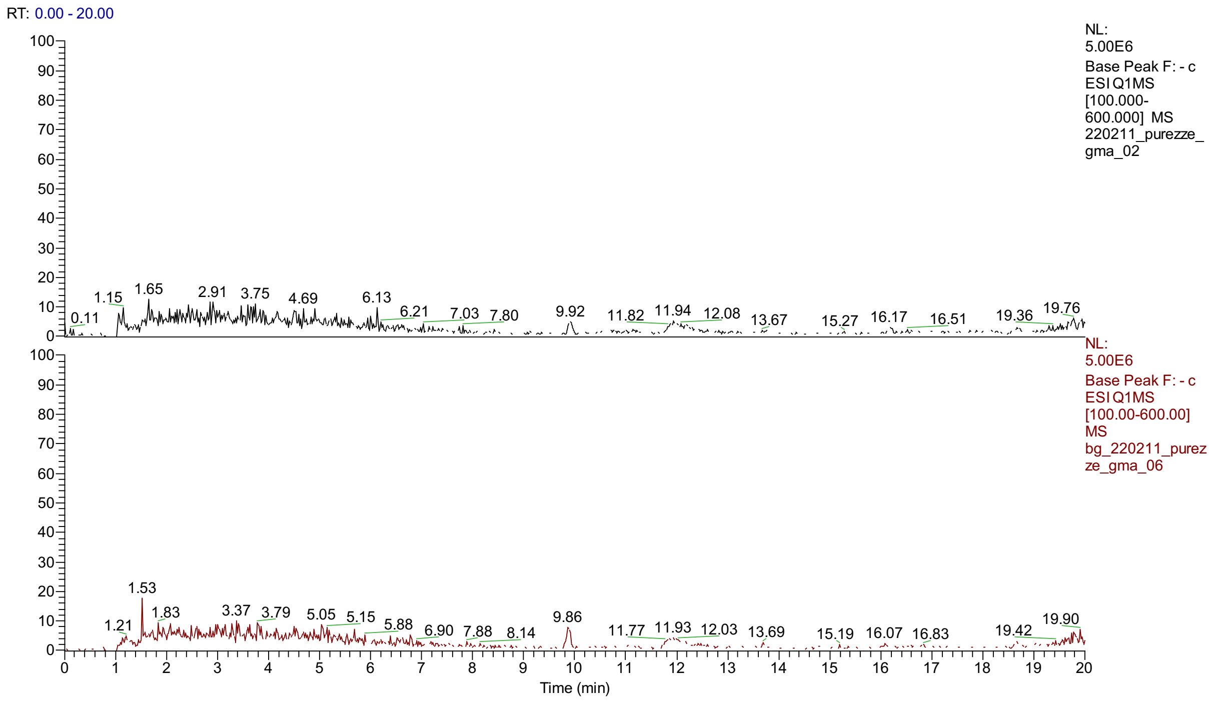

Purity > 99%.
No relevant impurity-related peaks were detected after careful inspection of chromatogram and corresponding full scan MS spectrum in both PIM and NIM.

Annex S5a. Upper panel. Full Scan HPLC Trace in PIM (ESI$^+$ mass range 100-600) of vehicle (upper trace) and compound **29r** 20 µM in MeOH+0.2% DMSO (lower trace); **Lower panel**. Traces reported in the upper panel zoomed in the retention time window from 4 to 11 minutes.

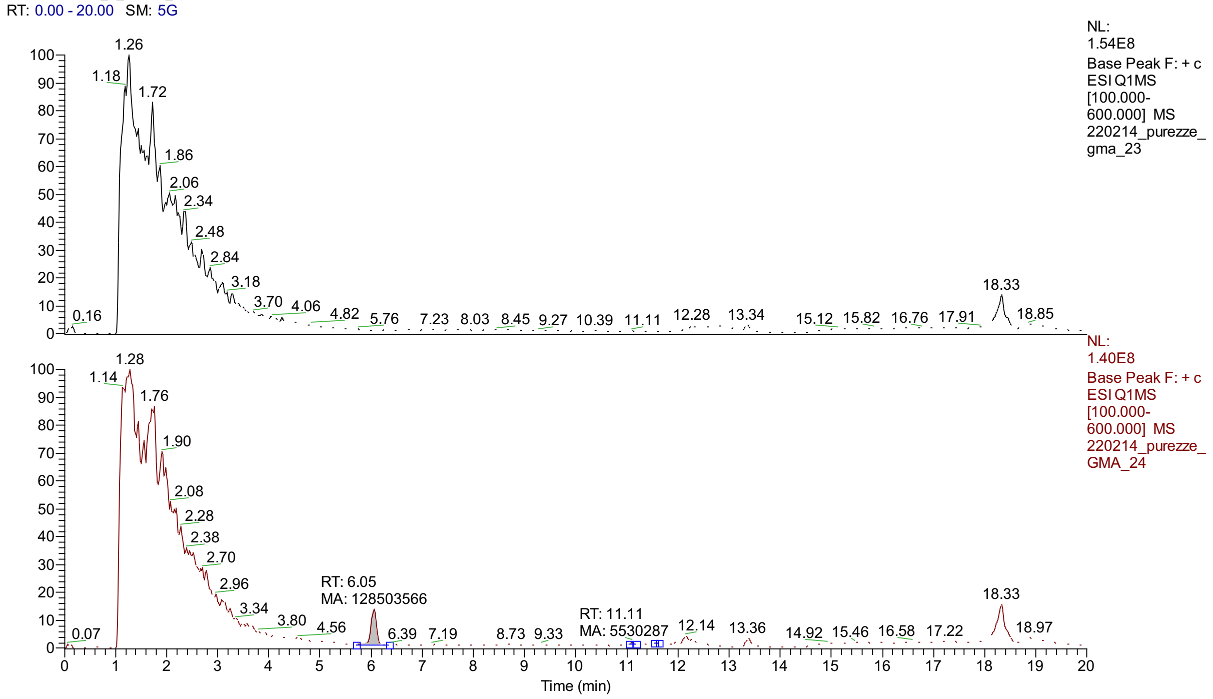

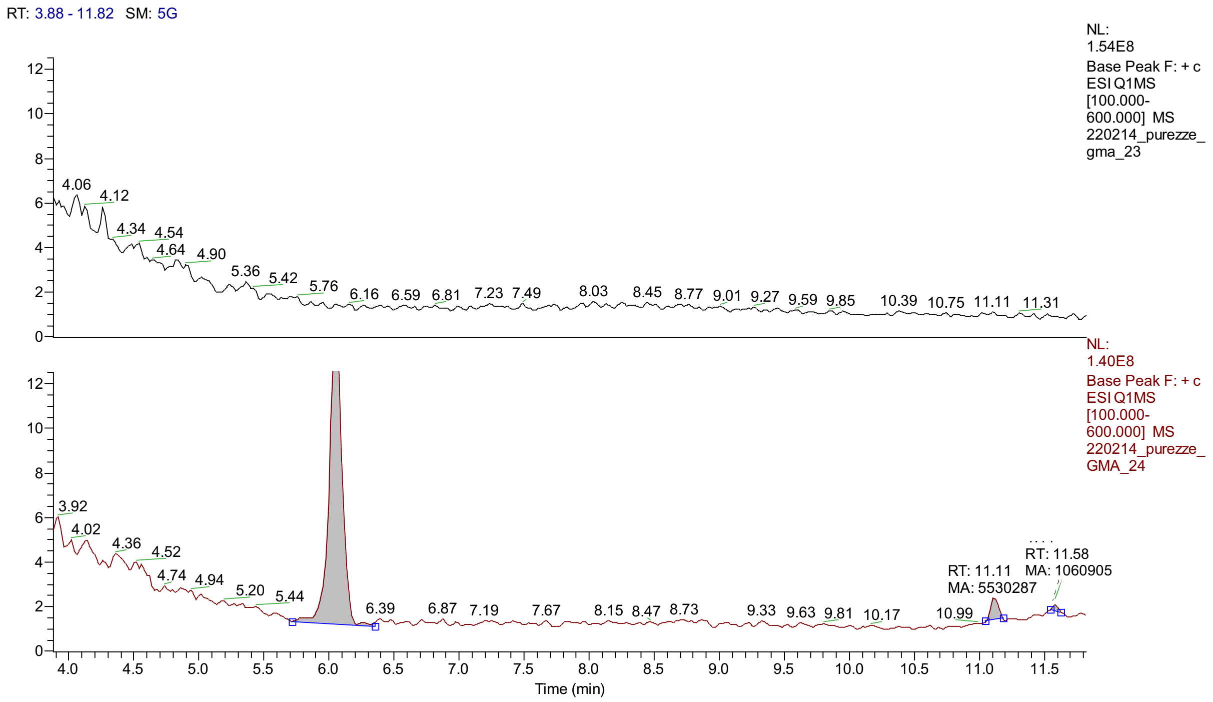

RT (min)	Area	%Area
6.05	128503566	95.12
11.11	5530287	4.09
11.58	1060905	0.8
**SUM**	135094758	100

b. MS spectrum of peak at RT=6.05 min.

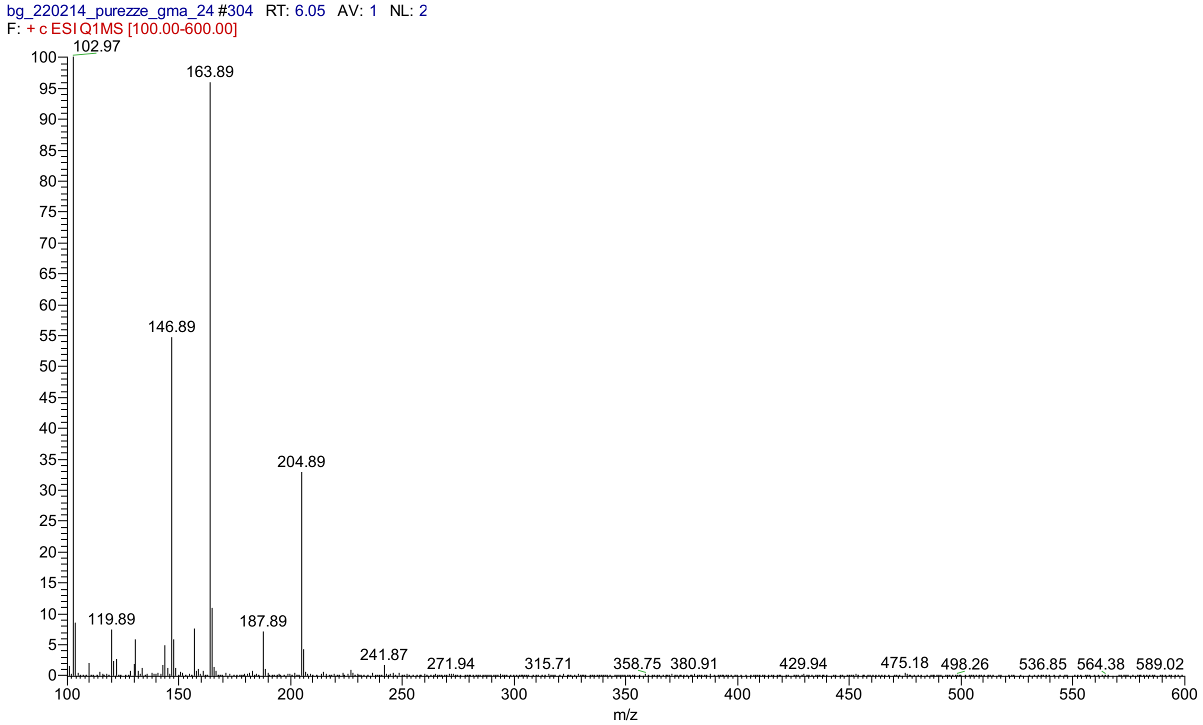

c. Full Scan HPLC Trace in Negative Ion Mode (NIM, ESI¯ mass range 100-600) of vehicle (upper trace) and compound **29r** 20 µM in MeOH+0.2% DMSO (lower trace).

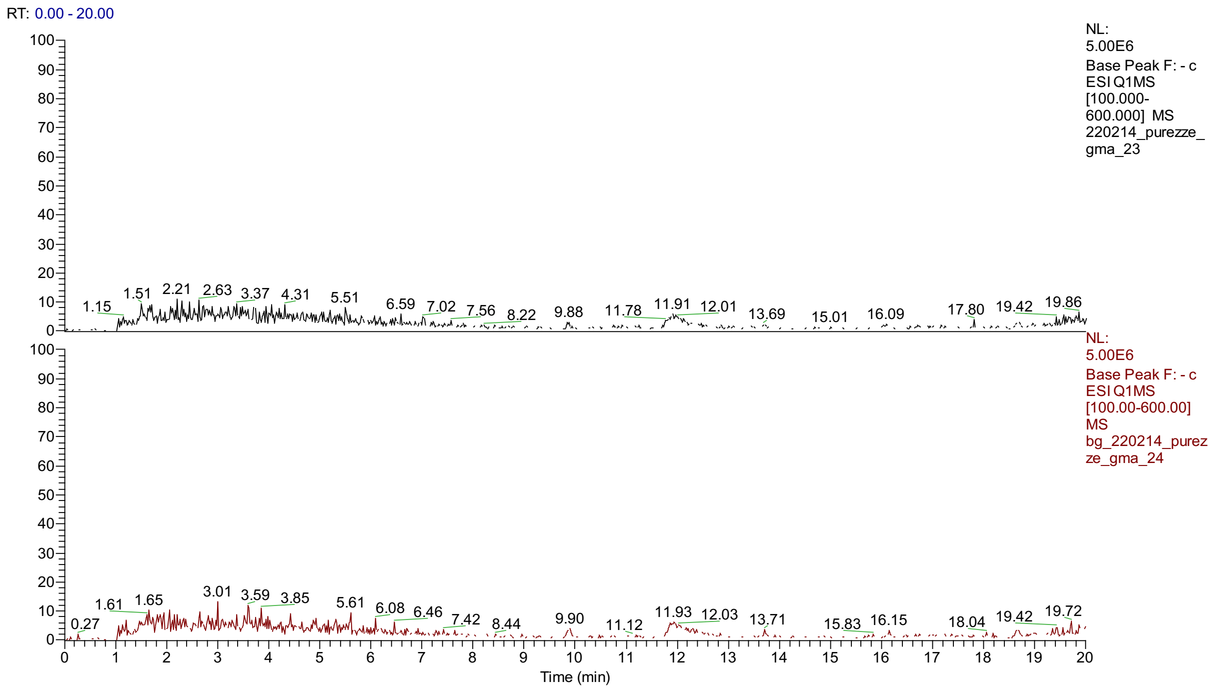

Annex S6a.Upper panel. Full Scan HPLC Trace in PIM (ESI$^+$ mass range 50-500) of vehicle (upper trace) and compound **10d** 100 µM in MeOH+1% DMSO (lower trace); **Lower panel**. Traces reported in the upper panel zoomed in the retention time window from 3 to 17 minutes.

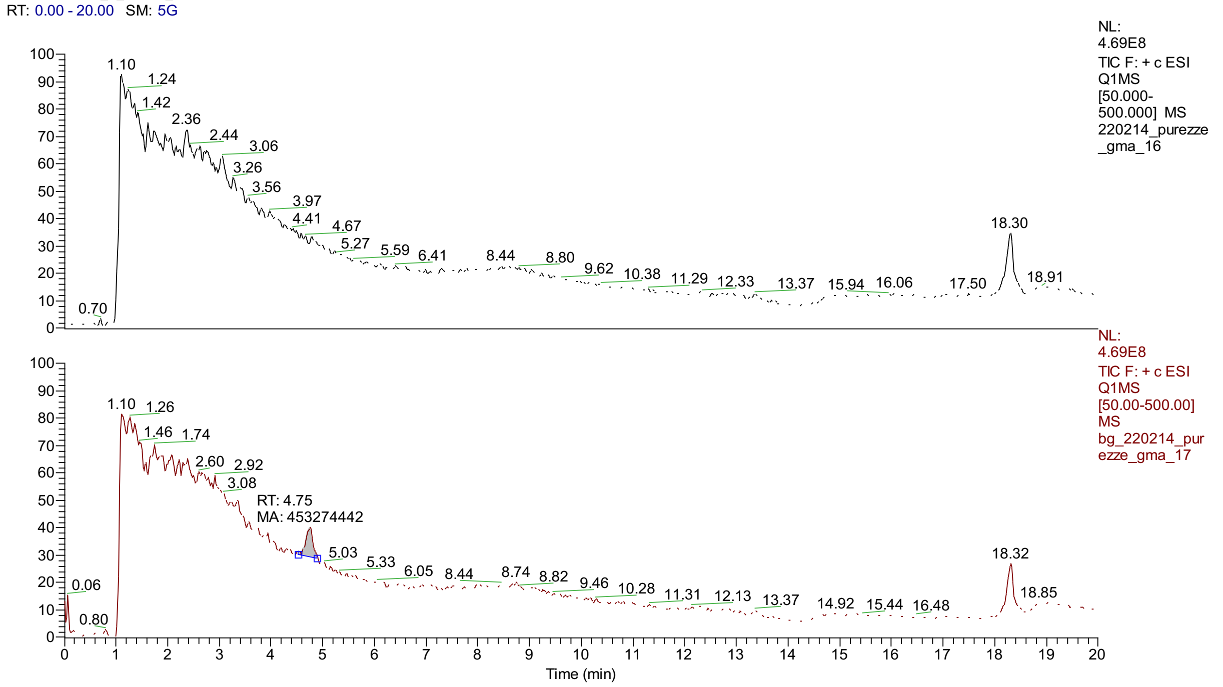

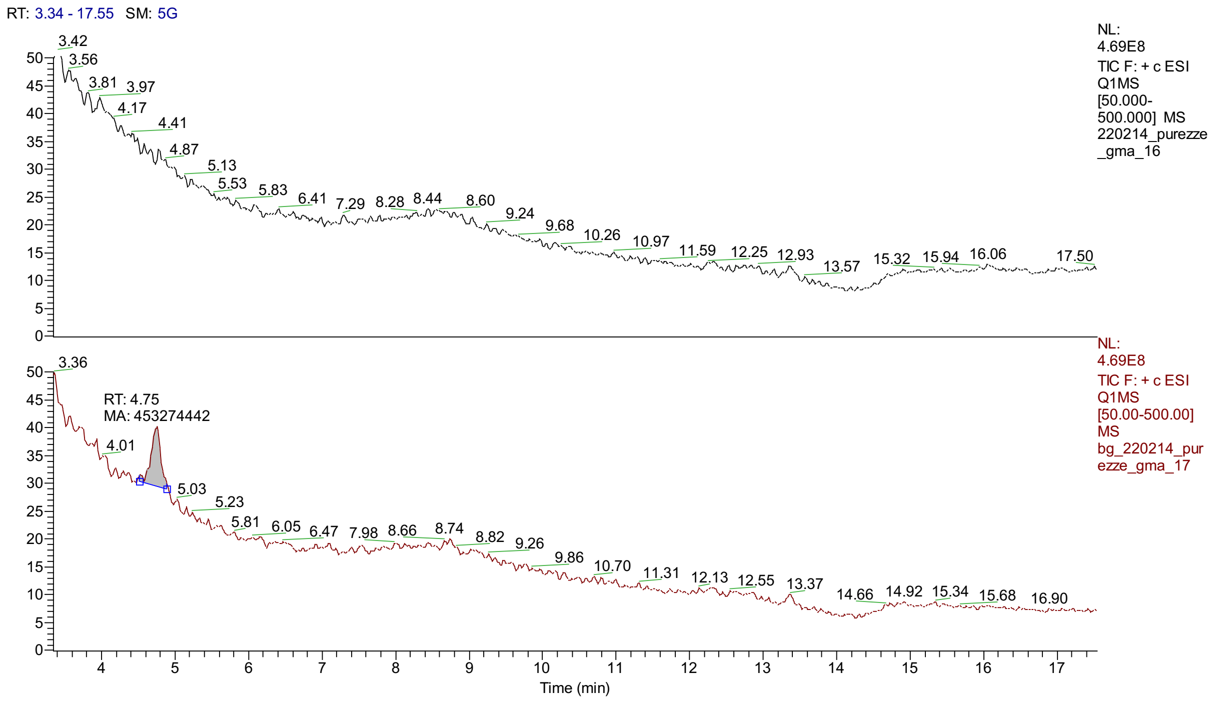

b. MS spectrum of peak at RT=4.75 min.

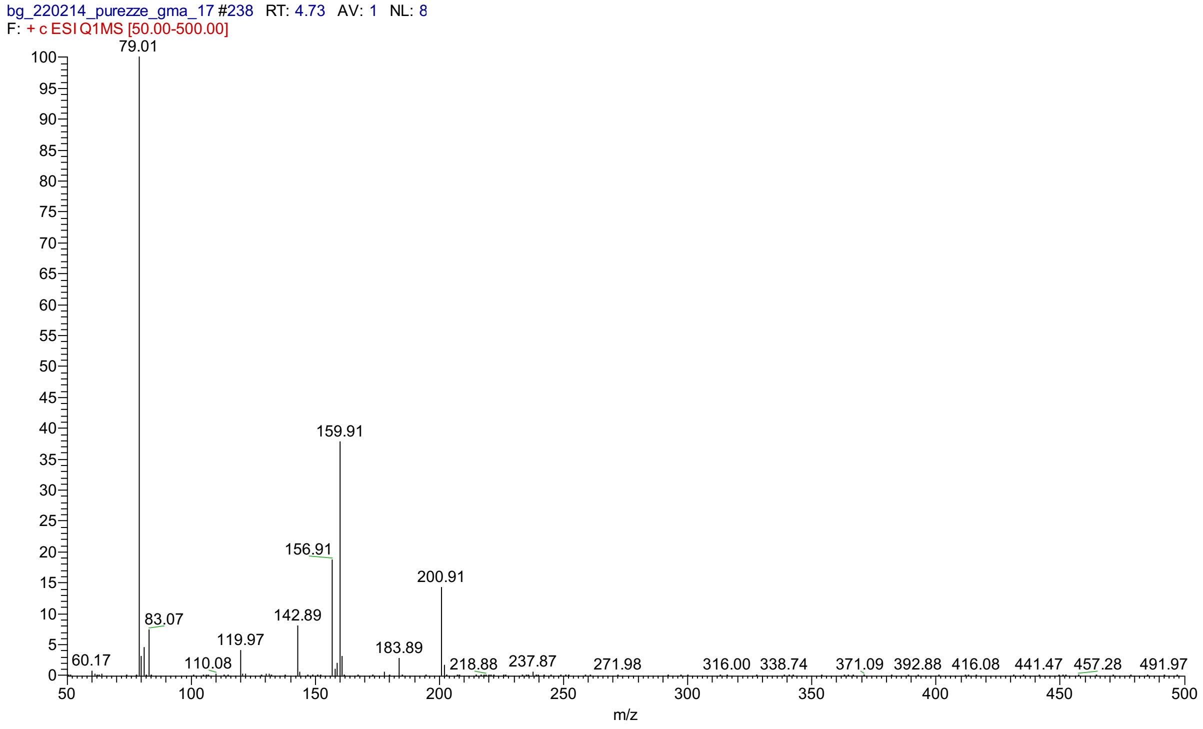

c. Full Scan HPLC Trace in Negative Ion Mode (NIM, ESI¯ mass range 50-500) of vehicle (upper trace) and compound **10d** 100 µM in MeOH+1% DMSO (lower trace).

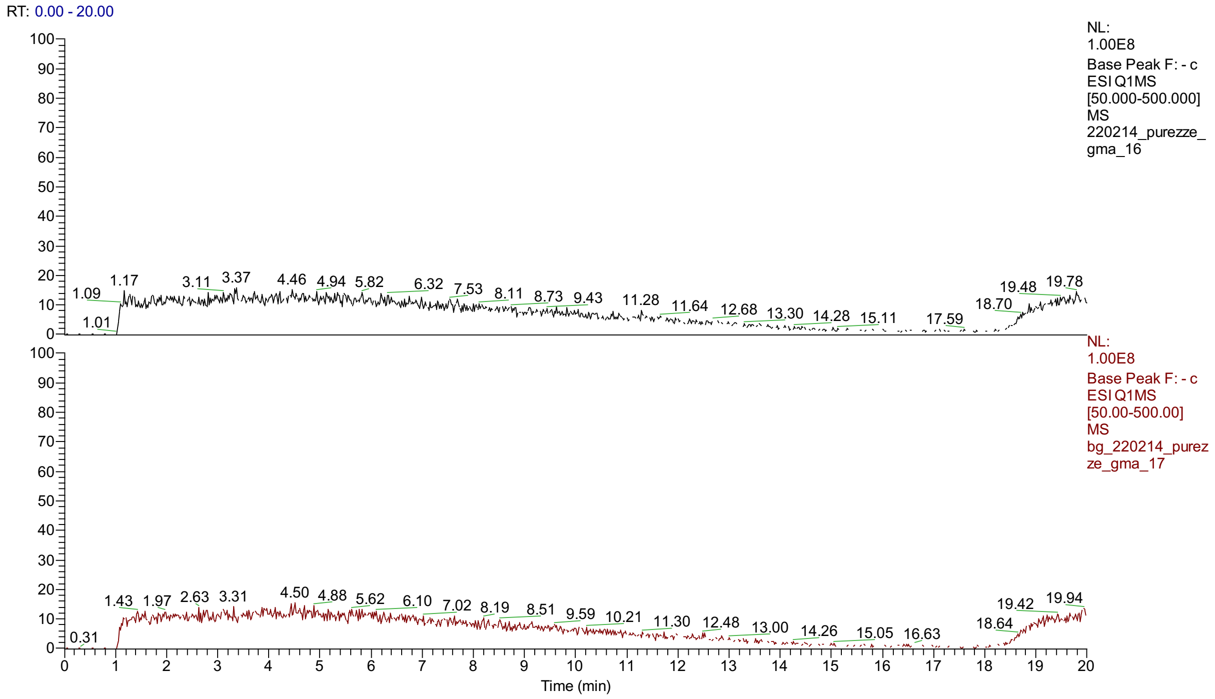

Purity > 99%.
No relevant impurity-related peaks were detected after careful inspection of chromatogram and corresponding full scan MS spectrum in both PIM and NIM.

Annex S7a.Upper panel. Full Scan HPLC Trace in PIM (ESI$^+$ mass range 50-500) of vehicle (upper trace) and compound **10a** 100 µM in MeOH+1% DMSO (lower trace); **Lower panel**. Traces reported in the upper panel zoomed in the retention time window from 5 to 18 minutes.

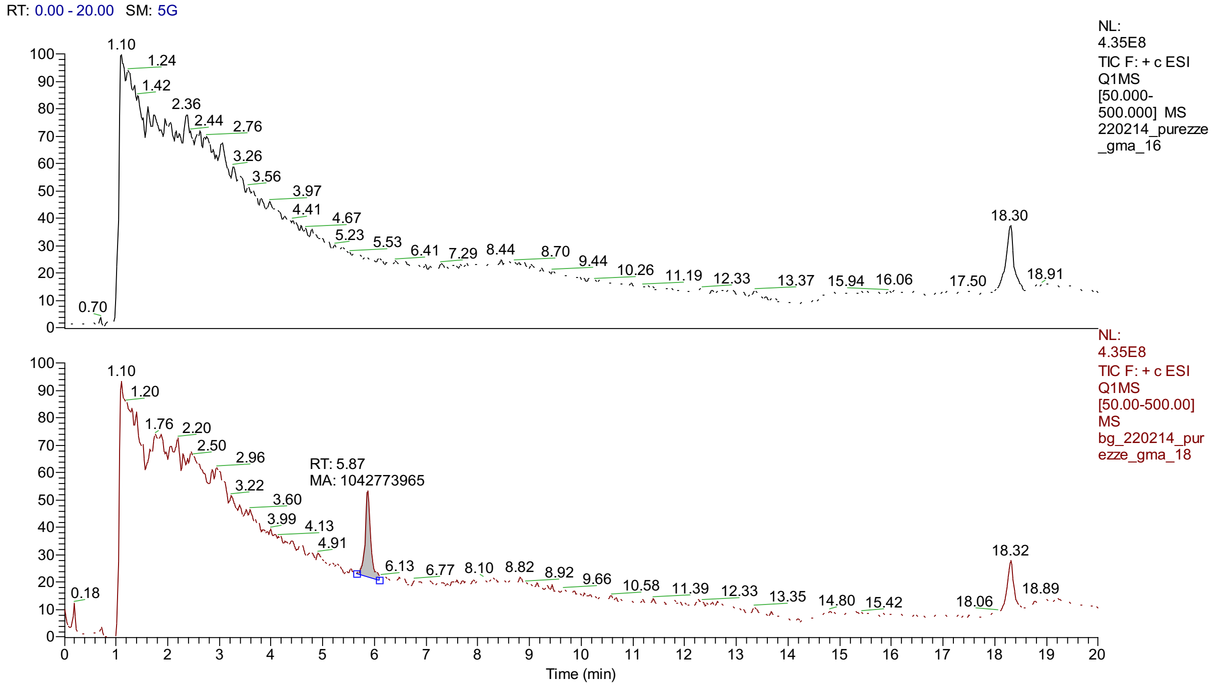

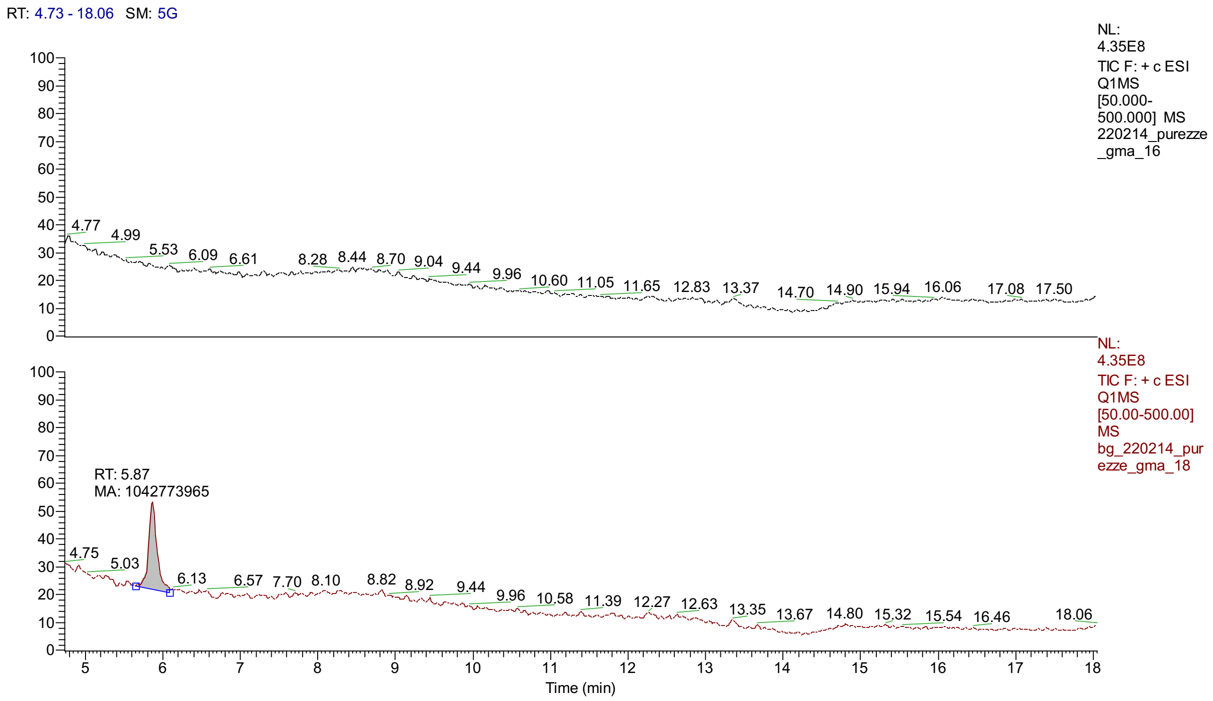

b. MS spectrum of peak at RT=5.87 min.

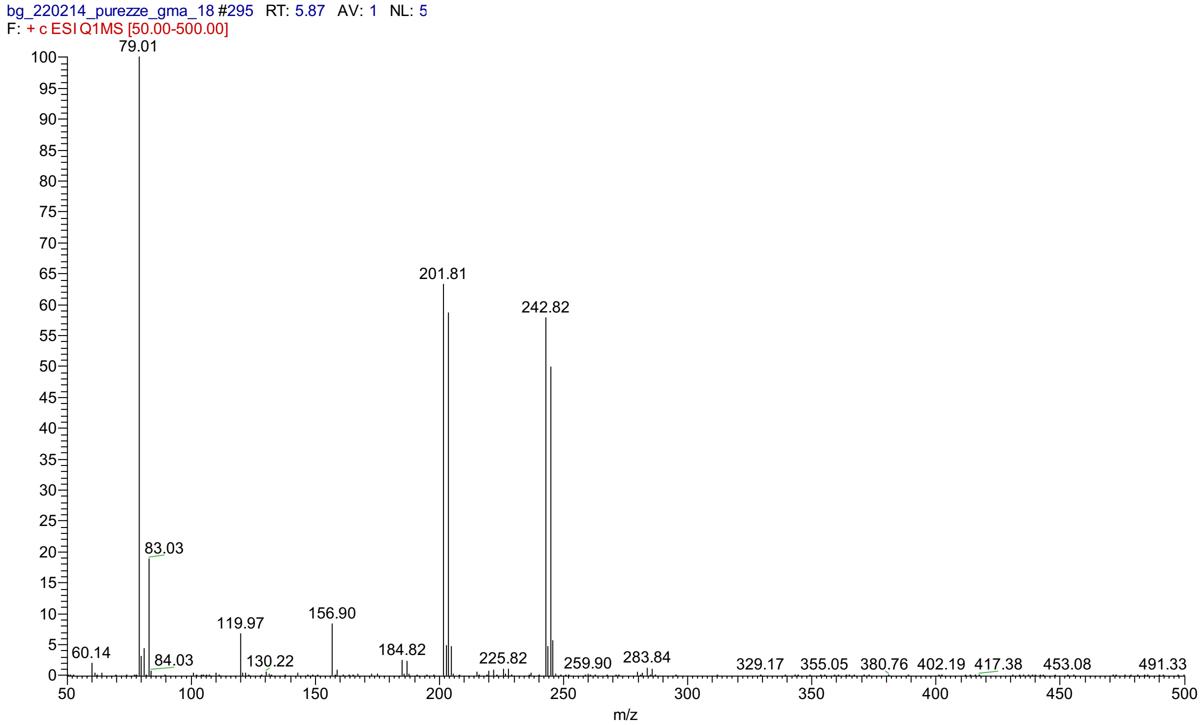

c. Full Scan HPLC Trace in Negative Ion Mode (NIM, ESI$^-$ mass range 50-500) of vehicle (upper trace) and compound **10a** 100 μM in MeOH+1% DMSO (lower trace).

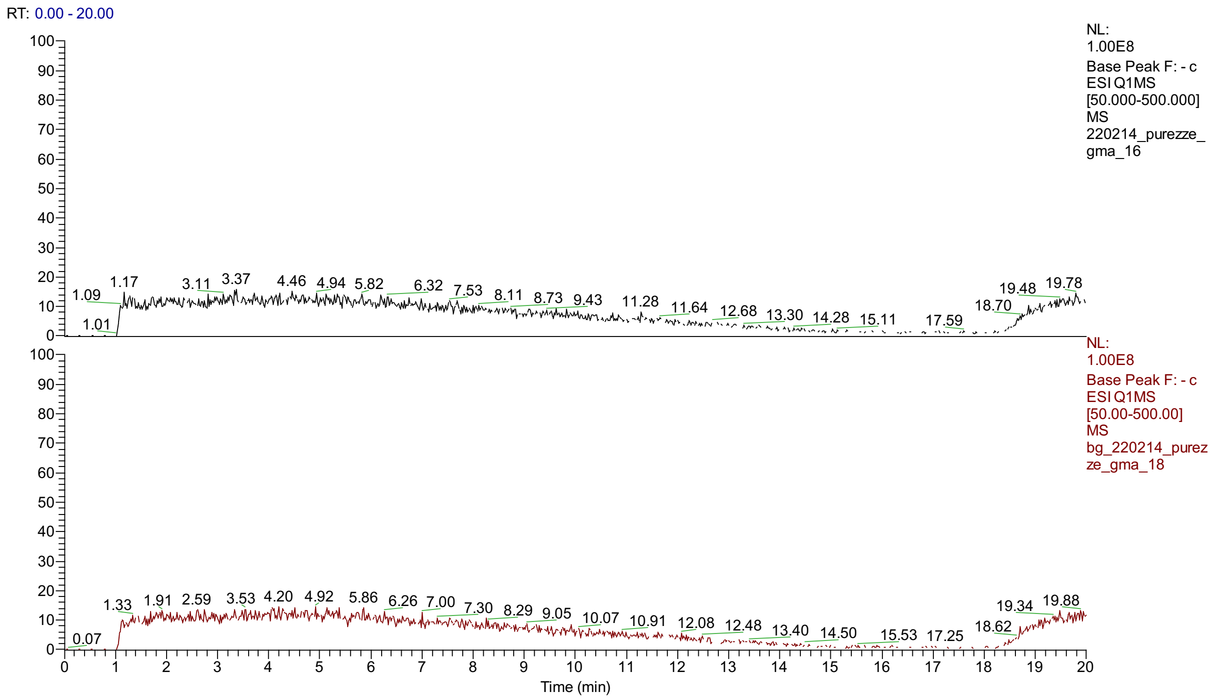

Purity > 99%.
No relevant impurity-related peaks were detected after careful inspection of chromatogram and corresponding full scan MS spectrum in both PIM and NIM.

Annex S8a. Upper trace. Full Scan HPLC Trace in PIM (ESI^+ mass range 50-500) of vehicle (upper trace) and compound **10b** 200 µM in MeOH+2% DMSO (lower trace); **Lower panel**. Traces reported in the upper panel zoomed in the retention time window from 3 to 18 minutes.

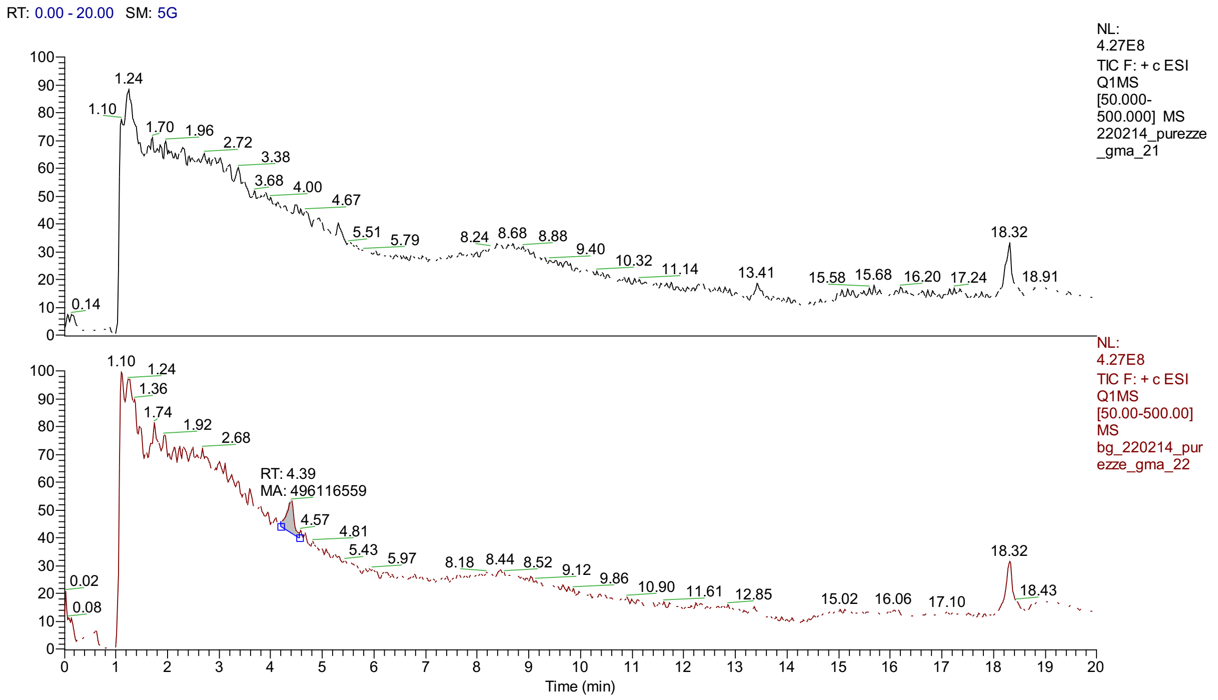

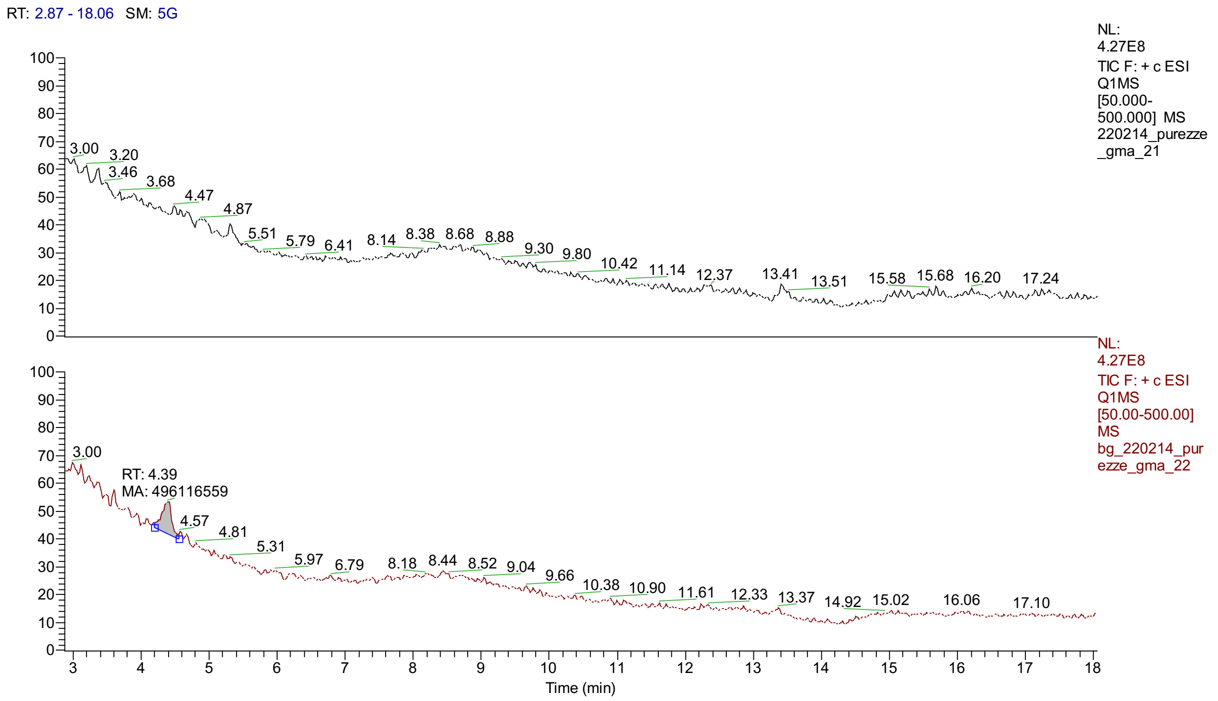

b. MS spectrum of peak at RT=4.39 min.

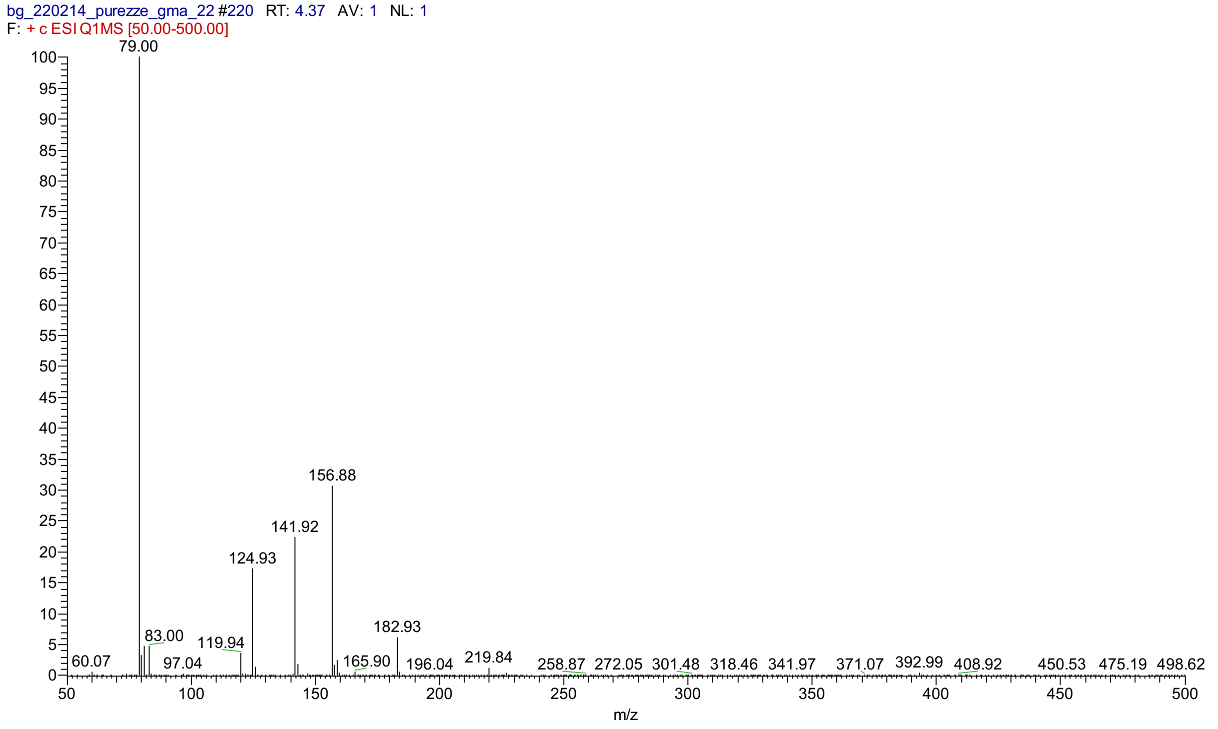

c. Full Scan HPLC Trace in Negative Ion Mode (NIM, ESI^{-} mass range 50-500) of vehicle (upper trace) and compound **10b** 200 µM in MeOH+2% DMSO (lower trace).

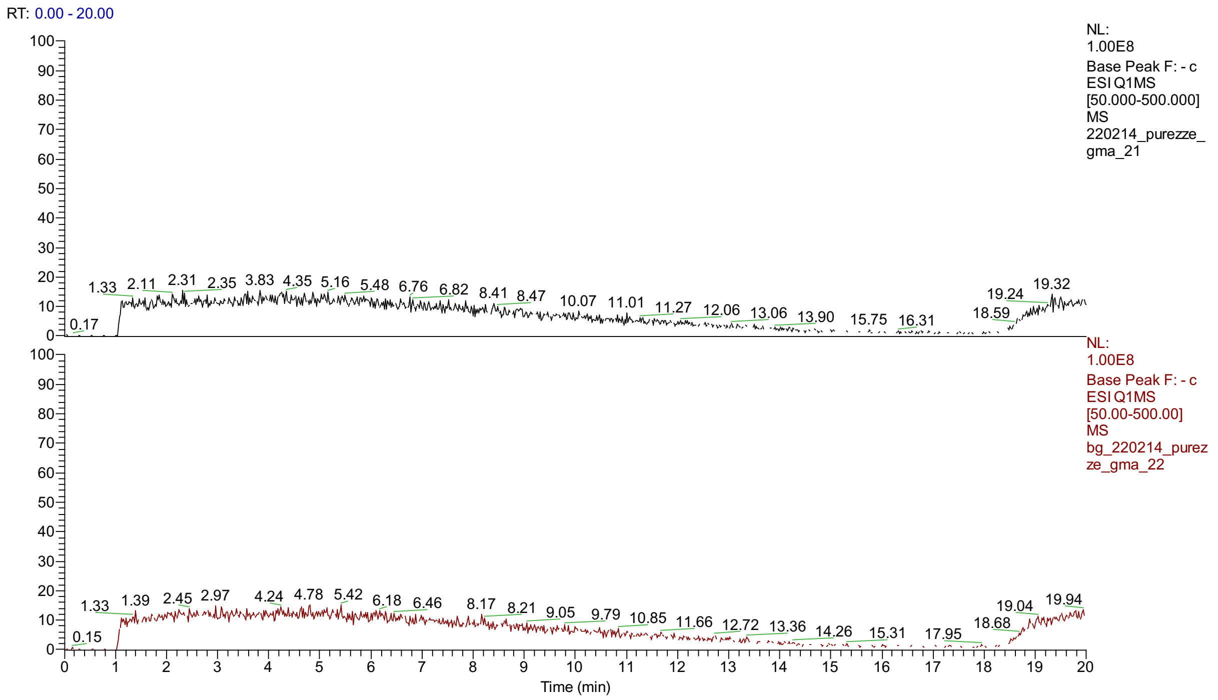

Purity > 99%.

No relevant impurity-related peaks were detected after careful inspection of chromatogram and corresponding full scan MS spectrum in both PIM and NIM.
